# Tear Proteins Calcium binding protein A4 (S100A4) and Prolactin Induced Protein (PIP) are Potential Biomarkers for Thyroid Eye Disease

**DOI:** 10.1038/s41598-018-35096-x

**Published:** 2018-11-16

**Authors:** Chiaw-Ling Chng, Lay Leng Seah, Morgan Yang, Sunny Yu Shen, Siew Kwan Koh, Yan Gao, Lu Deng, Louis Tong, Roger Wilmer Beuerman, Lei Zhou

**Affiliations:** 1 Department of Endocrinology, Singapore General Hospital Level 3, The Academia, 20 College Road, Singapore, 169856 Singapore; 2 Oculoplastic Department, Singapore National Eye Centre 11 Third Hospital Avenue, Singapore, 168751 Singapore; 3 Singapore Eye Research Institute, Discovery Tower Level 6 The Academia, 20 College Road, Singapore, 169856 Singapore; 4 Corneal and External Eye Disease Department, Singapore National Eye Centre 11 Third Hospital Avenue, Singapore, 168751 Singapore

## Abstract

There are no reliable biomarkers to predict thyroid eye disease (TED) in patients with autoimmune thyroid disease (AITD) currently. Several evidences support the involvement of the lacrimal gland in TED. The aim of our study was to quantitatively correlate the changes in tear protein profile with increasing severity of TED. Tear samples were collected from four groups of patients; AITD without TED (AITD), AITD with mild TED (mild TED), AITD with severe TED (severe TED) and normal controls. A total of 72 patients were recruited for the study. In discovery phase, isobaric tags for relative and absolute quantification (iTRAQ) 4-plex was used for quantitative proteomics analysis. For verification of results from discovery phase, sequential window acquisition of all theoretical fragment ion spectra (SWATH) was used to analyze an independent cohort from normal controls, AITD, mild TED and severe TED. Two proteins, S100A4 and PIP showed consistent dysregulation trends in the discovery and validation phase experiments. Our study demonstrated the differences in tear proteome across the spectrum of different severity and activity of TED in patients with AITD. Two tear proteins, S100A4 and PIP may serve as potential biomarkers to predict progression to severe TED in patients with AITD.

## Introduction

Thyroid Eye Disease (TED) is the most common extra-thyroidal manifestation of autoimmune thyroid disease (AITD)^1^. Majority of the presentation is mild, with moderate to severe form affecting 5% of cases, which can progress to sight-threatening disease. Current literature suggests several risk factors for development of TED in patients with AITD, such as smoking and thyroid dysfunction^2^. However, there are no reliable biomarkers currently which could predict development of severe TED in patients with AITD. Consequently, if one could predict the patients in whom significant ocular complications would develop; closer monitoring and appropriate early intervention could be instituted in these susceptible individuals to avoid potential serious consequences.

Tears are complex fluid comprising of secretions from several sources including the lacrimal gland, goblet cells, cornea and vascular sources. Tear composition reflects the physiological condition of the underlying tissues. Thus, tear fluid is useful in the evaluation of health and disease states and represents a valuable source of biomarkers for objective ocular and systemic diseases^3,4^. Potential tear biomarkers have been investigated in several ophthalmic diseases^5^, such as dry eye^6^, keratoconus^7^ and glaucoma^8^. Several evidences support the involvement of the lacrimal gland in the pathogenesis of ocular surface damage in TED. Eckstein *et al*. demonstrated that the acinar cells from lacrimal glands were positive for thyrotropin receptor (TSH receptor) immunoreactivity and thus susceptible to activation by TSH receptor autoantibodies^9^. Enlargement of lacrimal glands have also been reported in clinical and radiological studies in TED patients^10,11^. Recently, this increase in size has been found to be accompanied by changes in tear inflammatory cytokines in TED and correlated with disease inflammatory activity^12^.

Hence, we postulated that tear protein profile can vary with different stages of TED. The aim of our study was to quantitatively correlate the changes in tear protein profile with severity and activity of TED. In this study, quantitative proteomics methods, which were iTRAQ (isobaric tags for relative and absolute quantification) technology coupled with two-dimensional nanoLC-nano-ESI-MS/MS and SWATH-MS (Sequential window acquisition of all theoretical fragment ion spectra), were used to discover and verify candidate biomarkers respectively.

## Materials and Methods

### Subjects

Human tear samples were collected from the following groups of patients: (1) AITD without TED (“group AITD”) (2) AITD with mild inactive TED (group “TED, mild”) (3) AITD with severe active TED (group “TED, severe”) and normal subjects without AITD (group “Ctrl”). Subjects were excluded from the study if they had pterygia, previous ocular surgeries or radiation, concomitant chronic ocular surface inflammation, already been treated with systemic immunosuppressants or had underlying systemic inflammatory conditions or malignancies. In addition, patients receiving medications that could affect tear film constitution (e.g. diuretics, topical eye drops) were also excluded.

A total of 54 subjects with AITD (46 with Graves’ disease and 8 with Hashimoto’s thyroiditis) and 18 control subjects without AITD were recruited from the outpatient clinics for the whole study. All the patients with TED were examined by an Ophthalmologist. The diagnosis of TED was based on diagnostic criteria defined by Bartley and Gorman^13^, i.e. TED is present if eyelid retraction occurs in association with thyroid dysfunction, exophthalmos, optic nerve dysfunction or extraocular muscle involvement and other confounding causes such as idiopathic orbital inflammation are excluded. The subjects’ disease severity was assessed based on ITEDS VISA classification^14^. Briefly, this classification system is based on four disease end points: Vision, Inflammation, Strabismus, and Appearance/Exposure. Subjective input and reproducible objective measurements are recorded for each section, and a global severity grade can be assigned for each function. The subjective and objective progress and tempo of disease can be documented to reflect disease activity. Based on this classification, TED is considered active when the inflammatory score is more than 4/10; mild when only lid signs, proptosis and/or mild extraocular muscle restrictions are present, and severe if vision is affected because of compressive neuropathy, exposure keratopathy or disabling diplopia.

None of the patients were treated with steroids, immunosuppressant (e.g. Methotrexate) or topical eye drops (e.g. steroid or lubricant eye drops) prior to recruitment for the study. Further information on subjects’ autoimmune thyroid disease duration, thyroid receptor antibodies levels (TRAb) and thyroid hormone levels measured at the time of recruitment (for patients with AITD only) or at the time disease presentation (for patients with TED) were collected from electronic medical records.

For the discovery phase, eight patients with underlying AITD without TED (group “AITD”, consists of three males, five females, median age 54.5 yrs, range 46–61, one smoker), eight patients with mild inactive TED (group “TED, mild”, consists of three males, five females, median age 55.5 yrs, range 44–59, one smoker) and eight patients with severe active TED (group “TED, severe”, consists of five males, three females, median age 53.5 yrs, range 46–59, three smokers) were studied. Another eight healthy subjects (five males, three females, and median age 54.5 yrs, range 45–62) served as the normal control group (group “Ctrl”). There were no statistically significant differences in the age, duration of AITD, thyroid hormone levels or TSH receptor antibody titer between the groups in the discovery phase. A separate group of patients were recruited for the verification phase. This group consisted of ten patients with underlying AITD without TED (group “AITD”, consists of two males, eight females, median age 59.5 yrs, range 43–67, no smokers), ten patients with mild inactive TED (group “TED, mild”, consists of two males, eight females, median age 53 yrs, range 40–63, one smoker) and ten patients with severe active TED (group “TED, severe”, consists of six males, four females, median age 58.5 yrs, range 43–86, two smokers). Another ten healthy subjects (group “Ctrl”, consists of three males, seven females, median age 61.5 yrs, range 53–72) were recruited as normal controls. There were also no statistically significant differences in the age, thyroid hormone levels or TSH receptor antibody titer between the groups in the verification phase. The patients with severe TED had significantly shorter duration of AITD compared to patients with AITD only (p = 0.010) and mild TED (p = 0.029).

Informed consent was obtained from all participating subjects and the study was approved by the SingHealth Centralized Institutional Review Board. The current study was performed in accordance with the Declaration of Helsinki. The clinical characteristics of subjects recruited in the discovery and validation stages are summarized in Table 1 and Table 2 respectively.

**Table 1:** Clinical characteristics the subjects with AITD in the discovery phase.

Clinical characteristics	AITD only (n = 8)	TED, mild (n = 8)	TED, severe (n = 8)	Controls (n = 8)
Male, n (%)	3 (37.5)	3 (37.5)	5 (62.5)	5 (62.5)
Age, yrs, median (range)	54.5 (46–61)	55.5 (44–59)	53.5 (46–59)	54.5 (45–62)
Smokers, n (%)	1 (12.5)	1 (12.5)	3 (37.5)	
Duration of AITD, mth, median (range)	18 (1–504)	18 (0–204)	12.5 (0–19)	
Graves’ disease	4 (50)	8 (100)	8 (100)	
CAS (/10), median, range	NA	0.5 (0–1)	5.5 (4–8)	
TRAb, IU/L (reference < 1.5), median (range)	6.2 (0.8–38.1)	4.3 (1.4–19.7)	6.4 (1.6–40)	
FT4, pmol/L (reference 8.8–14.4), median (range)	14.6 (9.3–34.3)	13.2 (9.1–40.2)	11.1 (6.7–15.8)	
TSH, mIU/L (reference 0.65–3.70), median (range)	1.871 (0.033–3.14)	1.37 (0.015–4.33)	0.227 (0.005–2.13)

**Table 2:** Clinical characteristics of the subjects in the verification phase.

Clinical characteristics	AITD (n = 10)	TED, mild (n = 10)	TED, severe (n = 10)	Controls (n = 10)
Male, n (%)	2 (20)	2 (20)	6 (60)	3 (30)
Age, yrs, median (range)	59.5 (43–67)	53.0 (40–63)	58.5 (43–86)	61.5 (53–72)
Smokers, n (%)	0	1 (10)	2 (20)	
Duration of AITD, mth, median (range)	48 (7–480)	31.5 (6–252)	5.5 (1–168)	
Graves’ disease	7 (70)	9 (90)	10 (100)	
CAS (/10), median, range	NA	0 (0–3)	5 (4–7)	
TRAb, IU/L (reference < 1.5), median (range)	1.9 (0.7–40)	3.4 (0–16.3)	3.8 (0.8–13.8)	
FT4, pmol/L (reference 8.8–14.4), median (range)	12.2 (10.3–16.7)	13.4 (5.4–25.2)	14.0 (9.7–29.5)	
TSH,mIU/L (reference 0.65–3.70), median (range)	1.68 (0.067–3.07)	0.98 (0.004–3.59)	0.223 (0.006–3.69)	

### Tears collection and tear protein elution

Tear fluid was collected with gloved hands from each eye using Schirmer’s paper strips. These strips were inserted in the inferior conjunctival cul-de-sac (fornix) 1 cm from the lateral canthus for five minutes with eyes open and in a dim room. Special care was taken not to touch the eyelids or ocular surface to avoid eye irritation and contamination^15^. No stimulation, anesthetic or eye drops were used before sample collection. External factors known to affect the content of tear samples, such as harsh lighting, background noise and extremes of room temperature were strictly avoided. After collection, Schirmer’s strips were immediately frozen at −80 °C until further analysis. The Schirmer’s strips were cut into small pieces and soaked in 100 µL of 50-mM ammonium bicarbonate (Sigma-Aldrich, St. Louis, MO, USA) solution containing protease inhibitor (Halt Protease Inhibitor cocktail, Thermo Scientific, Rockford, IL, USA) for three hours to elute tear proteins. Total tear protein concentration of each sample was measured using a Micro BCA Protein Assay Kit (Pierce Biotechnology, Inc.).

### Study design and iTRAQ sample preparation

**Figure 1:**
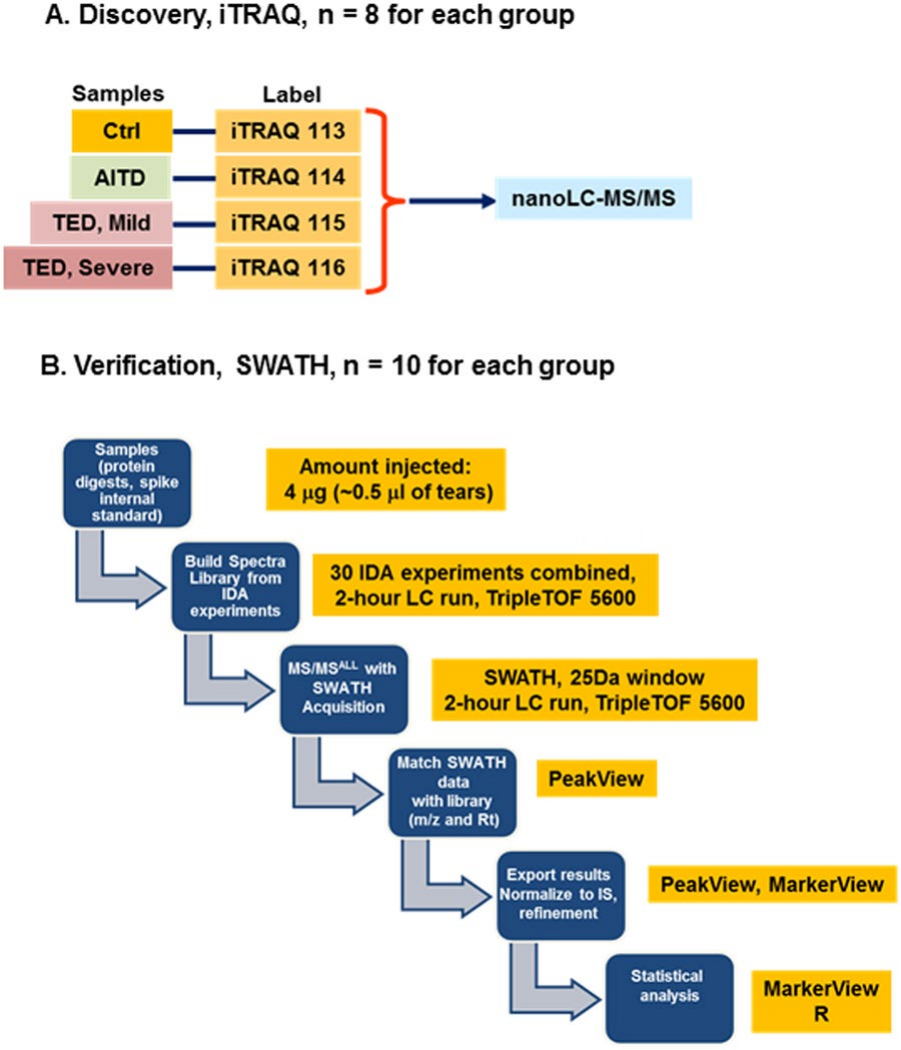
Experimental design. (**A**) Discovery phase: 8 sets of 4-plex iTRAQ experiments were performed, 8 tear samples were used in each disease group (healthy control, AITD, mild TED and severe TED). (**B**) Verification phase: SWATH was used for analysis of tear samples from an independent patient cohort including healthy control, AITD, mild TED and severe TED; 10 tear samples for each group.

The overall experimental design is given in Fig. 1. In the discovery phase, iTRAQ 4-plex was used for quantitative proteomics analysis. One iTRAQ 4-plex set included one sample each from control, AITD, TED (mild) and TED (severe) and with a total of eight iTRAQ sets (Fig. 1A). To verify the results from the discovery phase, Sequential window acquisition of all theoretical fragment ion spectra (SWATH) was used to analyze an independent cohort which consisted of 10 samples each from controls, AITD, TED (mild) and TED (severe). Two technical replicates were performed from each sample.

For each sample, 100 µg total protein amount was used. Briefly, protein samples were reduced using final concentration of 5 mM TCEP (Sigma, MO, USA) and incubated for one hour at 60 °C. This was followed by alkylating the reduced protein sample with 15 mM IAA (final concentration) for 20 minutes in the dark. The sample was then digested with trypsin (ABSciex, MA, USA) at a trypsin-to-substrate ratio of 1:50, at 37 °C overnight. Finally, the digested samples were reconstituted in dissolution buffer (0.5 µM TEAB) and labeled with iTRAQ reagents according to the experimental design in Fig. 1A.

NanoLC-MS/MS [Ultimate 3000 nanoLC system (Dionex, Thermo Fisher Scientific, MA, USA) coupled with AB Sciex 5600 TripleTOF (AB Sciex, Framingham, MA, USA)] was used for the proteomic analysis. The LC separation is based on the reversed phase LC with an Acclaim PepMap RSLC C18 column (15 cm × 75 µm i.d., Dionex, Thermo Fisher Scientific, MA, USA). A step linear gradient of mobile phase B (2/98 v/v of water/ACN with 0.1% formic acid) from 5% to 12% over 30 min, 12–40% for 60 min, and lastly, 40–90% over 20 min at flow rate of 300 nL/min was used for the peptide separation. Two microliters of sample were injected for the analysis. Proteomics data was acquired using information-dependent acquisition (IDA) mode using Analyst TF 1.7 software (AB Sciex, USA). Data acquisition parameters were listed as follows, (1) TOF-MS survey scan: 250 ms; (2) mass range for TOF-MS: 400–1250; (3) product ion scan: 50 ms; (4) mass range for MS/MS: of 100–1500; (5) switching criteria: ions greater than m/z 400 and smaller than m/z 1250 with charge state of 2–5, maximum number of candidate ions to monitor per cycle was 40 spectra and an abundance threshold of >120 counts; former target ions were excluded for 12 s; (6) collision energy (CE): IDA Advanced “rolling collision energy”.

The iTRAQ data was processed with ProteinPilot 5.0 (AB Sciex, Framingham, MA, USA) using protein database version “uniprot_all_Oct2014”.

### Verification phase using SWATH

#### Generation of spectral library using IDA mass spectrometry

To establish the human tear spectral library (Fig. 1B), a global control tear sample pooled from 1000 healthy subjects (411 male, 589 female, average age 55.5 years, SD 14.5 years) was used. For this purpose, 200 µg of tear protein sample was denatured with 0.1% Rapigest (Waters, MA, USA) to a final concentration of 0.025%. The protein samples were denatured, alkylated and tryptic digested as described above. The digested sample was spun down and 10% formic acid (final concentration) was added and mixed for 45 min at 37 °C. The acid-treated sample was subsequently spun down at 13000 rpm for 10 min to precipitate out hydrolytic Rapigest by-products. Next, the peptide sample was desalted using ultramicrospin Silica C18 column (The Nest Group, Inc., MA, USA). Finally, the eluted peptide sample was lyophilized for further analysis. To build the comprehensive human tear spectral library, 30 IDA runs were combined.

The experimental conditions for nanoLC-MS/MS analysis were the same as the above.

#### SWATH-MS (DIA mass spectrometry)

The LC set-up and experimental parameters are the same as the above. For SWATH experiment (Fig. 1B), the instrumentation settings were as follows: Ionspray Voltage Floating (ISVF) = 2400 V, curtain gas (CUR) = 30, Ion source gas 1 (GS1) = 12, Interface Heater Temperature (IHT) = 125, Declustering potential (DP) = 100 V. All data was acquired using SWATH mode with Analyst TF 1.7 software (AB Sciex). TOF-MS scan (experiment 1) parameters were set as follows: 0.05 sec TOF MS accumulation time in the mass range of 350–1250 Da followed by SWATH parameters as follows: 0.075 sec accumulation time in the mass range of 350–1250 Da with a swath width of 25 Da. The product ion mass range was 100–1500 Da, using high sensitivity mode and the collision energy spread was set at 15 V.

#### Data analysis and Statistical Analysis

For between group comparisons, Mann-Whitney U test and Kruskal-Wallis tests were used to compare medians. For iTRAQ data, geometric means of technical replicates were used for further analysis. The average ratios of AITD/Ctrl, TED (mild)/Ctrl and TED (severe)/Ctrl from eight iTRAQ sets were calculated using geometric mean. The p-values were generated using t-test of log transformed data. For SWATH data (Fig. 1B), alignment and normalization of the raw data was performed using SWATH data processing package in PeakView (AB Sciex, Framingham, MA, USA). Data was exported for further analysis (Fig. 1B) using *R* (Foundation for Statistical Computing, Vienna, Austria).

## Results

**Figure 2:**
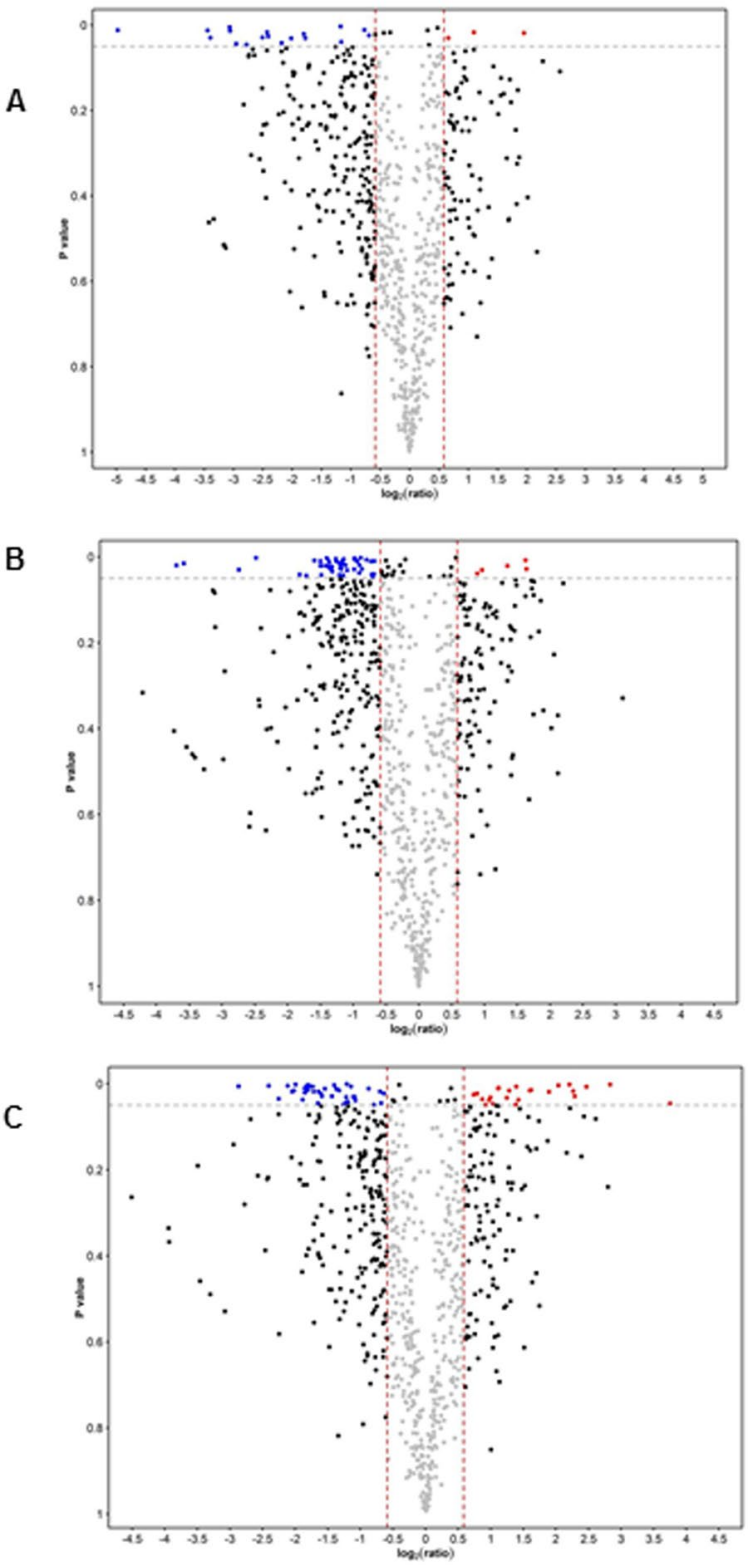
Volcano plots for discovery phase. The cutoff for significant changes: Fold-change of 1.5 and p < 0.05. (**A**) AITD vs Ctrl. (**B**) mild TED vs Ctrl. (**C**) Severe TED vs Ctrl.

In the discovery phase using iTRAQ quantitative proteomics, we identified 1167 unique tear proteins with false discovery rate (FDR) < 1% (95% peptide confidence level). Using a specific cut-off of significant fold change, defined as fold change >1.5 for up-regulation and <0.67 for down-regulation (p < 0.05), we found three up-regulated and 18 down-regulated tear proteins when tear protein profile was compared between AITD and normal controls (Fig. 2A, Supplemental Table S1). There were five up-regulated and 46 down-regulated tear proteins in mild TED vs controls (Fig. 2B, Supplemental Table S2), and 24 up-regulated and 44 down-regulated tear proteins in severe TED vs controls (Fig. 2C, Supplemental Table S3), respectively.

To verify the results from discovery proteomics using SWATH, it is necessary to establish a comprehensive spectral library to process SWATH data. The tear sample used for generation of spectral library was pooled from 1000 healthy subjects (male: female = 51.1%: 48.9%) to avoid biasness from individual samples. Thirty 1D LC-MS/MS using IDA mode were combined. The total number of unique proteins identified was 1508 (FDR < 1%, 95% peptide confidence level) after combining 30 IDA runs (Supplemental Table S4). The average number of unique proteins identified was 826 ± 18 (610 ± 14 with ≥2 peptides) for a single run at FDR < 1%. This represents the most comprehensive spectral library for human tears.

**Figure 3:**
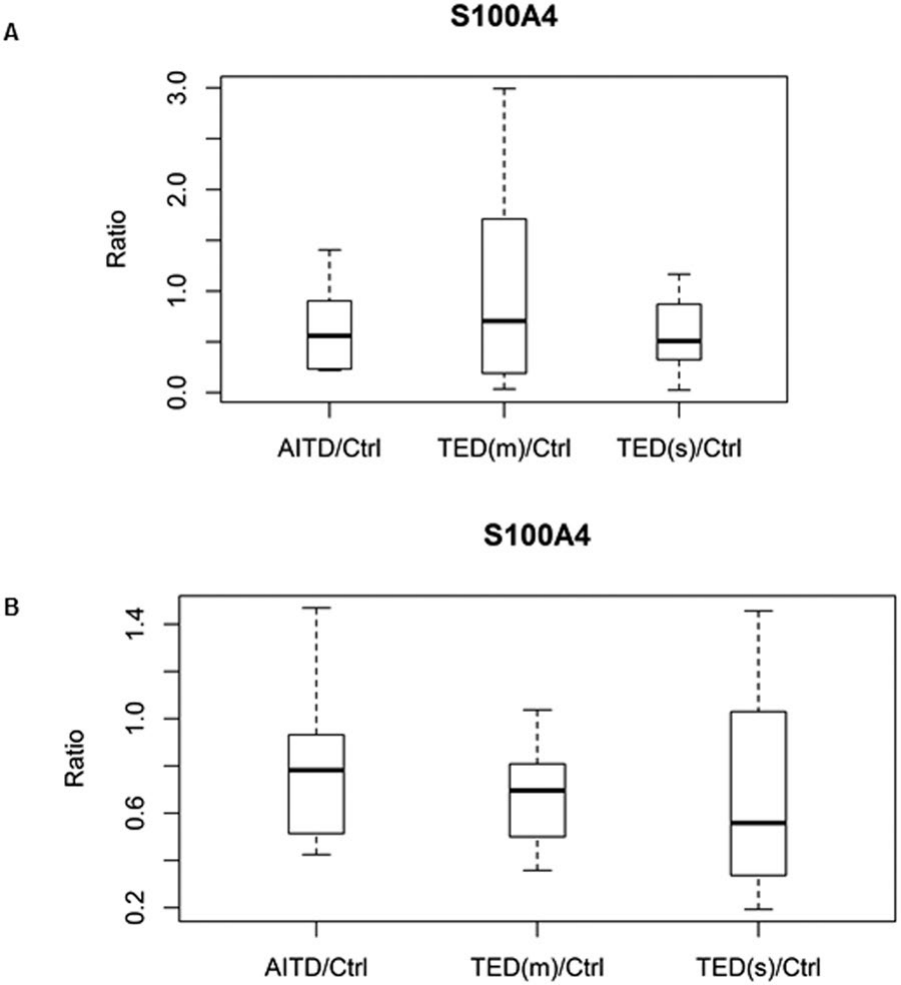
Box-and-whisker plots of S100A4. (**A**) Discovery phase by iTRAQ [TED(s)/Ctrl, p = 0.029]. (**B**) Validation phase by SWATH [TED(m)/Ctrl, p = 0.0022, TED(s)/Ctrl, p = 0.0361].

**Figure 4:**
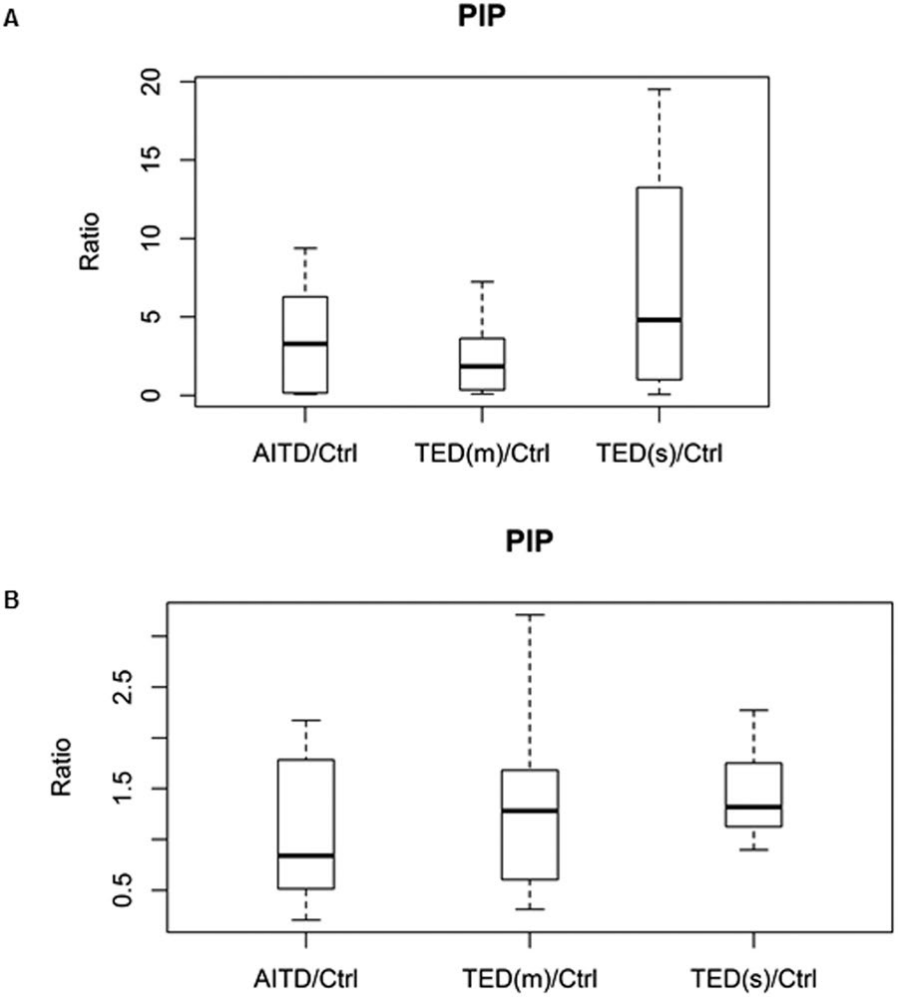
Box-and-whisker plots of PIP. (**A**) Discovery phase by iTRAQ [TED(s)/Ctrl, p = 0.023]. (**B**) Validation phase by SWATH [TED(s)/Ctrl, p = 0.0131].

After statistical analysis, Clusterin (CLU) [TED(m or mild)/Ctrl, p = 0.030, TED(s or severe)/Ctrl, p = 0.002], Mesothelin (MSLN) [TED(m)/Ctrl, p = 0.028, TED(s)/Ctrl, p = 0.023], and Prolactin Induced Protein were up-regulated [TED(s)/Ctrl, p = 0.023] and S100 Calcium binding protein A4 (S100A4) was down-regulated [TED(s)/Ctrl, p = 0.029] in the discovery phase. The dysregulation trends of two of these tear proteins, S100A4 and PIP, were confirmed on the verification phase (Figs 3 and 4). There was an observed downward trend of S100A4 protein fold change and an observed upward trend of PIP fold change with increasing severity of TED in both discovery and verification phase experiments. Fold change and p-values of S100A4 and PIP in both discovery and verification phases are provided in Table 3.

**Table 3:** Fold change and p values of S100A4 and PIP in both discovery and verification phases.

Protein	AITD/Ctrl FC	AITD/Ctrl p-value	TED(m)/Ctrl FC	TED(m)/Ctrl p-value	TED(s)/Ctrl FC	TED(s)/Ctrl p-value
**Discovery Phase**						
S100A4	0.63	0.280	0.49	0.227	0.33	0.029*
PIP	1.05	0.954	1.48	0.551	4.19	0.023*
**Verification Phase**						
S100A4	0.73	0.058	0.65	0.002*	0.55	0.036*
PIP	0.83	0.874	1.09	0.237	1.37	0.013*

*p < 0.05, FC: Fold change.

## Discussion

The use of tears as a source of biomarker discovery is an approach of pivotal importance. Protein profiling of tears reflects the altered states in the eye due to the close spatial relations of the lacrimal gland. Previous evidence supports lacrimal involvement in TED. Recent studies have revealed different tear proteome composition between patients with TED and healthy controls^16–18^, AITD without TED^19^, dry eye syndrome^20^ and smokers^21^. The older studies utilized low resolution proteomic technique such as HPLC and 1D gel electrophoresis, for identification of the protein markers. Recently, Matheis *et al*. have analyzed tears from 120 subjects with TED and correlated this pathology with subjects with dry eye syndrome in a two-phase experimental design. In the discovery phase, PROL1, S100A8, Annexin A1 and PRP4, among others, were dysregulated in healthy individuals. In the second phase, the authors performed an antibody microarray based validation and confirmed significant changes between the levels of PRP4, PRO1 and UGDH in TED and dry eyes and healthy controls^20^. In their study, MALDI-TOF/TOF was used for protein identification, which is different from our LC-ESI-MS/MS. Due to different proteomics platform used, there could be study-specific biomarkers identified in each study^22^. However, in our current study, the two biomarker candidates were identified from two independent cohorts using two different techniques (iTRAQ and SWATH).

In our study, tears were collected using Schirmer’s strips. Tear proteins were then eluted from the strip by 50-mM ammonium bicarbonate using the same protocol for each sample. Prior work by Grus *et al*. and our experience using this method showed that the recovery of total tear protein was reproducible and acceptable^23^. Our study is the first to compare the tear proteome across different severity of TED using highly sensitive quantitative proteomics approach. We employed iTRAQ technology coupled with two-dimensional nanoLC-nano-ESI-MS/MS in the discovery phase. The iTRAQ technique is widely used for discovery-based proteomics, which allows for simultaneous protein identification and (relative) quantification. We verified the results from discovery phase (using iTRAQ on data-dependent acquisition mode) by SWATH (data-independent acquisition mode) on an independent cohort. In addition to traditional antibody-based targeted assays (ELISA or western blot), mass spectrometry (MS)-based proteomic technologies, such as multiple reaction monitoring (MRM), parallel reaction monitoring (PRM), and Sequential Windowed Acquisition of All Theoretical Fragment Ion Mass Spectra (SWATH-MS) have been used as powerful targeted MS-based methods for verification of the results gained from discovery-phase proteomics studies^24,25^. The capability of multiplexing in targeted MS-based is especially suitable for samples with extremely low volume, such as tears. In our study, we demonstrated that a few hundred tear proteins can be quantified in one analysis using SWATH-MS. The two different quantitative approaches (iTRAQ and SWATH) have been shown to complement each other, as for a given instrument time, the iTRAQ experiment will provide more protein identifications; however, the SWATH experiment can identify more differential ratios, being both more sensitive in the detection of small differential ratios and being able to cover a larger dynamic range^26^.

The discovery phase experiments demonstrated four tear proteins were differentially expressed between varying severity of TED and normal control group; with three up-regulated (CLU, MSLN and PIP) and one down-regulated (S100A4). The dysregulation of two proteins (S100A4 and PIP) showed similar trends in the verification phase experiments and reached statistical significance. S100A4 is a member of the S100 calcium binding protein family. Through binding with different proteins, S100 proteins are involved in the regulation of many important cellular activities such calcium homeostasis, cytoskeleton organization, stress response, cell motility, cell proliferation and differentiation^27^. Expression of S100A4 in different tumor cells has been shown to strongly correlate with aggressive metastatic tumor phenotype. Recently, increased expression of this protein has been implicated in pathologies of non-malignant diseases such as rheumatoid arthritis and disorders in cardiovascular, nervous and pulmonary systems. Interestingly, it is postulated that the common feature of various diseases implicating S100A4 is its participation in the inflammatory component which contribute to the development and progression of the various pathologies^28^. However, our study results did not support this since S100A4 was down-regulated with worsening severity of TED. Recent studies have linked S100 positivity in the ocular surface tissue in non-inflammatory conditions^6,29,30^. Notably, selective S100 proteins involvement in corneal epithelial cell proliferation and differentiation under normal and pathological conditions was suggested by a study by Jing *et al*.^31^. Nubile *et al*. demonstrated strong cytoplasmic expression of the total S100 family (including S100A4) in all limbal epithelial crypts (LECs) of healthy corneas and marked reduced expression was noted in an inflamed limbus^32^. In particular, S100A4 protein presented higher limbal expression in healthy samples. The LEC are anatomically well defined structures and are located deeper in the substantia propria of the limbus providing both protection and a microenvironment of extracellular matrix with its multitude of resident cells^33^. Based on the results of their study, the authors concluded that the drastic reduction of S100 expression, highlighted in limbal inflammation, showed different behavior of this family of proteins in limbal tissues compared to other forms of ocular inflammation in which S100 over-expression was reported. These evidence supports the hypothesis that S100 proteins represent a marker of limbal normality^32^.

Superior limbic keratoconjunctivitis (SLK) is characterized by inflammation of the superior bulbar conjunctiva with predominant involvement of the superior limbus and adjacent epithelial keratitis and has been associated with TED^34^. The precise cause of SLK in TED is unknown, but has been thought to be due to mechanical factors such as the oculopalpebral asynergy or related to an autoimmune mechanism^35^. In an earlier study by Kadrmas *et al*., SLK was noted to be prognostic sign for severe TED^36^. No studies to date examine in detail the pathological changes of the limbus in patients with TED at different stages of their disease. Although up to a third of SLK cases are linked to TED, the diagnosis of SLK in TED patients is uncommon^34^. The expression of S100A4 protein is also not exclusive to the limbus and may be found in other ocular surface tissues^37^. We did not ascertain the incidence of SLK in our subjects in this study. Hence, whether increasing limbal inflammation due to the autoimmune process in TED contributed to the downward trend of S100A4 protein observed in the tear profile in our patients reflects the increasing severity and activity of their orbital condition remains to be elucidated in further studies.

Prolactin-inducible protein (PIP)/gross cystic disease fluid protein-15 (GCDFP-15) is typically expressed in several exocrine tissues, such as lacrimal, salivary, and sweat glands. Recently, PIP has been investigated as a potential biomarker for metastatic breast cancer and keratoconus^38,39^. Although the function of PIP is not well elucidated, recent evidence suggest that its primary role may be related to host defense and immune modulation^40^. Several studies have shown that PIP has a strong affinity for the CD4 receptor and is a specific inhibitor of CD4+ T-cell receptor-mediated apoptosis, with potential functions in innate and adaptive immunity^40^. PIP expression has also been shown to be influenced by several cytokines, namely IL4/13, IL1 and IL6^39^. Since aberrant cytokine expression and breakdown of immune tolerance is central to pathogenesis of TED^41^, we postulate the changes in PIP in our study could reflect these processes. PIP has recently been reported as a biomarker on keratoconus in human studies^39,42^. The fact that PIP was downregulated in keratoconus disease but upregulated in TED in our study further strengthens the specificity of our study results. Interestingly, in the study on the role of PIP as a potential biomarker for keratoconus disease, Priyadarsini *et al*. noted PIP expression was up-regulated in both healthy and diseased human corneal fibroblasts upon Transforming Growth Factor β (TGF-β) treatment^43^. Orbital fibroblast is central to the pathogenesis of TED and the cytokine TGF-β plays a key role in this autoimmune inflammatory disorder of the orbit. Two subpopulations of orbital fibroblasts were previously noted; Thy1− (CD90−) orbital fibroblasts which exhibit high capacity to differentiate into adipocytes, and Thy1 +(CD90+) orbital fibroblasts which tend to differentiate into α–smooth muscle actin expressing myofibroblasts. When exposed to TGF-β, the Thy-1- fibroblasts differentiate into myofibroblasts with prominent cytoplasmic actin filaments that can participate in inflammation, repair, and fibrosis^44^. Within the orbit, relative proportions of Thy-1+ and Thy-1− fibroblasts and their degree of exposure to TGF-β may affect disease expression, including whether muscle or fat expansion predominates and the extent of fibrosis that develops^45^. The presence of TGF-β, particularly in patients with severe TED, potentially augments PIP levels in the tears of these patients.

It is interesting to note that the dysregulation trend of both S100A4 and PIP is opposite in TED compared to our earlier works which compared dry eyes and healthy controls^6^. Dry eye is the most frequent cause of the ocular discomfort in TED and has been found to be present in 85% of patients^46,47^. The underlying mechanism is not fully elucidated but was thought to be related to anatomical alterations in TED such as corneal exposure from exophthalmos and lid retraction leading to ocular surface drying, ocular surface inflammation, as well as lacrimal gland dysfunction in TED^48,49^. In contrast, the cause of dry eye syndrome is fundamentally due to an insufficiency of tears to maintain the ocular surface. Previous proteomic studies have showed distinctly different proteomics pattern between TED and dry eye syndrome^20^.

The main limitation of our study is the small sample size. However, all the subjects with TED recruited for the study were not treated (e.g. with steroids, immunosuppressant, topical eye drops) prior to collection of tear samples, since treatment of TED could potentially alter the tear proteome composition. This stringent criterion nonetheless affected the patient numbers that could be recruited for the study. Although the patients with concomitant medical conditions or receiving medications that could affect the tear film constituents were excluded from the study, we cannot be completely sure other medical conditions of the subjects would not influence the levels of the S100A4 and PIP.

## Conclusion

Our study demonstrated for the first time, differences in tear proteome across the spectrum of different severity and activity of TED in patients with AITD. Of note, two proteins, S100A4 and PIP showed consistent dysregulation trends in the discovery and validation phase experiments. These tear proteins may serve as potential biomarkers to predict progression to severe TED in patients with AITD. Based on previous studies, S100A4 may represent limbal normality and PIP could reflect the breakdown of immune tolerance and ongoing fibrosis in severe TED. Hence, these potential biomarkers appear to complement each other, with S100A4 protein down-regulated in both mild inactive and severe TED, potentially providing a marker for the propensity to develop TED in patients with AITD before clinical manifestation; while PIP up-regulation may signify more ominous progression to severe TED. Further prospective evaluation on larger cohort of patients would be necessary to confirm our findings.

## Electronic supplementary material

Supplementary Tables S1-S4 accompanies this paper.

## Data Availability

Any additional data beyond those included in the main text that supports the findings of this study are also available from the corresponding author upon request.
