# Supplementary material for: Tear Proteins Calcium binding protein A4 (S100A4) and Prolactin Induced Protein (PIP) are Potential Biomarkers for Thyroid Eye Disease
Source: Sci Rep. 2018 Nov 16;8:16936. doi: 10.1038/s41598-018-35096-x (PMC6240106; doi:10.1038/s41598-018-35096-x)
Supplement: Supplementary file 1 — Supplementary Tables S1-S4 accompanies this paper. [file 41598_2018_35096_MOESM1_ESM.pdf]

Title: Tear Proteins Calcium binding protein A4 (S100A4) and Prolactin Induced Protein (PIP) are Potential Biomarkers for Thyroid Eye Disease

Authors: Chiaw-Ling Chng, Lay Leng Seah, Morgan Yang, Shen Yu Sunny, Siew Kwan Koh, Yan Gao, Lu Deng, Louis Tong, Roger Wilmer Beuerman, Lei Zhou

**Up regulated proteins**		
**Gene Symbol**	**Fold Change (geometric mean)**	**p-value**
ALDH3A1	3.972	0.020
RARRES1	2.193	0.019
CHMP4B	1.705	0.013
**Down regulated proteins**		
**Gene Symbol**	**Fold Change (geometric mean)**	**p-value**
TF	0.092	0.003
IGHG4	0.095	0.013
ALB	0.120	0.014
LTF	0.126	0.003
APOA2	0.128	0.046
SERPINA1	0.139	0.006
A1BG	0.164	0.035
APOA1	0.173	0.043
PLA2G2A	0.178	0.040
AGR2	0.184	0.030
ORM1	0.188	0.027
HPX	0.206	0.015
GC	0.216	0.019
PRDX5	0.271	0.036
ITIH1	0.398	0.046
LAP3	0.447	0.043
SERPINC1	0.468	0.05
AHSG	0.530	0.031

Supplemental Table S1. Three up- and 18 down- regulated tear proteins in AITD vs normal controls (fold change > 1.5, or < 0.67, p value < 0.05).

Up regulated proteins		
Gene Symbol	Fold Change (geometric mean)	p-value
LACRT	4.605	0.031
LYZ	3.363	0.045
MSLN	3.285	0.028
AZGP1	3.089	0.046
CLU	2.415	0.030
**Down regulated proteins**		
**Gene Symbol**	**Fold Change (geometric mean)**	**p-value**
ADH1C	0.077	0.007
PRR4	0.098	0.027
FBP1	0.179	0.001
PPIA	0.284	0.021
AKR1C1	0.303	0.036
PFN2	0.305	0.022
PSMB9	0.306	0.046
LAP3	0.332	0.004
ENO1	0.336	0.045
CAPG	0.346	0.033
HSPA1B;HSPA1A	0.354	0.010
PRDX1	0.357	0.021
A2M	0.375	0.010
ANXA3	0.388	0.011
SELENBP1	0.390	0.013
APRT	0.397	0.026
TPI1;TPI1P1	0.399	0.011
CALML3	0.401	0.034
LDHA	0.404	0.033
NQO1	0.404	0.044
ALDH1A3	0.417	0.005
S100A6	0.432	0.007
PKM2	0.436	0.026
ALDOA	0.442	0.010
TKT	0.447	0.008
HSPB1	0.448	0.034
AKR1A1	0.450	0.043
CAP1	0.469	0.047
CCT8	0.469	0.031
UGP2	0.495	0.040
ANXA5	0.505	0.029

PRDX2	0.506	0.044
YWHAZ	0.508	0.003
PARK7	0.511	0.034
YWHAB	0.522	0.005
GSTO1	0.534	0.019
ORM1	0.544	0.043
VCL	0.548	0.014
GBE1	0.560	0.037
TYMP	0.563	0.045
SORD	0.575	0.007
HSPA8	0.593	0.032
PGK1	0.594	0.030
GSS	0.608	0.020
CMPK1	0.625	0.005
SFN	0.636	0.025

Supplemental Table S2. Five up- and 46 down- regulated tear proteins in mild TED vs normal controls (fold change > 1.5, or < 0.67, p value < 0.05).

Up regulated proteins		
**Gene Symbol**	**Fold Change (geometric mean)**	**p-value**
PRR4;PRH2;PRH1	13.996	0.000
LACRT	7.122	0.002
AZGP1	5.535	0.007
LYZ	4.891	0.029
IGHA1	4.813	0.017
CLU	4.622	0.002
MSLN	4.406	0.023
PIP	4.188	0.023
CST4	3.929	0.005
PIGR	3.316	0.041
CHI3L2	3.078	0.007
LGALS3BP	2.658	0.005
HSPG2	2.626	0.003
IGJ	2.251	0.049
PLTP	2.173	0.026
C4orf40	2.152	0.027
SCGB2A1	2.012	0.041
TCN1	2.010	0.048
IGKV2-30	1.994	0.032
CST3	1.965	0.038
IGLV3-19	1.895	0.049
GOLM1	1.831	0.036
IGKV3D-15	1.731	0.023
IGLV1-47	1.614	0.021

Down regulated proteins		
**Gene Symbol**	**Fold Change (geometric mean)**	**p-value**
ANXA5	0.189	0.005
IGHG2	0.210	0.035
ENO1	0.231	0.005
TPI1;TPI1P1	0.242	0.018
PGK1	0.251	0.002
AKR1C1	0.260	0.037
PRDX5	0.277	0.010
CAPG	0.284	0.004
ALDH1A1	0.287	0.015
PRDX1	0.288	0.003
HSPA1B;HSPA1A	0.291	0.006
PSMA1	0.319	0.028

ASS1	0.321	0.018
S100A6	0.327	0.001
S100A4	0.332	0.029
PRDX5	0.337	0.015
TAGLN2	0.352	0.006
LDHA	0.355	0.007
ADH1C	0.362	0.026
SELENBP1	0.370	0.014
ALDOA	0.380	0.012
PARK7	0.383	0.017
SORD	0.385	0.023
PSMB9	0.391	0.038
PEBP1	0.400	0.000
AGR2	0.411	0.014
LXN	0.424	0.011
LAP3	0.431	0.021
PPP1R7	0.434	0.044
ORM2	0.442	0.020
NAPRT1	0.453	0.003
YWHAZ	0.460	0.024
FGG	0.493	0.005
TALDO1	0.503	0.016
AKR1A1	0.504	0.000
HSPA8	0.518	0.024
HSP90AA1	0.532	0.023
YWHAB	0.534	0.020
PCBD1	0.540	0.025
FBP1	0.568	0.032
IDH1	0.576	0.019
SLC9A3R1	0.643	0.049
RAB1B	0.654	0.037
PYGL	0.662	0.023

Supplemental Table S3. Twenty-four up- and 44 down- regulated tear proteins in severe TED vs normal controls (fold change > 1.5, or < 0.67, p value < 0.05).

N	Accession #	Protein Name	Unused Score	Peptides (> 95% confidence)
1	P02788	Lactotransferrin GN=LTF PE=1 SV=6	383.29	624
2	P02768	Serum albumin GN=ALB PE=1 SV=2	276.39	494
3	P01024	Complement C3 GN=C3 PE=1 SV=2	181.52	132
4	P98160	Basement membrane-specific heparan sulfate proteoglycan core protein GN=HSPG2 PE=1 SV=4	149.27	80
5	P01876	Ig alpha-1 chain C region GN=IGHA1 PE=1 SV=2	148.04	222
6	P01833	Polymeric immunoglobulin receptor GN=PIGR PE=1 SV=4	134.68	200
7	P31025	Lipocalin-1 GN=LCN1 PE=1 SV=1	127.41	303
8	P01877	Ig alpha-2 chain C region GN=IGHA2 PE=1 SV=3	125.96	188
9	P61626	Lysozyme C GN=LYZ PE=1 SV=1	117.29	261
10	P02787	Serotransferrin GN=TF PE=1 SV=3	106.44	122
11	Q9UGM3	Deleted in malignant brain tumors 1 protein GN=DMBT1 PE=1 SV=2	93.12	88
12	P00352	Retinal dehydrogenase 1 GN=ALDH1A1 PE=1 SV=2	89.27	76
13	P25311	Zinc-alpha-2-glycoprotein GN=AZGP1 PE=1 SV=2	87.35	125
14	P63261	Actin, cytoplasmic 2 GN=ACTG1 PE=1 SV=1	78.99	106
15	P21980	Protein-glutamine gamma-glutamyltransferase 2 GN=TGM2 PE=1 SV=2	78.54	59
16	P00450	Ceruloplasmin GN=CP PE=1 SV=1	78.33	60
17	P01023	Alpha-2-macroglobulin GN=A2M PE=1 SV=3	77.53	43
18	O43707	Alpha-actinin-4 GN=ACTN4 PE=1 SV=2	76.54	50
19	P60709	Actin, cytoplasmic 1 GN=ACTB PE=1 SV=1	72.19	108

20	P14618	Pyruvate kinase PKM GN=PKM PE=1 SV=4	71.71	66
21	P08107	Heat shock 70 kDa protein 1A/1B GN=HSPA1A PE=1 SV=5	69.56	52
22	P06733	Alpha-enolase GN=ENO1 PE=1 SV=2	64.63	78
23	Q13228	Selenium-binding protein 1 GN=SELENBP1 PE=1 SV=2	60.9	43
24	P01857	Ig gamma-1 chain C region GN=IGHG1 PE=1 SV=1	60.73	69
25	P0C0L5	Complement C4-B GN=C4B PE=1 SV=2	60.51	33
26	Q16378	Proline-rich protein 4 GN=PRR4 PE=1 SV=3	59.72	83
27	P0C0L4	Complement C4-A GN=C4A PE=1 SV=2	59.16	33
28	P06396	Gelsolin GN=GSN PE=1 SV=1	56.86	57
29	P07355	Annexin A2 GN=ANXA2 PE=1 SV=2	54.92	42
30	Q9GZZ8	Extracellular glycoprotein lacritin GN=LACRT PE=1 SV=1	54.72	129
31	P04083	Annexin A1 GN=ANXA1 PE=1 SV=2	54.52	62
32	P22314	Ubiquitin-like modifier-activating enzyme 1 GN=UBA1 PE=1 SV=3	54.51	32
33	P07900	Heat shock protein HSP 90-alpha GN=HSP90AA1 PE=1 SV=5	54.42	30
34	P30740	Leukocyte elastase inhibitor GN=SERPINB1 PE=1 SV=1	53.84	58
35	Q13421	Mesothelin GN=MSLN PE=1 SV=2	52.42	42
36	P00738	Haptoglobin GN=HP PE=1 SV=1	52.12	45
37	P01009	Alpha-1-antitrypsin GN=SERPINA1 PE=1 SV=3	50.47	42
38	P01859	Ig gamma-2 chain C region GN=IGHG2 PE=1 SV=2	50.03	57
39	P50395	Rab GDP dissociation inhibitor beta GN=GDI2 PE=1 SV=2	48.43	28
40	P13639	Elongation factor 2 GN=EEF2 PE=1 SV=4	48.35	29

41	P00338	L-lactate dehydrogenase A chain GN=LDHA PE=1 SV=2	48.08	39
42	P68104	Elongation factor 1-alpha 1 GN=EEF1A1 PE=1 SV=1	47.19	37
43	O75556	Mammaglobin-B GN=SCGB2A1 PE=1 SV=1	46.5	99
44	P14550	Alcohol dehydrogenase [NADP(+)] GN=AKR1A1 PE=1 SV=3	45.81	32
45	P01834	Ig kappa chain C region GN=IGKC PE=1 SV=1	44.1	90
46	Q09666	Neuroblast differentiation-associated protein AHNAK GN=AHNAK PE=1 SV=2	43.9	25
47	P08603	Complement factor H GN=CFH PE=1 SV=4	43.43	24
48	P22079	Lactoperoxidase GN=LPO PE=1 SV=2	42.92	33
49	P06703	Protein S100-A6 GN=S100A6 PE=1 SV=1	42.77	58
50	P20061	Transcobalamin-1 GN=TCN1 PE=1 SV=2	42.12	32
51	Q99935	Proline-rich protein 1 GN=PROL1 PE=1 SV=2	41.95	48
52	P15311	Ezrin GN=EZR PE=1 SV=4	41.83	31
53	P00558	Phosphoglycerate kinase 1 GN=PGK1 PE=1 SV=3	41.5	38
54	Q7Z406	Myosin-14 GN=MYH14 PE=1 SV=2	41.25	22
55	P02790	Hemopexin GN=HPX PE=1 SV=2	41.17	32
56	P55072	Transitional endoplasmic reticulum ATPase GN=VCP PE=1 SV=4	41.12	24
57	P04406	Glyceraldehyde-3-phosphate dehydrogenase GN=GAPDH PE=1 SV=3	40.71	41
58	P12273	Prolactin-inducible protein GN=PIP PE=1 SV=1	39.88	78
59	Q96KP4	Cytosolic non-specific dipeptidase GN=CNDP2 PE=1 SV=2	39.87	27
60	Q06830	Peroxiredoxin-1 GN=PRDX1 PE=1 SV=1	39.75	42
61	P10909	Clusterin GN=CLU PE=1 SV=1	39.67	37

62	P07384	Calpain-1 catalytic subunit GN=CAPN1 PE=1 SV=1	39.44	24
63	P09211	Glutathione S-transferase P GN=GSTP1 PE=1 SV=2	39.28	56
64	Q04828	Aldo-keto reductase family 1 member C1 GN=AKR1C1 PE=1 SV=1	39.24	25
65	P68371	Tubulin beta-4B chain GN=TUBB4B PE=1 SV=1	38.91	27
66	P26447	Protein S100-A4 GN=S100A4 PE=1 SV=1	38.84	38
67	O95968	Secretoglobin family 1D member 1 GN=SCGB1D1 PE=1 SV=1	38.7	45
68	P08238	Heat shock protein HSP 90-beta GN=HSP90AB1 PE=1 SV=4	38.45	26
69	P01871	Ig mu chain C region GN=IGHM PE=1 SV=3	38.44	35
70	P04075	Fructose-bisphosphate aldolase A GN=ALDOA PE=1 SV=2	38.19	36
71	P52209	6-phosphogluconate dehydrogenase, decarboxylating GN=PGD PE=1 SV=3	37.95	26
72	P13645	Keratin, type I cytoskeletal 10 GN=KRT10 PE=1 SV=6	37.88	25
73	P06737	Glycogen phosphorylase, liver form GN=PYGL PE=1 SV=4	37.69	25
74	P08727	Keratin, type I cytoskeletal 19 GN=KRT19 PE=1 SV=4	37.63	26
75	P13797	Plastin-3 GN=PLS3 PE=1 SV=4	37.54	22
76	P28838	Cytosol aminopeptidase GN=LAP3 PE=1 SV=3	37.37	21
77	P30041	Peroxiredoxin-6 GN=PRDX6 PE=1 SV=3	37.36	32
78	P01037	Cystatin-SN GN=CST1 PE=1 SV=3	37.07	67
79	P07437	Tubulin beta chain GN=TUBB PE=1 SV=2	36.29	27
80	P00326	Alcohol dehydrogenase 1C GN=ADH1C PE=1 SV=2	35.84	21

81	P11142	Heat shock cognate 71 kDa protein GN=HSPA8 PE=1 SV=1	35.65	33
82	P02774	Vitamin D-binding protein GN=GC PE=1 SV=1	35.61	22
83	Q08380	Galectin-3-binding protein GN=LGALS3BP PE=1 SV=1	34.99	31
84	Q6XQN6	Nicotinate phosphoribosyltransferase GN=NAPRT PE=1 SV=2	34.84	20
85	P04792	Heat shock protein beta-1 GN=HSPB1 PE=1 SV=2	34.67	42
86	P05109	Protein S100-A8 GN=S100A8 PE=1 SV=1	34.37	48
87	P35579	Myosin-9 GN=MYH9 PE=1 SV=4	34.33	18
88	P13489	Ribonuclease inhibitor GN=RNH1 PE=1 SV=2	34.02	21
89	P46940	Ras GTPase-activating-like protein IQGAP1 GN=IQGAP1 PE=1 SV=1	34.01	18
90	P04264	Keratin, type II cytoskeletal 1 GN=KRT1 PE=1 SV=6	33.9	23
91	P01036	Cystatin-S GN=CST4 PE=1 SV=3	33.87	59
92	P00390	Glutathione reductase, mitochondrial GN=GSR PE=1 SV=2	33.66	22
93	P30838	Aldehyde dehydrogenase, dimeric NADP-preferring GN=ALDH3A1 PE=1 SV=3	33.24	32
94	P13647	Keratin, type II cytoskeletal 5 GN=KRT5 PE=1 SV=3	33.03	21
95	P98088	Mucin-5AC (Fragments) GN=MUC5AC PE=1 SV=3	32.81	18
96	P55786	Puromycin-sensitive aminopeptidase GN=NPEPPS PE=1 SV=2	32.14	17
97	Q06323	Proteasome activator complex subunit 1 GN=PSME1 PE=1 SV=1	32	19
98	P00751	Complement factor B GN=CFB PE=1 SV=2	31.49	19
99	P62258	14-3-3 protein epsilon GN=YWHAE PE=1 SV=1	31	29

100	P01008	Antithrombin-III GN=SERPINC1 PE=1 SV=1	30.74	20
101	P47895	Aldehyde dehydrogenase family 1 member A3 GN=ALDH1A3 PE=1 SV=2	30.59	19
102	P63104	14-3-3 protein zeta/delta GN=YWHAZ PE=1 SV=1	30.51	33
103	P60174	Triosephosphate isomerase GN=TPI1 PE=1 SV=3	30.46	33
104	P30044	Peroxiredoxin-5, mitochondrial GN=PRDX5 PE=1 SV=4	30.22	32
105	P07237	Protein disulfide-isomerase GN=P4HB PE=1 SV=3	30.08	18
106	P01011	Alpha-1-antichymotrypsin GN=SERPINA3 PE=1 SV=2	30.01	22
107	O75083	WD repeat-containing protein 1 GN=WDR1 PE=1 SV=4	29.55	17
108	P20810	Calpastatin GN=CAST PE=1 SV=4	29.44	17
109	Q9H4A4	Aminopeptidase B GN=RNPEP PE=1 SV=2	29.38	16
110	Q01518	Adenylyl cyclase-associated protein 1 GN=CAP1 PE=1 SV=5	29.32	19
111	P36952	Serpin B5 GN=SERPINB5 PE=1 SV=2	29.19	19
112	P01591	Immunoglobulin J chain GN=IGJ PE=1 SV=4	28.95	32
113	Q01469	Fatty acid-binding protein, epidermal GN=FABP5 PE=1 SV=3	28.2	23
114	P19971	Thymidine phosphorylase GN=TYMP PE=1 SV=2	28.19	20
115	P00966	Argininosuccinate synthase GN=ASS1 PE=1 SV=2	28.18	19
116	P0CG06	Ig lambda-3 chain C regions GN=IGLC3 PE=1 SV=1	28.18	64
117	B9A064	Immunoglobulin lambda-like polypeptide 5 GN=IGLL5 PE=2 SV=2	27.99	62
118	P61769	Beta-2-microglobulin GN=B2M PE=1 SV=1	27.59	24

119	P62937	Peptidyl-prolyl cis-trans isomerase A GN=PPIA PE=1 SV=2	26.65	31
120	P08758	Annexin A5 GN=ANXA5 PE=1 SV=2	26.5	20
121	P18206	Vinculin GN=VCL PE=1 SV=4	26.27	17
122	P31947	14-3-3 protein sigma GN=SFN PE=1 SV=1	25.99	25
123	P06702	Protein S100-A9 GN=S100A9 PE=1 SV=1	25.97	80
124	P09960	Leukotriene A-4 hydrolase GN=LTA4H PE=1 SV=2	25.92	14
125	O75874	Isocitrate dehydrogenase [NADP] cytoplasmic GN=IDH1 PE=1 SV=2	25.8	16
126	P68363	Tubulin alpha-1B chain GN=TUBA1B PE=1 SV=1	25.69	17
127	P19013	Keratin, type II cytoskeletal 4 GN=KRT4 PE=1 SV=4	25.55	16
128	P09467	Fructose-1,6-bisphosphatase 1 GN=FBP1 PE=1 SV=5	25.38	17
129	P16152	Carbonyl reductase [NADPH] 1 GN=CBR1 PE=1 SV=3	25.18	15
130	P18136	Ig kappa chain V-III region HIC PE=2 SV=2	25.01	31
131	P50995	Annexin A11 GN=ANXA11 PE=1 SV=1	24.86	13
132	P02647	Apolipoprotein A-I GN=APOA1 PE=1 SV=1	24.81	17
133	Q8WUM4	Programmed cell death 6-interacting protein GN=PDCD6IP PE=1 SV=1	24.61	14
134	P30101	Protein disulfide-isomerase A3 GN=PDIA3 PE=1 SV=4	24.56	13
135	P02749	Beta-2-glycoprotein 1 GN=APOH PE=1 SV=3	24.35	18
136	P04217	Alpha-1B-glycoprotein GN=A1BG PE=1 SV=4	24.29	15
137	P05787	Keratin, type II cytoskeletal 8 GN=KRT8 PE=1 SV=7	24.05	14
138	P02763	Alpha-1-acid glycoprotein 1 GN=ORM1 PE=1 SV=1	24	18

139	O60437	Periplakin GN=PPL PE=1 SV=4	23.88	13
140	Q96DA0	Zymogen granule protein 16 homolog B GN=ZG16B PE=1 SV=3	23.72	19
141	P17655	Calpain-2 catalytic subunit GN=CAPN2 PE=1 SV=6	23.69	14
142	P80303	Nucleobindin-2 GN=NUCB2 PE=1 SV=2	23.46	16
143	P29401	Transketolase GN=TKT PE=1 SV=3	23.45	15
144	P0CG05	Ig lambda-2 chain C regions GN=IGLC2 PE=1 SV=1	23.24	63
145	P61158	Actin-related protein 3 GN=ACTR3 PE=1 SV=3	23.19	14
146	P07858	Cathepsin B GN=CTSB PE=1 SV=3	23.1	17
147	O60664	Perilipin-3 GN=PLIN3 PE=1 SV=3	22.58	15
148	P02671	Fibrinogen alpha chain GN=FGA PE=1 SV=2	22.57	14
149	P40394	Alcohol dehydrogenase class 4 mu/sigma chain GN=ADH7 PE=1 SV=2	22.54	19
150	P36871	Phosphoglucomutase-1 GN=PGM1 PE=1 SV=3	22.49	13
151	O00299	Chloride intracellular channel protein 1 GN=CLIC1 PE=1 SV=4	22.32	16
152	P02766	Transthyretin GN=TTR PE=1 SV=1	22.18	17
153	P07602	Prosaposin GN=PSAP PE=1 SV=2	22.07	13
154	P43490	Nicotinamide phosphoribosyltransferase GN=NAMPT PE=1 SV=1	21.96	12
155	P07203	Glutathione peroxidase 1 GN=GPX1 PE=1 SV=4	21.91	12
156	P06744	Glucose-6-phosphate isomerase GN=GPI PE=1 SV=4	21.9	19
157	Q14624	Inter-alpha-trypsin inhibitor heavy chain H4 GN=ITIH4 PE=1 SV=4	21.71	12
158	P17931	Galectin-3 GN=LGALS3 PE=1 SV=5	21.67	27

159	P07339	Cathepsin D GN=CTSD PE=1 SV=1	21.49	14
160	Q9NY33	Dipeptidyl peptidase 3 GN=DPP3 PE=1 SV=2	20.82	13
161	P31939	Bifunctional purine biosynthesis protein PURH GN=ATIC PE=1 SV=3	20.8	11
162	Q86VP6	Cullin-associated NEDD8-dissociated protein 1 GN=CAND1 PE=1 SV=2	20.77	12
163	P40121	Macrophage-capping protein GN=CAPG PE=1 SV=2	20.71	15
164	P23528	Cofilin-1 GN=CFL1 PE=1 SV=3	20.69	15
165	P18669	Phosphoglycerate mutase 1 GN=PGAM1 PE=1 SV=2	20.66	18
166	P48637	Glutathione synthetase GN=GSS PE=1 SV=1	20.46	12
167	P37802	Transgelin-2 GN=TAGLN2 PE=1 SV=3	20.39	23
168	O00151	PDZ and LIM domain protein 1 GN=PDLIM1 PE=1 SV=4	20.37	14
169	O95834	Echinoderm microtubule-associated protein-like 2 GN=EML2 PE=1 SV=1	20.35	10
170	O95336	6-phosphogluconolactonase GN=PGLS PE=1 SV=2	20.15	13
171	P22392	Nucleoside diphosphate kinase B GN=NME2 PE=1 SV=1	20.14	14
172	P40925	Malate dehydrogenase, cytoplasmic GN=MDH1 PE=1 SV=4	20.03	17
173	O00391	Sulfhydryl oxidase 1 GN=QSOX1 PE=1 SV=3	20.01	10
174	Q99497	Protein DJ-1 GN=PARK7 PE=1 SV=2	20	16
175	O75891	Cytosolic 10-formyltetrahydrofolate dehydrogenase GN=ALDH1L1 PE=1 SV=2	20	13
176	P13646	Keratin, type I cytoskeletal 13 GN=KRT13 PE=1 SV=4	19.75	22
177	P01042	Kininogen-1 GN=KNG1 PE=1 SV=2	19.73	12
178	P00747	Plasminogen GN=PLG PE=1 SV=2	19.73	11

179	P05155	Plasma protease C1 inhibitor GN=SERPING1 PE=1 SV=2	19.71	15
180	P80188	Neutrophil gelatinase-associated lipocalin GN=LCN2 PE=1 SV=2	19.69	23
181	Q9Y6U3	Adseverin GN=SCIN PE=1 SV=4	19.68	11
182	Q02818	Nucleobindin-1 GN=NUCB1 PE=1 SV=4	19.59	11
183	Q8NBJ4	Golgi membrane protein 1 GN=GOLM1 PE=1 SV=1	19.56	11
184	Q16851	UTP--glucose-1-phosphate uridylyltransferase GN=UGP2 PE=1 SV=5	19.44	10
185	Q15181	Inorganic pyrophosphatase GN=PPA1 PE=1 SV=2	19.43	12
186	P30085	UMP-CMP kinase GN=CMPK1 PE=1 SV=3	19.4	14
187	Q9HC38	Glyoxalase domain-containing protein 4 GN=GLOD4 PE=1 SV=1	19.07	11
188	P52565	Rho GDP-dissociation inhibitor 1 GN=ARHGDIA PE=1 SV=3	18.98	15
189	P12429	Annexin A3 GN=ANXA3 PE=1 SV=3	18.89	11
190	P34932	Heat shock 70 kDa protein 4 GN=HSPA4 PE=1 SV=4	18.8	10
191	P07195	L-lactate dehydrogenase B chain GN=LDHB PE=1 SV=2	18.71	15
192	P04632	Calpain small subunit 1 GN=CAPNS1 PE=1 SV=1	18.63	15
193	Q71U36	Tubulin alpha-1A chain GN=TUBA1A PE=1 SV=1	18.54	15
194	P36955	Pigment epithelium-derived factor GN=SERPINF1 PE=1 SV=4	18.49	11
195	P11021	78 kDa glucose-regulated protein GN=HSPA5 PE=1 SV=2	18.39	15
196	P09525	Annexin A4 GN=ANXA4 PE=1 SV=4	18.32	12
197	P02814	Submaxillary gland androgen-regulated protein 3B GN=SMR3B PE=1 SV=2	18.08	26

198	P30086	Phosphatidylethanolamine-binding protein 1 GN=PEBP1 PE=1 SV=3	18.06	16
199	P31946	14-3-3 protein beta/alpha GN=YWHAB PE=1 SV=3	18	22
200	P00734	Prothrombin GN=F2 PE=1 SV=2	18	9
201	P18510	Interleukin-1 receptor antagonist protein GN=IL1RN PE=1 SV=1	17.92	14
202	P21281	V-type proton ATPase subunit B, brain isoform GN=ATP6V1B2 PE=1 SV=3	17.84	10
203	P63241	Eukaryotic translation initiation factor 5A-1 GN=EIF5A PE=1 SV=2	17.8	11
204	P01601	Ig kappa chain V-I region HK101 (Fragment) PE=4 SV=1	17.75	17
205	P02765	Alpha-2-HS-glycoprotein GN=AHSG PE=1 SV=1	17.56	16
206	P15104	Glutamine synthetase GN=GLUL PE=1 SV=4	17.39	10
207	P01034	Cystatin-C GN=CST3 PE=1 SV=1	17.21	14
208	Q13938	Calcyphosin GN=CAPS PE=1 SV=1	17.19	9
209	P45974	Ubiquitin carboxyl-terminal hydrolase 5 GN=USP5 PE=1 SV=2	17.16	9
210	P62158	Calmodulin GN=CALM1 PE=1 SV=2	17.15	14
211	P02679	Fibrinogen gamma chain GN=FGG PE=1 SV=3	17.15	10
212	P60981	Destrin GN=DSTN PE=1 SV=3	16.85	10
213	P01764	Ig heavy chain V-III region VH26 PE=1 SV=1	16.71	25
214	Q96C19	EF-hand domain-containing protein D2 GN=EFHD2 PE=1 SV=1	16.65	9
215	P61160	Actin-related protein 2 GN=ACTR2 PE=1 SV=1	16.51	9
216	P18135	Ig kappa chain V-III region HAH PE=2 SV=1	16.47	23
217	P02675	Fibrinogen beta chain GN=FGB PE=1 SV=2	16.43	9

218	Q15782	Chitinase-3-like protein 2 GN=CHI3L2 PE=1 SV=1	16.39	10
219	P08729	Keratin, type II cytoskeletal 7 GN=KRT7 PE=1 SV=5	16.33	13
220	P04080	Cystatin-B GN=CSTB PE=1 SV=2	16.09	17
221	P23526	Adenosylhomocysteinase GN=AHCY PE=1 SV=4	16.05	10
222	P67936	Tropomyosin alpha-4 chain GN=TPM4 PE=1 SV=3	16.01	11
223	P50990	T-complex protein 1 subunit theta GN=CCT8 PE=1 SV=4	16.01	8
224	Q00796	Sorbitol dehydrogenase GN=SORD PE=1 SV=4	16	10
225	P78371	T-complex protein 1 subunit beta GN=CCT2 PE=1 SV=4	16	8
226	P01766	Ig heavy chain V-III region BRO PE=1 SV=1	16	35
227	P01019	Angiotensinogen GN=AGT PE=1 SV=1	15.77	8
228	P31949	Protein S100-A11 GN=S100A11 PE=1 SV=2	15.6	33
229	P32119	Peroxiredoxin-2 GN=PRDX2 PE=1 SV=5	15.58	22
230	P55058	Phospholipid transfer protein GN=PLTP PE=1 SV=1	15.52	11
231	P27797	Calreticulin GN=CALR PE=1 SV=1	15.35	9
232	P37837	Transaldolase GN=TALDO1 PE=1 SV=2	15.34	8
233	P17987	T-complex protein 1 subunit alpha GN=TCP1 PE=1 SV=1	15.13	8
234	O75369	Filamin-B GN=FLNB PE=1 SV=2	15.12	8
235	Q9UL46	Proteasome activator complex subunit 2 GN=PSME2 PE=1 SV=4	15.02	11
236	P21964	Catechol O-methyltransferase GN=COMT PE=1 SV=2	14.97	11
237	P17858	ATP-dependent 6-phosphofructokinase, liver type GN=PFKL PE=1 SV=6	14.97	11

238	P30153	Serine/threonine-protein phosphatase 2A 65 kDa regulatory subunit A alpha isoform GN=PPP2R1A PE=1 SV=4	14.94	9
239	Q06210	Glutamine--fructose-6-phosphate aminotransferase [isomerizing] 1 GN=GFPT1 PE=1 SV=3	14.86	8
240	P08670	Vimentin GN=VIM PE=1 SV=4	14.74	9
241	P47756	F-actin-capping protein subunit beta GN=CAPZB PE=1 SV=4	14.74	11
242	Q16719	Kynureninase GN=KYNU PE=1 SV=1	14.57	9
243	P35527	Keratin, type I cytoskeletal 9 GN=KRT9 PE=1 SV=3	14.53	8
244	P60660	Myosin light polypeptide 6 GN=MYL6 PE=1 SV=2	14.49	13
245	O00764	Pyridoxal kinase GN=PDXK PE=1 SV=1	14.48	9
246	P47755	F-actin-capping protein subunit alpha-2 GN=CAPZA2 PE=1 SV=3	14.48	8
247	P19827	Inter-alpha-trypsin inhibitor heavy chain H1 GN=ITIH1 PE=1 SV=3	14.33	9
248	P14314	Glucosidase 2 subunit beta GN=PRKCSH PE=1 SV=2	14.22	9
249	P02750	Leucine-rich alpha-2-glycoprotein GN=LRG1 PE=1 SV=2	14.18	9
250	P52907	F-actin-capping protein subunit alpha-1 GN=CAPZA1 PE=1 SV=3	14.16	9
251	P13798	Acylamino-acid-releasing enzyme GN=APEH PE=1 SV=4	14.14	8
252	Q96G03	Phosphoglucomutase-2 GN=PGM2 PE=1 SV=4	14.12	9
253	P11413	Glucose-6-phosphate 1-dehydrogenase GN=G6PD PE=1 SV=4	14.08	8
254	P60842	Eukaryotic initiation factor 4A-I GN=EIF4A1 PE=1 SV=1	14.03	9
255	P10599	Thioredoxin GN=TXN PE=1 SV=3	14.01	11

256	P07737	Profilin-1 GN=PFN1 PE=1 SV=2	14	18
257	O95994	Anterior gradient protein 2 homolog GN=AGR2 PE=1 SV=1	14	14
258	Q14914	Prostaglandin reductase 1 GN=PTGR1 PE=1 SV=2	14	8
259	P52566	Rho GDP-dissociation inhibitor 2 GN=ARHGDIB PE=1 SV=3	14	9
260	P04196	Histidine-rich glycoprotein GN=HRG PE=1 SV=1	14	9
261	P02748	Complement component C9 GN=C9 PE=1 SV=2	14	7
262	O60547	GDP-mannose 4,6 dehydratase GN=GMDS PE=1 SV=1	13.8	8
263	O00534	von Willebrand factor A domain-containing protein 5A GN=VWA5A PE=2 SV=2	13.79	8
264	Q13813	Spectrin alpha chain, non-erythrocytic 1 GN=SPTAN1 PE=1 SV=3	13.77	10
265	P20618	Proteasome subunit beta type-1 GN=PSMB1 PE=1 SV=2	13.73	9
266	P31941	DNA dC->dU-editing enzyme APOBEC-3A GN=APOBEC3A PE=1 SV=3	13.7	10
267	P13796	Plastin-2 GN=LCP1 PE=1 SV=6	13.69	13
268	Q9ULZ3	Apoptosis-associated speck-like protein containing a CARD GN=PYCARD PE=1 SV=2	13.64	7
269	P61978	Heterogeneous nuclear ribonucleoprotein K GN=HNRNPK PE=1 SV=1	13.48	7
270	Q14651	Plastin-1 GN=PLS1 PE=1 SV=2	13.45	12
271	P05388	60S acidic ribosomal protein P0 GN=RPLP0 PE=1 SV=1	13.41	7
272	P30046	D-dopachrome decarboxylase GN=DDT PE=1 SV=3	13.38	8
273	P01768	Ig heavy chain V-III region CAM PE=1 SV=1	13.33	15
274	P46459	Vesicle-fusing ATPase GN=NSF PE=1 SV=3	13.29	8

275	Q14515	SPARC-like protein 1 GN=SPARCL1 PE=1 SV=2	13.25	7
276	O15144	Actin-related protein 2/3 complex subunit 2 GN=ARPC2 PE=1 SV=1	13.23	7
277	Q9BRF8	Serine/threonine-protein phosphatase CPPED1 GN=CPPED1 PE=1 SV=3	13.21	9
278	P40926	Malate dehydrogenase, mitochondrial GN=MDH2 PE=1 SV=3	13.2	7
279	P01617	Ig kappa chain V-II region TEW PE=1 SV=1	13.1	19
280	P01610	Ig kappa chain V-I region WEA PE=1 SV=1	13.07	19
281	P04004	Vitronectin GN=VTN PE=1 SV=1	13.02	8
282	P31151	Protein S100-A7 GN=S100A7 PE=1 SV=4	13.01	10
283	P40227	T-complex protein 1 subunit zeta GN=CCT6A PE=1 SV=3	12.97	7
284	P07741	Adenine phosphoribosyltransferase GN=APRT PE=1 SV=2	12.93	9
285	P47989	Xanthine dehydrogenase/oxidase GN=XDH PE=1 SV=4	12.88	7
286	P06309	Ig kappa chain V-II region GM607 (Fragment) PE=4 SV=1	12.87	22
287	Q14247	Src substrate cortactin GN=CTTN PE=1 SV=2	12.85	7
288	P02511	Alpha-crystallin B chain GN=CRYAB PE=1 SV=2	12.81	8
289	O14818	Proteasome subunit alpha type-7 GN=PSMA7 PE=1 SV=1	12.71	7
290	Q14894	Ketimine reductase mu-crystallin GN=CRYM PE=1 SV=1	12.65	7
291	P06576	ATP synthase subunit beta, mitochondrial GN=ATP5B PE=1 SV=3	12.46	6
292	Q15435	Protein phosphatase 1 regulatory subunit 7 GN=PPP1R7 PE=1 SV=1	12.31	7
293	P01781	Ig heavy chain V-III region GAL PE=1 SV=1	12.16	14

294	Q9NQW7	Xaa-Pro aminopeptidase 1 GN=XPNPEP1 PE=1 SV=3	12.06	6
295	Q12792	Twinfilin-1 GN=TWF1 PE=1 SV=3	12	6
296	Q96FW1	Ubiquitin thioesterase OTUB1 GN=OTUB1 PE=1 SV=2	12	6
297	P35754	Glutaredoxin-1 GN=GLRX PE=1 SV=2	12	9
298	P27482	Calmodulin-like protein 3 GN=CALML3 PE=1 SV=2	12	7
299	P09651	Heterogeneous nuclear ribonucleoprotein A1 GN=HNRNPA1 PE=1 SV=5	12	7
300	O75368	SH3 domain-binding glutamic acid-rich-like protein GN=SH3BGRL PE=1 SV=1	11.92	6
301	P38606	V-type proton ATPase catalytic subunit A GN=ATP6V1A PE=1 SV=2	11.88	6
302	P11216	Glycogen phosphorylase, brain form GN=PYGB PE=1 SV=5	11.86	9
303	P22626	Heterogeneous nuclear ribonucleoproteins A2/B1 GN=HNRNPA2B1 PE=1 SV=2	11.85	7
304	P49189	4-trimethylaminobutyraldehyde dehydrogenase GN=ALDH9A1 PE=1 SV=3	11.84	8
305	P12955	Xaa-Pro dipeptidase GN=PEPD PE=1 SV=3	11.8	7
306	P62140	Serine/threonine-protein phosphatase PP1-beta catalytic subunit GN=PPP1CB PE=1 SV=3	11.77	7
307	Q96C23	Aldose 1-epimerase GN=GALM PE=1 SV=1	11.72	6
308	Q14697	Neutral alpha-glucosidase AB GN=GANAB PE=1 SV=3	11.68	8
309	Q14240	Eukaryotic initiation factor 4A-II GN=EIF4A2 PE=1 SV=2	11.62	8
310	P46926	Glucosamine-6-phosphate isomerase 1 GN=GNPDA1 PE=1 SV=1	11.52	6
311	Q15293	Reticulocalbin-1 GN=RCN1 PE=1 SV=1	11.4	7
312	P05164	Myeloperoxidase GN=MPO PE=1 SV=1	11.38	7

313	P31948	Stress-induced-phosphoprotein 1 GN=STIP1 PE=1 SV=1	11.19	7
314	P61586	Transforming protein RhoA GN=RHOA PE=1 SV=1	11.15	7
315	P50991	T-complex protein 1 subunit delta GN=CCT4 PE=1 SV=4	11.12	7
316	P68402	Platelet-activating factor acetylhydrolase IB subunit beta GN=PAFAH1B2 PE=1 SV=1	11.04	7
317	Q14974	Importin subunit beta-1 GN=KPNB1 PE=1 SV=2	10.99	6
318	P27348	14-3-3 protein theta GN=YWHAQ PE=1 SV=1	10.97	15
319	P04179	Superoxide dismutase [Mn], mitochondrial GN=SOD2 PE=1 SV=2	10.96	8
320	P19105	Myosin regulatory light chain 12A GN=MYL12A PE=1 SV=2	10.92	7
321	P31150	Rab GDP dissociation inhibitor alpha GN=GDI1 PE=1 SV=2	10.82	19
322	P34896	Serine hydroxymethyltransferase, cytosolic GN=SHMT1 PE=1 SV=1	10.73	6
323	P49902	Cytosolic purine 5'-nucleotidase GN=NT5C2 PE=1 SV=1	10.72	7
324	P17174	Aspartate aminotransferase, cytoplasmic GN=GOT1 PE=1 SV=3	10.68	6
325	P48147	Prolyl endopeptidase GN=PREP PE=1 SV=2	10.65	7
326	P00441	Superoxide dismutase [Cu-Zn] GN=SOD1 PE=1 SV=2	10.55	7
327	Q8N474	Secreted frizzled-related protein 1 GN=SFRP1 PE=1 SV=1	10.55	8
328	O14745	Na(+)/H(+) exchange regulatory cofactor NHE-RF1 GN=SLC9A3R1 PE=1 SV=4	10.54	6
329	P05546	Heparin cofactor 2 GN=SERPIND1 PE=1 SV=3	10.46	7
330	Q9UBC9	Small proline-rich protein 3 GN=SPRR3 PE=1 SV=2	10.45	6

331	P23381	Tryptophan--tRNA ligase, cytoplasmic GN=WARS PE=1 SV=2	10.37	7
332	P25788	Proteasome subunit alpha type-3 GN=PSMA3 PE=1 SV=2	10.31	6
333	O43852	Calumenin GN=CALU PE=1 SV=2	10.26	6
334	O15143	Actin-related protein 2/3 complex subunit 1B GN=ARPC1B PE=1 SV=3	10.21	6
335	P56537	Eukaryotic translation initiation factor 6 GN=EIF6 PE=1 SV=1	10.21	8
336	P25815	Protein S100-P GN=S100P PE=1 SV=2	10.14	11
337	Q9UNZ2	NSFL1 cofactor p47 GN=NSFL1C PE=1 SV=2	10.11	7
338	P62136	Serine/threonine-protein phosphatase PP1-alpha catalytic subunit GN=PPP1CA PE=1 SV=1	10.11	7
339	Q15365	Poly(rC)-binding protein 1 GN=PCBP1 PE=1 SV=2	10.09	7
340	Q96S96	Phosphatidylethanolamine-binding protein 4 GN=PEBP4 PE=1 SV=3	10.08	6
341	P19961	Alpha-amylase 2B GN=AMY2B PE=1 SV=1	10.04	6
342	Q02809	Procollagen-lysine,2-oxoglutarate 5-dioxygenase 1 GN=PLOD1 PE=1 SV=2	10.04	5
343	P35606	Coatomer subunit beta' GN=COPB2 PE=1 SV=2	10.03	5
344	Q9UN36	Protein NDRG2 GN=NDRG2 PE=1 SV=2	10.02	6
345	Q04917	14-3-3 protein eta GN=YWHAH PE=1 SV=4	10	13
346	Q9Y5Z4	Heme-binding protein 2 GN=HEBP2 PE=1 SV=1	10	8
347	P05387	60S acidic ribosomal protein P2 GN=RPLP2 PE=1 SV=1	10	5
348	P01700	Ig lambda chain V-I region HA PE=1 SV=1	10	7
349	Q13126	S-methyl-5'-thioadenosine phosphorylase GN=MTAP PE=1 SV=2	10	6

350	P01033	Metalloproteinase inhibitor 1 GN=TIMP1 PE=1 SV=1	10	7
351	P60953	Cell division control protein 42 homolog GN=CDC42 PE=1 SV=2	10	6
352	Q9BR76	Coronin-1B GN=CORO1B PE=1 SV=1	10	8
353	P01772	Ig heavy chain V-III region KOL PE=1 SV=1	10	14
354	Q9H0W9	Ester hydrolase C11orf54 GN=C11orf54 PE=1 SV=1	10	5
355	P60900	Proteasome subunit alpha type-6 GN=PSMA6 PE=1 SV=1	10	5
356	P11766	Alcohol dehydrogenase class-3 GN=ADH5 PE=1 SV=4	10	6
357	P28062	Proteasome subunit beta type-8 GN=PSMB8 PE=1 SV=3	10	5
358	P49773	Histidine triad nucleotide-binding protein 1 GN=HINT1 PE=1 SV=2	10	5
359	P01594	Ig kappa chain V-I region AU PE=1 SV=1	10	16
360	P01777	Ig heavy chain V-III region TEI PE=1 SV=1	10	31
361	P08134	Rho-related GTP-binding protein RhoC GN=RHOC PE=1 SV=1	10	6
362	P30043	Flavin reductase (NADPH) GN=BLVRB PE=1 SV=3	9.96	7
363	Q9Y678	Coatomer subunit gamma-1 GN=COPG1 PE=1 SV=1	9.92	5
364	P17812	CTP synthase 1 GN=CTPS1 PE=1 SV=2	9.92	6
365	P19823	Inter-alpha-trypsin inhibitor heavy chain H2 GN=ITIH2 PE=1 SV=2	9.91	6
366	P04745	Alpha-amylase 1 GN=AMY1A PE=1 SV=2	9.89	6
367	P19652	Alpha-1-acid glycoprotein 2 GN=ORM2 PE=1 SV=2	9.86	13
368	P35908	Keratin, type II cytoskeletal 2 epidermal GN=KRT2 PE=1 SV=2	9.82	11

369	P30520	Adenylosuccinate synthetase isozyme 2 GN=ADSS PE=1 SV=3	9.8	6
370	P43652	Afamin GN=AFM PE=1 SV=1	9.69	6
371	Q14376	UDP-glucose 4-epimerase GN=GALE PE=1 SV=2	9.68	6
372	Q92743	Serine protease HTRA1 GN=HTRA1 PE=1 SV=1	9.64	5
373	P49721	Proteasome subunit beta type-2 GN=PSMB2 PE=1 SV=1	9.64	5
374	P49720	Proteasome subunit beta type-3 GN=PSMB3 PE=1 SV=2	9.46	6
375	P51149	Ras-related protein Rab-7a GN=RAB7A PE=1 SV=1	9.41	5
376	Q96TA1	Niban-like protein 1 GN=FAM129B PE=1 SV=3	9.37	8
377	P43034	Platelet-activating factor acetylhydrolase IB subunit alpha GN=PAFAH1B1 PE=1 SV=2	9.36	6
378	P05090	Apolipoprotein D GN=APOD PE=1 SV=1	9.35	6
379	Q04760	Lactoylglutathione lyase GN=GLO1 PE=1 SV=4	9.34	7
380	P13010	X-ray repair cross-complementing protein 5 GN=XRCC5 PE=1 SV=3	9.3	5
381	P28325	Cystatin-D GN=CST5 PE=1 SV=1	9.29	6
382	P49368	T-complex protein 1 subunit gamma GN=CCT3 PE=1 SV=4	9.29	6
383	P04746	Pancreatic alpha-amylase GN=AMY2A PE=1 SV=2	9.29	5
384	P21399	Cytoplasmic aconitate hydratase GN=ACO1 PE=1 SV=3	9.27	6
385	P28066	Proteasome subunit alpha type-5 GN=PSMA5 PE=1 SV=3	9.2	5
386	Q96HC4	PDZ and LIM domain protein 5 GN=PDLIM5 PE=1 SV=5	9.2	6

387	Q99933	BAG family molecular chaperone regulator 1 GN=BAG1 PE=1 SV=4	9.19	5
388	P25787	Proteasome subunit alpha type-2 GN=PSMA2 PE=1 SV=2	9.11	6
389	P25789	Proteasome subunit alpha type-4 GN=PSMA4 PE=1 SV=1	9.11	5
390	O43776	Asparagine--tRNA ligase, cytoplasmic GN=NARS PE=1 SV=1	8.94	5
391	O43396	Thioredoxin-like protein 1 GN=TXNL1 PE=1 SV=3	8.9	5
392	P53004	Biliverdin reductase A GN=BLVRA PE=1 SV=2	8.84	5
393	Q9P2T1	GMP reductase 2 GN=GMPR2 PE=1 SV=1	8.75	5
394	P02760	Protein AMBP GN=AMBP PE=1 SV=1	8.66	6
395	Q9H0U4	Ras-related protein Rab-1B GN=RAB1B PE=1 SV=1	8.62	6
396	P49788	Retinoic acid receptor responder protein 1 GN=RARRES1 PE=1 SV=2	8.55	5
397	Q15366	Poly(rC)-binding protein 2 GN=PCBP2 PE=1 SV=1	8.5	5
398	P12956	X-ray repair cross-complementing protein 6 GN=XRCC6 PE=1 SV=2	8.49	5
399	P62820	Ras-related protein Rab-1A GN=RAB1A PE=1 SV=3	8.47	5
400	P06727	Apolipoprotein A-IV GN=APOA4 PE=1 SV=3	8.46	5
401	P78417	Glutathione S-transferase omega-1 GN=GSTO1 PE=1 SV=2	8.31	6
402	P28799	Granulins GN=GRN PE=1 SV=2	8.31	5
403	P23284	Peptidyl-prolyl cis-trans isomerase B GN=PPIB PE=1 SV=2	8.3	6
404	Q53FA7	Quinone oxidoreductase PIG3 GN=TP53I3 PE=1 SV=2	8.26	5
405	P35237	Serpin B6 GN=SERPINB6 PE=1 SV=3	8.25	7

406	P14174	Macrophage migration inhibitory factor GN=MIF PE=1 SV=4	8.21	9
407	Q01105	Protein SET GN=SET PE=1 SV=3	8.21	6
408	Q9UJ70	N-acetyl-D-glucosamine kinase GN=NAGK PE=1 SV=4	8.18	4
409	Q13217	DnaJ homolog subfamily C member 3 GN=DNAJC3 PE=1 SV=1	8.17	5
410	P23083	Ig heavy chain V-I region V35 PE=1 SV=1	8.16	9
411	P07108	Acyl-CoA-binding protein GN=DBI PE=1 SV=2	8.14	7
412	P54727	UV excision repair protein RAD23 homolog B GN=RAD23B PE=1 SV=1	8.14	6
413	P02652	Apolipoprotein A-II GN=APOA2 PE=1 SV=1	8.09	6
414	P20073	Annexin A7 GN=ANXA7 PE=1 SV=3	8.09	5
415	P22352	Glutathione peroxidase 3 GN=GPX3 PE=1 SV=2	8.09	5
416	P48643	T-complex protein 1 subunit epsilon GN=CCT5 PE=1 SV=1	8.07	5
417	P08263	Glutathione S-transferase A1 GN=GSTA1 PE=1 SV=3	8.07	5
418	Q32P51	Heterogeneous nuclear ribonucleoprotein A1-like 2 GN=HNRNPA1L2 PE=2 SV=2	8.07	4
419	O43488	Aflatoxin B1 aldehyde reductase member 2 GN=AKR7A2 PE=1 SV=3	8.06	5
420	O75223	Gamma-glutamylcyclotransferase GN=GGCT PE=1 SV=1	8.03	6
421	P50502	Hsc70-interacting protein GN=ST13 PE=1 SV=2	8.02	4
422	Q96IU4	Alpha/beta hydrolase domain-containing protein 14B GN=ABHD14B PE=1 SV=1	8.01	4
423	O95171	Sciellin GN=SCEL PE=1 SV=2	8.01	4
424	P55263	Adenosine kinase GN=ADK PE=1 SV=2	8.01	5
425	Q6ZVX7	F-box only protein 50 GN=NCCRP1 PE=1	8	6

		SV=1		
426	P62987	Ubiquitin-60S ribosomal protein L40 GN=UBA52 PE=1 SV=2	8	6
427	P33241	Lymphocyte-specific protein 1 GN=LSP1 PE=1 SV=1	8	4
428	P55957	BH3-interacting domain death agonist GN=BID PE=1 SV=1	8	4
429	Q9NZT1	Calmodulin-like protein 5 GN=CALML5 PE=1 SV=2	8	5
430	P62328	Thymosin beta-4 GN=TMSB4X PE=1 SV=2	8	11
431	P29692	Elongation factor 1-delta GN=EEF1D PE=1 SV=5	8	4
432	Q9UJU6	Drebrin-like protein GN=DBNL PE=1 SV=1	8	4
433	P63151	Serine/threonine-protein phosphatase 2A 55 kDa regulatory subunit B alpha isoform GN=PPP2R2A PE=1 SV=1	8	4
434	P09429	High mobility group protein B1 GN=HMGB1 PE=1 SV=3	8	4
435	O95436	Sodium-dependent phosphate transport protein 2B GN=SLC34A2 PE=1 SV=3	8	4
436	P36405	ADP-ribosylation factor-like protein 3 GN=ARL3 PE=1 SV=2	8	4
437	Q9BRA2	Thioredoxin domain-containing protein 17 GN=TXNDC17 PE=1 SV=1	8	4
438	Q9UHY7	Enolase-phosphatase E1 GN=ENOPH1 PE=1 SV=1	8	4
439	P80748	Ig lambda chain V-III region LOI PE=1 SV=1	8	8
440	P01743	Ig heavy chain V-I region HG3 PE=4 SV=1	8	8
441	P13716	Delta-aminolevulinic acid dehydratase GN=ALAD PE=1 SV=1	8	4
442	Q08623	Pseudouridine-5'-monophosphatase GN=HDHD1 PE=1 SV=3	8	4
443	P84077	ADP-ribosylation factor 1 GN=ARF1 PE=1	8	5

		SV=2		
444	P67775	Serine/threonine-protein phosphatase 2A catalytic subunit alpha isoform GN=PPP2CA PE=1 SV=1	8	4
445	P05452	Tetranectin GN=CLEC3B PE=1 SV=3	8	4
446	P04208	Ig lambda chain V-I region WAH PE=1 SV=1	8	6
447	Q15084	Protein disulfide-isomerase A6 GN=PDIA6 PE=1 SV=1	7.93	5
448	Q14508	WAP four-disulfide core domain protein 2 GN=WFDC2 PE=1 SV=2	7.92	5
449	O43865	Putative adenosylhomocysteinase 2 GN=AHCYL1 PE=1 SV=2	7.91	4
450	P46108	Adapter molecule crk GN=CRK PE=1 SV=2	7.89	4
451	P29373	Cellular retinoic acid-binding protein 2 GN=CRABP2 PE=1 SV=2	7.77	4
452	P01031	Complement C5 GN=C5 PE=1 SV=4	7.76	4
453	Q16881	Thioredoxin reductase 1, cytoplasmic GN=TXNRD1 PE=1 SV=3	7.75	4
454	P61088	Ubiquitin-conjugating enzyme E2 N GN=UBE2N PE=1 SV=1	7.72	6
455	Q13630	GDP-L-fucose synthase GN=TSTA3 PE=1 SV=1	7.7	5
456	Q9UBQ7	Glyoxylate reductase/hydroxypyruvate reductase GN=GRHPR PE=1 SV=1	7.69	6
457	Q8IZ83	Aldehyde dehydrogenase family 16 member A1 GN=ALDH16A1 PE=1 SV=2	7.66	5
458	O43175	D-3-phosphoglycerate dehydrogenase GN=PHGDH PE=1 SV=4	7.64	4
459	Q9NQR4	Omega-amidase NIT2 GN=NIT2 PE=1 SV=1	7.62	5
460	Q10567	AP-1 complex subunit beta-1 GN=AP1B1 PE=1 SV=2	7.62	4
461	O75351	Vacuolar protein sorting-associated protein 4B GN=VPS4B PE=1 SV=2	7.52	4

462	P63244	Guanine nucleotide-binding protein subunit beta-2-like 1 GN=GNB2L1 PE=1 SV=3	7.5	4
463	Q9NTK5	Obg-like ATPase 1 GN=OLA1 PE=1 SV=2	7.47	4
464	P08865	40S ribosomal protein SA GN=RPSA PE=1 SV=4	7.47	4
465	P14625	Endoplasmin GN=HSP90B1 PE=1 SV=1	7.45	4
466	Q01813	ATP-dependent 6-phosphofructokinase, platelet type GN=PFKP PE=1 SV=2	7.4	5
467	Q9UEW8	STE20/SPS1-related proline-alanine-rich protein kinase GN=STK39 PE=1 SV=3	7.39	4
468	P58107	Epiplakin GN=EPPK1 PE=1 SV=2	7.34	5
469	O00584	Ribonuclease T2 GN=RNASET2 PE=1 SV=2	7.33	4
470	P02538	Keratin, type II cytoskeletal 6A GN=KRT6A PE=1 SV=3	7.32	14
471	P08571	Monocyte differentiation antigen CD14 GN=CD14 PE=1 SV=2	7.32	5
472	Q15056	Eukaryotic translation initiation factor 4H GN=EIF4H PE=1 SV=5	7.27	4
473	Q99832	T-complex protein 1 subunit eta GN=CCT7 PE=1 SV=2	7.22	5
474	Q9UKK9	ADP-sugar pyrophosphatase GN=NUDT5 PE=1 SV=1	7.22	4
475	P06314	Ig kappa chain V-IV region B17 PE=2 SV=1	7.07	16
476	Q8TAX9	Gasdermin-B GN=GSDMB PE=1 SV=2	7.02	4
477	Q96PD5	N-acetylmuramoyl-L-alanine amidase GN=PGLYRP2 PE=1 SV=1	7	5
478	P61970	Nuclear transport factor 2 GN=NUTF2 PE=1 SV=1	7	5
479	Q9Y5P6	Mannose-1-phosphate guanyltransferase beta GN=GMPPB PE=1 SV=2	6.99	4
480	P28070	Proteasome subunit beta type-4 GN=PSMB4 PE=1 SV=4	6.87	6

481	P61981	14-3-3 protein gamma GN=YWHAG PE=1 SV=2	6.8	10
482	P34096	Ribonuclease 4 GN=RNASE4 PE=1 SV=3	6.77	4
483	P24666	Low molecular weight phosphotyrosine protein phosphatase GN=ACP1 PE=1 SV=3	6.76	4
484	P09228	Cystatin-SA GN=CST2 PE=1 SV=1	6.75	32
485	Q14764	Major vault protein GN=MVP PE=1 SV=4	6.73	4
486	Q07960	Rho GTPase-activating protein 1 GN=ARHGAP1 PE=1 SV=1	6.69	4
487	P19338	Nucleolin GN=NCL PE=1 SV=3	6.68	5
488	P62826	GTP-binding nuclear protein Ran GN=RAN PE=1 SV=3	6.68	4
489	P48668	Keratin, type II cytoskeletal 6C GN=KRT6C PE=1 SV=3	6.68	10
490	P29508	Serpin B3 GN=SERPINB3 PE=1 SV=2	6.66	6
491	P00491	Purine nucleoside phosphorylase GN=PNP PE=1 SV=2	6.61	4
492	P59998	Actin-related protein 2/3 complex subunit 4 GN=ARPC4 PE=1 SV=3	6.6	5
493	P62714	Serine/threonine-protein phosphatase 2A catalytic subunit beta isoform GN=PPP2CB PE=1 SV=1	6.6	5
494	P61019	Ras-related protein Rab-2A GN=RAB2A PE=1 SV=1	6.59	4
495	Q8WWI1	LIM domain only protein 7 GN=LMO7 PE=1 SV=3	6.59	4
496	Q9NR45	Sialic acid synthase GN=NANS PE=1 SV=2	6.57	5
497	P62979	Ubiquitin-40S ribosomal protein S27a GN=RPS27A PE=1 SV=2	6.56	5
498	P49354	Protein farnesyltransferase/geranylgeranyltransferase type-1 subunit alpha GN=FNTA PE=1 SV=1	6.55	4
499	P61758	Prefoldin subunit 3 GN=VBP1 PE=1 SV=3	6.55	4

500	P09210	Glutathione S-transferase A2 GN=GSTA2 PE=1 SV=4	6.51	4
501	P32455	Interferon-induced guanylate-binding protein 1 GN=GBP1 PE=1 SV=2	6.42	3
502	Q99954	Submaxillary gland androgen-regulated protein 3A GN=SMR3A PE=2 SV=2	6.39	8
503	P25786	Proteasome subunit alpha type-1 GN=PSMA1 PE=1 SV=1	6.38	5
504	Q9ULA0	Aspartyl aminopeptidase GN=DNPEP PE=1 SV=1	6.3	4
505	O43242	26S proteasome non-ATPase regulatory subunit 3 GN=PSMD3 PE=1 SV=2	6.25	4
506	O95747	Serine/threonine-protein kinase OSR1 GN=OXSR1 PE=1 SV=1	6.25	3
507	Q14103	Heterogeneous nuclear ribonucleoprotein D0 GN=HNRNPD PE=1 SV=1	6.21	3
508	P49591	Serine--tRNA ligase, cytoplasmic GN=SARS PE=1 SV=3	6.18	4
509	P30048	Thioredoxin-dependent peroxide reductase, mitochondrial GN=PRDX3 PE=1 SV=3	6.14	5
510	Q3LXA3	Bifunctional ATP-dependent dihydroxyacetone kinase/FAD-AMP lyase (cyclizing) GN=DAK PE=1 SV=2	6.11	4
511	Q96NY7	Chloride intracellular channel protein 6 GN=CLIC6 PE=2 SV=3	6.1	4
512	P61204	ADP-ribosylation factor 3 GN=ARF3 PE=1 SV=2	6.09	5
513	P26641	Elongation factor 1-gamma GN=EEF1G PE=1 SV=3	6.08	3
514	Q13510	Acid ceramidase GN=ASAH1 PE=1 SV=5	6.08	4
515	P68036	Ubiquitin-conjugating enzyme E2 L3 GN=UBE2L3 PE=1 SV=1	6.06	5
516	O95865	N(G),N(G)-dimethylarginine dimethylaminohydrolase 2 GN=DDAH2 PE=1 SV=1	6.05	4

517	P31146	Coronin-1A GN=CORO1A PE=1 SV=4	6.04	3
518	P52895	Aldo-keto reductase family 1 member C2 GN=AKR1C2 PE=1 SV=3	6.04	21
519	P03973	Antileukoproteinase GN=SLPI PE=1 SV=2	6.03	5
520	Q9Y490	Talin-1 GN=TLN1 PE=1 SV=3	6.03	3
521	P63010	AP-2 complex subunit beta GN=AP2B1 PE=1 SV=1	6.03	4
522	Q6MZM9	Proline-rich protein 27 GN=PRR27 PE=1 SV=1	6.02	5
523	P01776	Ig heavy chain V-III region WAS PE=1 SV=1	6.02	20
524	P62942	Peptidyl-prolyl cis-trans isomerase FKBP1A GN=FKBP1A PE=1 SV=2	6.01	5
525	P61086	Ubiquitin-conjugating enzyme E2 K GN=UBE2K PE=1 SV=3	6.01	3
526	Q13561	Dynactin subunit 2 GN=DCTN2 PE=1 SV=4	6.01	3
527	P35221	Catenin alpha-1 GN=CTNNA1 PE=1 SV=1	6.01	3
528	Q9NTX5	Ethylmalonyl-CoA decarboxylase GN=ECHDC1 PE=1 SV=2	6.01	3
529	Q16543	Hsp90 co-chaperone Cdc37 GN=CDC37 PE=1 SV=1	6.01	3
530	Q7Z7M9	Polypeptide N-acetylgalactosaminyltransferase 5 GN=GALNT5 PE=1 SV=1	6.01	3
531	P01602	Ig kappa chain V-I region HK102 (Fragment) GN=IGKV1-5 PE=4 SV=1	6.01	6
532	P01717	Ig lambda chain V-IV region Hil PE=1 SV=1	6	4
533	P30050	60S ribosomal protein L12 GN=RPL12 PE=1 SV=1	6	3
534	P58546	Myotrophin GN=MTPN PE=1 SV=2	6	4
535	P08294	Extracellular superoxide dismutase [Cu-Zn] GN=SOD3 PE=1 SV=2	6	3
536	P06331	Ig heavy chain V-II region ARH-77 PE=4 SV=1	6	5

537	Q9P1F3	Costars family protein ABRACL GN=ABRACL PE=1 SV=1	6	6
538	P04430	Ig kappa chain V-I region BAN PE=1 SV=1	6	9
539	P49327	Fatty acid synthase GN=FASN PE=1 SV=3	6	4
540	P62888	60S ribosomal protein L30 GN=RPL30 PE=1 SV=2	6	3
541	P01780	Ig heavy chain V-III region JON PE=1 SV=1	6	9
542	P62837	Ubiquitin-conjugating enzyme E2 D2 GN=UBE2D2 PE=1 SV=1	6	3
543	P04220	Ig mu heavy chain disease protein PE=1 SV=1	6	27
544	P13693	Translationally-controlled tumor protein GN=TPT1 PE=1 SV=1	6	3
545	P10768	S-formylglutathione hydrolase GN=ESD PE=1 SV=2	6	3
546	P04040	Catalase GN=CAT PE=1 SV=3	6	3
547	P61020	Ras-related protein Rab-5B GN=RAB5B PE=1 SV=1	6	3
548	P06753	Tropomyosin alpha-3 chain GN=TPM3 PE=1 SV=2	6	5
549	P61916	Epididymal secretory protein E1 GN=NPC2 PE=1 SV=1	6	4
550	Q5SRE7	Phytanoyl-CoA dioxygenase domain-containing protein 1 GN=PHYHD1 PE=1 SV=2	6	3
551	P07711	Cathepsin L1 GN=CTSL PE=1 SV=2	6	3
552	P63208	S-phase kinase-associated protein 1 GN=SKP1 PE=1 SV=2	6	3
553	P40306	Proteasome subunit beta type-10 GN=PSMB10 PE=1 SV=1	6	4
554	O75363	Breast carcinoma-amplified sequence 1 GN=BCAS1 PE=1 SV=2	6	3
555	Q99574	Neuroserpin GN=SERPINI1 PE=1 SV=1	6	3
556	Q9H299	SH3 domain-binding glutamic acid-rich-like	6	3

		protein 3 GN=SH3BGRL3 PE=1 SV=1		
557	P61163	Alpha-centractin GN=ACTR1A PE=1 SV=1	6	3
558	P31943	Heterogeneous nuclear ribonucleoprotein H GN=HNRNPH1 PE=1 SV=4	6	3
559	P55327	Tumor protein D52 GN=TPD52 PE=1 SV=2	6	3
560	Q8N335	Glycerol-3-phosphate dehydrogenase 1-like protein GN=GPD1L PE=1 SV=1	6	3
561	Q99598	Translin-associated protein X GN=TSNAX PE=1 SV=1	6	3
562	Q14011	Cold-inducible RNA-binding protein GN=CIRBP PE=1 SV=1	6	3
563	P20930	Filaggrin GN=FLG PE=1 SV=3	6	3
564	P46976	Glycogenin-1 GN=GYG1 PE=1 SV=4	6	3
565	Q9BS40	Latexin GN=LXN PE=1 SV=2	6	3
566	Q15642	Cdc42-interacting protein 4 GN=TRIP10 PE=1 SV=3	6	3
567	P22102	Trifunctional purine biosynthetic protein adenosine-3 GN=GART PE=1 SV=1	6	3
568	Q9UBG3	Cornulin GN=CRNN PE=1 SV=1	6	3
569	P24158	Myeloblastin GN=PRTN3 PE=1 SV=3	6	3
570	P62834	Ras-related protein Rap-1A GN=RAP1A PE=1 SV=1	6	3
571	P16403	Histone H1.2 GN=HIST1H1C PE=1 SV=2	6	3
572	P62805	Histone H4 GN=HIST1H4A PE=1 SV=2	6	3
573	P51148	Ras-related protein Rab-5C GN=RAB5C PE=1 SV=2	6	3
574	Q9NRX4	14 kDa phosphohistidine phosphatase GN=PHPT1 PE=1 SV=1	6	3
575	P49419	Alpha-aminoadipic semialdehyde dehydrogenase GN=ALDH7A1 PE=1 SV=5	6	3
576	Q8IZP2	Putative protein FAM10A4 GN=ST13P4 PE=5	6	4

		SV=1		
577	O60784	Target of Myb protein 1 GN=TOM1 PE=1 SV=2	6	3
578	Q9BRK5	45 kDa calcium-binding protein GN=SDF4 PE=1 SV=1	5.94	3
579	O60888	Protein CutA GN=CUTA PE=1 SV=2	5.93	4
580	P07686	Beta-hexosaminidase subunit beta GN=HEXB PE=1 SV=3	5.92	3
581	P02753	Retinol-binding protein 4 GN=RBP4 PE=1 SV=3	5.89	4
582	P02533	Keratin, type I cytoskeletal 14 GN=KRT14 PE=1 SV=4	5.89	11
583	P07996	Thrombospondin-1 GN=THBS1 PE=1 SV=2	5.83	4
584	O15511	Actin-related protein 2/3 complex subunit 5 GN=ARPC5 PE=1 SV=3	5.82	3
585	P61224	Ras-related protein Rap-1b GN=RAP1B PE=1 SV=1	5.8	3
586	Q9BZQ8	Protein Niban GN=FAM129A PE=1 SV=1	5.77	5
587	P22234	Multifunctional protein ADE2 GN=PAICS PE=1 SV=3	5.74	4
588	P25774	Cathepsin S GN=CTSS PE=1 SV=3	5.73	4
589	P00505	Aspartate aminotransferase, mitochondrial GN=GOT2 PE=1 SV=3	5.72	3
590	Q5TDH0	Protein DDI1 homolog 2 GN=DDI2 PE=1 SV=1	5.72	4
591	Q14847	LIM and SH3 domain protein 1 GN=LASP1 PE=1 SV=2	5.7	3
592	P04003	C4b-binding protein alpha chain GN=C4BPA PE=1 SV=2	5.7	3
593	P20591	Interferon-induced GTP-binding protein Mx1 GN=MX1 PE=1 SV=4	5.7	3
594	P05120	Plasminogen activator inhibitor 2 GN=SERPINB2 PE=1 SV=2	5.69	5

595	P63000	Ras-related C3 botulinum toxin substrate 1 GN=RAC1 PE=1 SV=1	5.66	4
596	Q8NCW5	NAD(P)H-hydrate epimerase GN=APOA1BP PE=1 SV=2	5.66	3
597	Q9H4G0	Band 4.1-like protein 1 GN=EPB41L1 PE=1 SV=2	5.64	3
598	P62491	Ras-related protein Rab-11A GN=RAB11A PE=1 SV=3	5.6	4
599	P05455	Lupus La protein GN=SSB PE=1 SV=2	5.54	3
600	P26038	Moesin GN=MSN PE=1 SV=3	5.45	13
601	Q96HN2	Putative adenosylhomocysteinase 3 GN=AHCYL2 PE=1 SV=1	5.41	4
602	O15305	Phosphomannomutase 2 GN=PMM2 PE=1 SV=1	5.37	3
603	Q07654	Trefoil factor 3 GN=TFF3 PE=1 SV=1	5.36	3
604	Q96CN7	Isochorismatase domain-containing protein 1 GN=ISOC1 PE=1 SV=3	5.28	3
605	Q92817	Envoplakin GN=EVPL PE=1 SV=3	5.28	3
606	Q9H444	Charged multivesicular body protein 4b GN=CHMP4B PE=1 SV=1	5.25	4
607	Q12765	Secernin-1 GN=SCRN1 PE=1 SV=2	5.25	4
608	P11940	Polyadenylate-binding protein 1 GN=PABPC1 PE=1 SV=2	5.18	3
609	Q9BPY8	Homeodomain-only protein GN=HOPX PE=1 SV=1	5.17	3
610	P00568	Adenylate kinase isoenzyme 1 GN=AK1 PE=1 SV=3	5.15	4
611	Q96L46	Calpain small subunit 2 GN=CAPNS2 PE=2 SV=2	5.1	4
612	P68366	Tubulin alpha-4A chain GN=TUBA4A PE=1 SV=1	5.05	15
613	Q9UMR2	ATP-dependent RNA helicase DDX19B GN=DDX19B PE=1 SV=1	5.02	3

614	Q9H6S3	Epidermal growth factor receptor kinase substrate 8-like protein 2 GN=EPS8L2 PE=1 SV=2	4.99	3
615	Q86X76	Nitrilase homolog 1 GN=NIT1 PE=1 SV=2	4.93	3
616	P01762	Ig heavy chain V-III region TRO PE=1 SV=1	4.92	13
617	O15231	Zinc finger protein 185 GN=ZNF185 PE=1 SV=3	4.89	3
618	P68871	Hemoglobin subunit beta GN=HBB PE=1 SV=2	4.87	3
619	O95372	Acyl-protein thioesterase 2 GN=LYPLA2 PE=1 SV=1	4.75	3
620	P23396	40S ribosomal protein S3 GN=RPS3 PE=1 SV=2	4.74	4
621	Q9BT78	COP9 signalosome complex subunit 4 GN=COPS4 PE=1 SV=1	4.73	3
622	P61604	10 kDa heat shock protein, mitochondrial GN=HSPE1 PE=1 SV=2	4.7	3
623	Q99584	Protein S100-A13 GN=S100A13 PE=1 SV=1	4.68	3
624	P51858	Hepatoma-derived growth factor GN=HDGF PE=1 SV=1	4.67	3
625	Q8TCD5	5'(3')-deoxyribonucleotidase, cytosolic type GN=NT5C PE=1 SV=2	4.64	3
626	P52597	Heterogeneous nuclear ribonucleoprotein F GN=HNRNPF PE=1 SV=3	4.61	3
627	Q04323	UBX domain-containing protein 1 GN=UBXN1 PE=1 SV=2	4.6	4
628	O75608	Acyl-protein thioesterase 1 GN=LYPLA1 PE=1 SV=1	4.59	3
629	P06865	Beta-hexosaminidase subunit alpha GN=HEXA PE=1 SV=2	4.55	3
630	P47897	Glutamine--tRNA ligase GN=QARS PE=1 SV=1	4.54	3
631	P02743	Serum amyloid P-component GN=APCS PE=1 SV=2	4.54	3

632	Q15907	Ras-related protein Rab-11B GN=RAB11B PE=1 SV=4	4.53	3
633	P14780	Matrix metalloproteinase-9 GN=MMP9 PE=1 SV=3	4.53	3
634	O95361	Tripartite motif-containing protein 16 GN=TRIM16 PE=1 SV=3	4.49	3
635	P41250	Glycine--tRNA ligase GN=GARS PE=1 SV=3	4.47	3
636	Q8TE77	Protein phosphatase Slingshot homolog 3 GN=SSH3 PE=1 SV=2	4.45	3
637	Q16651	Prostasin GN=PRSS8 PE=1 SV=1	4.42	4
638	P30040	Endoplasmic reticulum resident protein 29 GN=ERP29 PE=1 SV=4	4.42	2
639	Q13838	Spliceosome RNA helicase DDX39B GN=DDX39B PE=1 SV=1	4.39	3
640	P62495	Eukaryotic peptide chain release factor subunit 1 GN=ETF1 PE=1 SV=3	4.36	3
641	P12081	Histidine--tRNA ligase, cytoplasmic GN=HARS PE=1 SV=2	4.35	2
642	P35998	26S protease regulatory subunit 7 GN=PSMC2 PE=1 SV=3	4.34	3
643	O95394	Phosphoacetylglucosamine mutase GN=PGM3 PE=1 SV=1	4.34	3
644	P06316	Ig lambda chain V-I region BL2 PE=2 SV=1	4.3	3
645	Q16222	UDP-N-acetylhexosamine pyrophosphorylase GN=UAP1 PE=1 SV=3	4.29	2
646	O76003	Glutaredoxin-3 GN=GLRX3 PE=1 SV=2	4.29	2
647	Q08257	Quinone oxidoreductase GN=CRYZ PE=1 SV=1	4.27	3
648	P01611	Ig kappa chain V-I region Wes PE=1 SV=1	4.27	8
649	Q16772	Glutathione S-transferase A3 GN=GSTA3 PE=1 SV=3	4.25	4
650	P04066	Tissue alpha-L-fucosidase GN=FUCA1 PE=1 SV=4	4.24	3

651	Q96HE7	ERO1-like protein alpha GN=ERO1L PE=1 SV=2	4.23	3
652	Q9UGI8	Testin GN=TES PE=1 SV=1	4.21	2
653	P14868	Aspartate--tRNA ligase, cytoplasmic GN=DARS PE=1 SV=2	4.2	2
654	P08697	Alpha-2-antiplasmin GN=SERPINF2 PE=1 SV=3	4.18	2
655	O75436	Vacuolar protein sorting-associated protein 26A GN=VPS26A PE=1 SV=2	4.18	4
656	Q9UQ80	Proliferation-associated protein 2G4 GN=PA2G4 PE=1 SV=3	4.16	3
657	P29218	Inositol monophosphatase 1 GN=IMPA1 PE=1 SV=1	4.14	2
658	P04207	Ig kappa chain V-III region CLL PE=1 SV=2	4.13	7
659	P13987	CD59 glycoprotein GN=CD59 PE=1 SV=1	4.13	3
660	P50238	Cysteine-rich protein 1 GN=CRIP1 PE=1 SV=3	4.13	3
661	Q9H361	Polyadenylate-binding protein 3 GN=PABPC3 PE=1 SV=2	4.12	2
662	P30047	GTP cyclohydrolase 1 feedback regulatory protein GN=GCHFR PE=1 SV=3	4.11	3
663	Q15257	Serine/threonine-protein phosphatase 2A activator GN=PPP2R4 PE=1 SV=3	4.08	3
664	P48594	Serpin B4 GN=SERPINB4 PE=1 SV=2	4.07	4
665	Q9NYL9	Tropomodulin-3 GN=TMOD3 PE=1 SV=1	4.07	2
666	P53367	Arfaptin-1 GN=ARFIP1 PE=1 SV=2	4.07	2
667	P01605	Ig kappa chain V-I region Lay PE=1 SV=1	4.07	5
668	Q9HAB8	Phosphopantothenate--cysteine ligase GN=PPCS PE=1 SV=2	4.06	3
669	Q7Z4W1	L-xylulose reductase GN=DCXR PE=1 SV=2	4.06	3
670	E9PAV3	Nascent polypeptide-associated complex subunit alpha, muscle-specific form	4.05	3

		GN=NACA PE=1 SV=1		
671	P51884	Lumican GN=LUM PE=1 SV=2	4.04	2
672	P60763	Ras-related C3 botulinum toxin substrate 3 GN=RAC3 PE=1 SV=1	4.04	2
673	P05386	60S acidic ribosomal protein P1 GN=RPLP1 PE=1 SV=1	4.03	5
674	O15145	Actin-related protein 2/3 complex subunit 3 GN=ARPC3 PE=1 SV=3	4.03	3
675	P39687	Acidic leucine-rich nuclear phosphoprotein 32 family member A GN=ANP32A PE=1 SV=1	4.03	2
676	P81605	Dermcidin GN=DCD PE=1 SV=2	4.02	2
677	P29466	Caspase-1 GN=CASP1 PE=1 SV=1	4.02	3
678	P84085	ADP-ribosylation factor 5 GN=ARF5 PE=1 SV=2	4.02	2
679	P06311	Ig kappa chain V-III region IARC/BL41 PE=1 SV=1	4.02	13
680	Q9BWS9	Chitinase domain-containing protein 1 GN=CHID1 PE=1 SV=1	4.02	2
681	O60610	Protein diaphanous homolog 1 GN=DIAPH1 PE=1 SV=2	4.02	2
682	P42224	Signal transducer and activator of transcription 1-alpha/beta GN=STAT1 PE=1 SV=2	4.02	2
683	Q13200	26S proteasome non-ATPase regulatory subunit 2 GN=PSMD2 PE=1 SV=3	4.01	2
684	Q9NP97	Dynein light chain roadblock-type 1 GN=DYNLRB1 PE=1 SV=3	4.01	2
685	P01699	Ig lambda chain V-I region VOR PE=1 SV=1	4.01	3
686	A0M8Q6	Ig lambda-7 chain C region GN=IGLC7 PE=1 SV=2	4.01	34
687	Q9NT62	Ubiquitin-like-conjugating enzyme ATG3 GN=ATG3 PE=1 SV=1	4.01	2
688	P12830	Cadherin-1 GN=CDH1 PE=1 SV=3	4.01	2

689	P07477	Trypsin-1 GN=PRSS1 PE=1 SV=1	4.01	6
690	O75348	V-type proton ATPase subunit G 1 GN=ATP6V1G1 PE=1 SV=3	4.01	2
691	O60506	Heterogeneous nuclear ribonucleoprotein Q GN=SYNCRIP PE=1 SV=2	4	2
692	Q15847	Adipogenesis regulatory factor GN=ADIRF PE=1 SV=1	4	5
693	Q96FQ6	Protein S100-A16 GN=S100A16 PE=1 SV=1	4	2
694	Q92882	Osteoclast-stimulating factor 1 GN=OSTF1 PE=1 SV=2	4	2
695	O60936	Nucleolar protein 3 GN=NOL3 PE=1 SV=1	4	2
696	Q15819	Ubiquitin-conjugating enzyme E2 variant 2 GN=UBE2V2 PE=1 SV=4	4	2
697	Q15121	Astrocytic phosphoprotein PEA-15 GN=PEA15 PE=1 SV=2	4	3
698	P55209	Nucleosome assembly protein 1-like 1 GN=NAP1L1 PE=1 SV=1	4	2
699	P25325	3-mercaptopyruvate sulfurtransferase GN=MPST PE=1 SV=3	4	2
700	P06319	Ig lambda chain V-VI region EB4 PE=2 SV=1	4	5
701	P61457	Pterin-4-alpha-carbinolamine dehydratase GN=PCBD1 PE=1 SV=2	4	3
702	Q8N1G4	Leucine-rich repeat-containing protein 47 GN=LRRC47 PE=1 SV=1	4	2
703	Q9BUT1	3-hydroxybutyrate dehydrogenase type 2 GN=BDH2 PE=1 SV=2	4	3
704	P25398	40S ribosomal protein S12 GN=RPS12 PE=1 SV=3	4	4
705	O95782	AP-2 complex subunit alpha-1 GN=AP2A1 PE=1 SV=3	4	2
706	P35321	Cornifin-A GN=SPRR1A PE=1 SV=2	4	2
707	Q9H2G2	STE20-like serine/threonine-protein kinase GN=SLK PE=1 SV=1	4	2

708	O75347	Tubulin-specific chaperone A GN=TBCA PE=1 SV=3	4	2
709	P52272	Heterogeneous nuclear ribonucleoprotein M GN=HNRNPM PE=1 SV=3	4	2
710	Q99471	Prefoldin subunit 5 GN=PFDN5 PE=1 SV=2	4	2
711	Q9UK22	F-box only protein 2 GN=FBXO2 PE=1 SV=2	4	2
712	P01767	Ig heavy chain V-III region BUT PE=1 SV=1	4	9
713	P01613	Ig kappa chain V-I region Ni PE=1 SV=1	4	10
714	Q96QK1	Vacuolar protein sorting-associated protein 35 GN=VPS35 PE=1 SV=2	4	2
715	Q9BW30	Tubulin polymerization-promoting protein family member 3 GN=TPPP3 PE=1 SV=1	4	2
716	P54725	UV excision repair protein RAD23 homolog A GN=RAD23A PE=1 SV=1	4	2
717	Q13740	CD166 antigen GN=ALCAM PE=1 SV=2	4	3
718	Q02878	60S ribosomal protein L6 GN=RPL6 PE=1 SV=3	4	2
719	Q5VW32	BRO1 domain-containing protein BROX GN=BROX PE=1 SV=1	4	2
720	Q9GZT8	Putative GTP cyclohydrolase 1 type 2 NIF3L1 GN=NIF3L1 PE=1 SV=2	4	2
721	Q9HBR0	Putative sodium-coupled neutral amino acid transporter 10 GN=SLC38A10 PE=1 SV=2	4	2
722	Q14677	Clathrin interactor 1 GN=CLINT1 PE=1 SV=1	4	2
723	Q15046	Lysine--tRNA ligase GN=KARS PE=1 SV=3	4	2
724	P17980	26S protease regulatory subunit 6A GN=PSMC3 PE=1 SV=3	4	2
725	Q99460	26S proteasome non-ATPase regulatory subunit 1 GN=PSMD1 PE=1 SV=2	4	2
726	Q14118	Dystroglycan GN=DAG1 PE=1 SV=2	4	2
727	P09417	Dihydropteridine reductase GN=QDPR PE=1 SV=2	4	2

728	Q9NUQ9	Protein FAM49B GN=FAM49B PE=1 SV=1	4	2
729	Q11201	CMP-N-acetylneuraminate-beta-galactosamide-alpha-2,3-sialyltransferase 1 GN=ST3GAL1 PE=2 SV=1	4	2
730	P62081	40S ribosomal protein S7 GN=RPS7 PE=1 SV=1	4	2
731	P31944	Caspase-14 GN=CASP14 PE=1 SV=2	4	3
732	P02747	Complement C1q subcomponent subunit C GN=C1QC PE=1 SV=3	4	2
733	NA	Biognosys Retention time kit peptide	4	4
734	P62857	40S ribosomal protein S28 GN=RPS28 PE=1 SV=1	4	2
735	P01714	Ig lambda chain V-III region SH PE=1 SV=1	4	4
736	NA	Biognosys Retention time kit peptide	4	3
737	NA	Biognosys Retention time kit peptide	4	2
738	P22528	Cornifin-B GN=SPRR1B PE=1 SV=2	4	3
739	P24534	Elongation factor 1-beta GN=EEF1B2 PE=1 SV=3	4	2
740	P41226	Ubiquitin-like modifier-activating enzyme 7 GN=UBA7 PE=1 SV=2	4	2
741	P16402	Histone H1.3 GN=HIST1H1D PE=1 SV=2	4	2
742	P09758	Tumor-associated calcium signal transducer 2 GN=TACSTD2 PE=1 SV=3	4	2
743	P01763	Ig heavy chain V-III region WEA PE=1 SV=1	4	7
744	P26639	Threonine--tRNA ligase, cytoplasmic GN=TARS PE=1 SV=3	4	2
745	Q9H788	SH2 domain-containing protein 4A GN=SH2D4A PE=1 SV=1	4	2
746	O60749	Sorting nexin-2 GN=SNX2 PE=1 SV=2	4	3
747	Q8N1Q1	Carbonic anhydrase 13 GN=CA13 PE=1 SV=1	4	3
748	P54819	Adenylate kinase 2, mitochondrial GN=AK2	4	3

		PE=1 SV=2		
749	Q92896	Golgi apparatus protein 1 GN=GLG1 PE=1 SV=2	4	2
750	Q53EL6	Programmed cell death protein 4 GN=PDCD4 PE=1 SV=2	4	2
751	P55735	Protein SEC13 homolog GN=SEC13 PE=1 SV=3	4	2
752	P22059	Oxysterol-binding protein 1 GN=OSBP PE=1 SV=1	4	2
753	Q15019	Septin-2 GN=SEPT2 PE=1 SV=1	4	2
754	P35813	Protein phosphatase 1A GN=PPM1A PE=1 SV=1	4	2
755	P63220	40S ribosomal protein S21 GN=RPS21 PE=1 SV=1	4	3
756	Q16531	DNA damage-binding protein 1 GN=DDB1 PE=1 SV=1	4	2
757	Q02790	Peptidyl-prolyl cis-trans isomerase FKBP4 GN=FKBP4 PE=1 SV=3	4	2
758	P30626	Sorcin GN=SRI PE=1 SV=1	4	3
759	Q8TE68	Epidermal growth factor receptor kinase substrate 8-like protein 1 GN=EPS8L1 PE=1 SV=3	4	2
760	Q8NBS9	Thioredoxin domain-containing protein 5 GN=TXNDC5 PE=1 SV=2	4	2
761	Q15631	Translin GN=TSN PE=1 SV=1	4	2
762	P61077	Ubiquitin-conjugating enzyme E2 D3 GN=UBE2D3 PE=1 SV=1	4	2
763	Q9NZ08	Endoplasmic reticulum aminopeptidase 1 GN=ERAP1 PE=1 SV=3	4	2
764	P02794	Ferritin heavy chain GN=FTH1 PE=1 SV=2	4	2
765	Q13404	Ubiquitin-conjugating enzyme E2 variant 1 GN=UBE2V1 PE=1 SV=2	4	2
766	B0FP48	Uroplakin-3b-like protein GN=UPK3BL PE=2	4	2

		SV=1		
767	P08246	Neutrophil elastase GN=ELANE PE=1 SV=1	4	2
768	Q15185	Prostaglandin E synthase 3 GN=PTGES3 PE=1 SV=1	4	3
769	P01619	Ig kappa chain V-III region B6 PE=1 SV=1	4	14
770	Q8NCL4	Polypeptide N-acetylgalactosaminyltransferase 6 GN=GALNT6 PE=2 SV=2	4	2
771	P54136	Arginine--tRNA ligase, cytoplasmic GN=RARS PE=1 SV=2	4	2
772	Q14002	Carcinoembryonic antigen-related cell adhesion molecule 7 GN=CEACAM7 PE=1 SV=1	4	2
773	P43487	Ran-specific GTPase-activating protein GN=RANBP1 PE=1 SV=1	4	2
774	Q14203	Dynactin subunit 1 GN=DCTN1 PE=1 SV=3	4	2
775	P02545	Prelamin-A/C GN=LMNA PE=1 SV=1	4	2
776	P25705	ATP synthase subunit alpha, mitochondrial GN=ATP5A1 PE=1 SV=1	4	2
777	P14854	Cytochrome c oxidase subunit 6B1 GN=COX6B1 PE=1 SV=2	4	2
778	P53634	Dipeptidyl peptidase 1 GN=CTSC PE=1 SV=2	4	2
779	P80419	Ig heavy chain V-III region GAR PE=1 SV=1	4	6
780	P53621	Coatomer subunit alpha GN=COPA PE=1 SV=2	4	3
781	P54577	Tyrosine--tRNA ligase, cytoplasmic GN=YARS PE=1 SV=4	4	2
782	P60983	Glia maturation factor beta GN=GMFB PE=1 SV=2	4	2
783	P55795	Heterogeneous nuclear ribonucleoprotein H2 GN=HNRNPH2 PE=1 SV=1	4	2
784	P41222	Prostaglandin-H2 D-isomerase GN=PTGDS PE=1 SV=1	4	2

785	Q9BTM1	Histone H2A.J GN=H2AFJ PE=1 SV=1	4	2
786	P06681	Complement C2 GN=C2 PE=1 SV=2	4	2
787	P26599	Polypyrimidine tract-binding protein 1 GN=PTBP1 PE=1 SV=1	4	2
788	P55064	Aquaporin-5 GN=AQP5 PE=1 SV=1	4	2
789	P19957	Elafin GN=PI3 PE=1 SV=3	4	2
790	O75131	Copine-3 GN=CPNE3 PE=1 SV=1	4	2
791	P50914	60S ribosomal protein L14 GN=RPL14 PE=1 SV=4	4	2
792	P33176	Kinesin-1 heavy chain GN=KIF5B PE=1 SV=1	4	2
793	P02144	Myoglobin GN=MB PE=1 SV=2	4	2
794	P11586	C-1-tetrahydrofolate synthase, cytoplasmic GN=MTHFD1 PE=1 SV=3	4	2
795	Q99798	Aconitate hydratase, mitochondrial GN=ACO2 PE=1 SV=2	4	2
796	P68032	Actin, alpha cardiac muscle 1 GN=ACTC1 PE=1 SV=1	4	32
797	P05198	Eukaryotic translation initiation factor 2 subunit 1 GN=EIF2S1 PE=1 SV=3	4	2
798	Q16204	Coiled-coil domain-containing protein 6 GN=CCDC6 PE=1 SV=2	4	2
799	P67809	Nuclease-sensitive element-binding protein 1 GN=YBX1 PE=1 SV=3	4	2
800	P20339	Ras-related protein Rab-5A GN=RAB5A PE=1 SV=2	4	2
801	P15941	Mucin-1 GN=MUC1 PE=1 SV=3	4	2
802	Q99733	Nucleosome assembly protein 1-like 4 GN=NAP1L4 PE=1 SV=1	4	2
803	Q2VIR3	Putative eukaryotic translation initiation factor 2 subunit 3-like protein GN=EIF2S3L PE=5 SV=2	4	2
804	Q9UNM6	26S proteasome non-ATPase regulatory	4	2

		subunit 13 GN=PSMD13 PE=1 SV=2		
805	P08118	Beta-microseminoprotein GN=MSMB PE=1 SV=1	4	2
806	P32456	Interferon-induced guanylate-binding protein 2 GN=GBP2 PE=1 SV=3	4	2
807	Q9HCY8	Protein S100-A14 GN=S100A14 PE=1 SV=1	4	2
808	P35326	Small proline-rich protein 2A GN=SPRR2A PE=1 SV=1	3.96	2
809	P05156	Complement factor I GN=CFI PE=1 SV=2	3.96	2
810	O75882	Attractin GN=ATRN PE=1 SV=2	3.96	2
811	Q9NRV9	Heme-binding protein 1 GN=HEBP1 PE=1 SV=1	3.92	2
812	P46781	40S ribosomal protein S9 GN=RPS9 PE=1 SV=3	3.92	2
813	P28482	Mitogen-activated protein kinase 1 GN=MAPK1 PE=1 SV=3	3.91	5
814	Q9H2U2	Inorganic pyrophosphatase 2, mitochondrial GN=PPA2 PE=1 SV=2	3.89	3
815	P00492	Hypoxanthine-guanine phosphoribosyltransferase GN=HPRT1 PE=1 SV=2	3.89	2
816	P07954	Fumarate hydratase, mitochondrial GN=FH PE=1 SV=3	3.89	2
817	Q9Y2S2	Lambda-crystallin homolog GN=CRYL1 PE=1 SV=3	3.85	2
818	P10412	Histone H1.4 GN=HIST1H1E PE=1 SV=2	3.85	3
819	P31153	S-adenosylmethionine synthase isoform type-2 GN=MAT2A PE=1 SV=1	3.82	2
820	Q9H008	Phospholysine phosphohistidine inorganic pyrophosphate phosphatase GN=LHPP PE=1 SV=2	3.8	2
821	P48444	Coatomer subunit delta GN=ARCN1 PE=1 SV=1	3.78	2

822	P59666	Neutrophil defensin 3 GN=DEFA3 PE=1 SV=1	3.74	3
823	P01814	Ig heavy chain V-II region OU PE=1 SV=1	3.74	2
824	Q96DR8	Mucin-like protein 1 GN=MUCL1 PE=1 SV=1	3.74	3
825	Q92530	Proteasome inhibitor PI31 subunit GN=PSMF1 PE=1 SV=2	3.7	3
826	P01703	Ig lambda chain V-I region NEWM PE=1 SV=1	3.68	3
827	P27361	Mitogen-activated protein kinase 3 GN=MAPK3 PE=1 SV=4	3.64	2
828	Q7Z4S6	Kinesin-like protein KIF21A GN=KIF21A PE=1 SV=2	3.62	2
829	Q99877	Histone H2B type 1-N GN=HIST1H2BN PE=1 SV=3	3.62	2
830	P13861	cAMP-dependent protein kinase type II-alpha regulatory subunit GN=PRKAR2A PE=1 SV=2	3.6	3
831	O43399	Tumor protein D54 GN=TPD52L2 PE=1 SV=2	3.59	2
832	P28065	Proteasome subunit beta type-9 GN=PSMB9 PE=1 SV=2	3.58	2
833	P15374	Ubiquitin carboxyl-terminal hydrolase isozyme L3 GN=UCHL3 PE=1 SV=1	3.57	2
834	P41091	Eukaryotic translation initiation factor 2 subunit 3 GN=EIF2S3 PE=1 SV=3	3.52	2
835	P13671	Complement component C6 GN=C6 PE=1 SV=3	3.51	2
836	P30084	Enoyl-CoA hydratase, mitochondrial GN=ECHS1 PE=1 SV=4	3.51	2
837	O00592	Podocalyxin GN=PODXL PE=1 SV=2	3.42	2
838	P08185	Corticosteroid-binding globulin GN=SERPINA6 PE=1 SV=1	3.41	2
839	Q15691	Microtubule-associated protein RP/EB family member 1 GN=MAPRE1 PE=1 SV=3	3.38	2
840	Q6PCB0	von Willebrand factor A domain-containing protein 1 GN=VWA1 PE=2 SV=1	3.37	2

841	Q13296	Mammaglobin-A GN=SCGB2A2 PE=1 SV=1	3.37	2
842	P12259	Coagulation factor V GN=F5 PE=1 SV=4	3.36	2
843	P12277	Creatine kinase B-type GN=CKB PE=1 SV=1	3.34	2
844	Q9H773	dCTP pyrophosphatase 1 GN=DCTPP1 PE=1 SV=1	3.34	2
845	P53041	Serine/threonine-protein phosphatase 5 GN=PPP5C PE=1 SV=1	3.34	2
846	Q9Y4L1	Hypoxia up-regulated protein 1 GN=HYOU1 PE=1 SV=1	3.34	2
847	P15814	Immunoglobulin lambda-like polypeptide 1 GN=IGLL1 PE=1 SV=1	3.33	6
848	Q6DKJ4	Nucleoredoxin GN=NXN PE=1 SV=2	3.33	3
849	P27169	Serum paraoxonase/arylesterase 1 GN=PON1 PE=1 SV=3	3.32	2
850	Q9UHL4	Dipeptidyl peptidase 2 GN=DPP7 PE=1 SV=3	3.31	2
851	Q9H0R4	Haloacid dehalogenase-like hydrolase domain-containing protein 2 GN=HDHD2 PE=1 SV=1	3.31	2
852	P54578	Ubiquitin carboxyl-terminal hydrolase 14 GN=USP14 PE=1 SV=3	3.28	2
853	Q9Y6E0	Serine/threonine-protein kinase 24 GN=STK24 PE=1 SV=1	3.25	2
854	O95833	Chloride intracellular channel protein 3 GN=CLIC3 PE=1 SV=2	3.24	2
855	O95395	Beta-1,3-galactosyl-O-glycosyl-glycoprotein beta-1,6-N-acetylglucosaminyltransferase 3 GN=GCNT3 PE=2 SV=1	3.23	2
856	Q13442	28 kDa heat- and acid-stable phosphoprotein GN=PDAP1 PE=1 SV=1	3.23	2
857	P06888	Ig lambda chain V-I region EPS PE=1 SV=1	3.23	4
858	Q99880	Histone H2B type 1-L GN=HIST1H2BL PE=1 SV=3	3.21	2
859	Q8NI22	Multiple coagulation factor deficiency protein 2	3.19	2

		GN=MCFD2 PE=1 SV=1		
860	P09871	Complement C1s subcomponent GN=C1S PE=1 SV=1	3.19	2
861	P01861	Ig gamma-4 chain C region GN=IGHG4 PE=1 SV=1	3.19	48
862	P13667	Protein disulfide-isomerase A4 GN=PDIA4 PE=1 SV=2	3.15	3
863	O14737	Programmed cell death protein 5 GN=PDCD5 PE=1 SV=3	3.15	2
864	Q9Y266	Nuclear migration protein nudC GN=NUDC PE=1 SV=1	3.15	2
865	Q9BS26	Endoplasmic reticulum resident protein 44 GN=ERP44 PE=1 SV=1	3.09	2
866	Q12805	EGF-containing fibulin-like extracellular matrix protein 1 GN=EFEMP1 PE=1 SV=2	3.05	2
867	Q6IBS0	Twinfilin-2 GN=TWF2 PE=1 SV=2	2.99	2
868	P55011	Solute carrier family 12 member 2 GN=SLC12A2 PE=1 SV=1	2.96	2
869	P04431	Ig kappa chain V-I region Walker PE=1 SV=1	2.96	12
870	P42574	Caspase-3 GN=CASP3 PE=1 SV=2	2.94	2
871	Q8TBC4	NEDD8-activating enzyme E1 catalytic subunit GN=UBA3 PE=1 SV=2	2.92	2
872	P54920	Alpha-soluble NSF attachment protein GN=NAPA PE=1 SV=3	2.92	2
873	P01880	Ig delta chain C region GN=IGHD PE=1 SV=2	2.91	2
874	Q03591	Complement factor H-related protein 1 GN=CFHR1 PE=1 SV=2	2.9	4
875	Q9BY32	Inosine triphosphate pyrophosphatase GN=ITPA PE=1 SV=2	2.9	2
876	P03950	Angiogenin GN=ANG PE=1 SV=1	2.85	2
877	Q00169	Phosphatidylinositol transfer protein alpha isoform GN=PITPNA PE=1 SV=2	2.85	2

878	P52943	Cysteine-rich protein 2 GN=CRIP2 PE=1 SV=1	2.83	2
879	Q8N257	Histone H2B type 3-B GN=HIST3H2BB PE=1 SV=3	2.83	2
880	Q5VT79	Annexin A8-like protein 2 GN=ANXA8L2 PE=2 SV=1	2.78	2
881	P19021	Peptidyl-glycine alpha-amidating monooxygenase GN=PAM PE=1 SV=2	2.77	2
882	Q8WXI7	Mucin-16 GN=MUC16 PE=1 SV=2	2.75	2
883	Q9BV40	Vesicle-associated membrane protein 8 GN=VAMP8 PE=1 SV=1	2.75	2
884	Q15008	26S proteasome non-ATPase regulatory subunit 6 GN=PSMD6 PE=1 SV=1	2.7	2
885	O95861	3'(2'),5'-bisphosphate nucleotidase 1 GN=BPNT1 PE=1 SV=1	2.7	3
886	Q9Y2V2	Calcium-regulated heat stable protein 1 GN=CARHSP1 PE=1 SV=2	2.68	2
887	Q96GG9	DCN1-like protein 1 GN=DCUN1D1 PE=1 SV=1	2.66	2
888	P01597	Ig kappa chain V-I region DEE PE=1 SV=1	2.65	14
889	Q9UDY2	Tight junction protein ZO-2 GN=TJP2 PE=1 SV=2	2.63	2
890	Q12840	Kinesin heavy chain isoform 5A GN=KIF5A PE=1 SV=2	2.59	2
891	P52788	Spermine synthase GN=SMS PE=1 SV=2	2.57	2
892	O14908	PDZ domain-containing protein GIPC1 GN=GIPC1 PE=1 SV=2	2.56	2
893	Q99879	Histone H2B type 1-M GN=HIST1H2BM PE=1 SV=3	2.53	2
894	Q9Y315	Putative deoxyribose-phosphate aldolase GN=DERA PE=1 SV=2	2.52	2
895	O14773	Tripeptidyl-peptidase 1 GN=TPP1 PE=1 SV=2	2.49	2
896	Q15843	NEDD8 GN=NEDD8 PE=1 SV=1	2.48	3

897	P05186	Alkaline phosphatase, tissue-nonspecific isozyme GN=ALPL PE=1 SV=4	2.48	2
898	Q9H3K6	BolA-like protein 2 GN=BOLA2 PE=1 SV=1	2.46	2
899	P22061	Protein-L-isoaspartate(D-aspartate) O-methyltransferase GN=PCMT1 PE=1 SV=4	2.42	2
900	P80217	Interferon-induced 35 kDa protein GN=IFI35 PE=1 SV=5	2.37	2
901	Q8WXG9	G-protein coupled receptor 98 GN=GPR98 PE=1 SV=2	2.36	2
902	P04433	Ig kappa chain V-III region VG (Fragment) PE=1 SV=1	2.35	7
903	O94760	N(G),N(G)-dimethylarginine dimethylaminohydrolase 1 GN=DDAH1 PE=1 SV=3	2.34	3
904	P15559	NAD(P)H dehydrogenase [quinone] 1 GN=NQO1 PE=1 SV=1	2.32	2
905	Q9BV20	Methylthioribose-1-phosphate isomerase GN=MRI1 PE=1 SV=1	2.32	2
906	Q02487	Desmocollin-2 GN=DSC2 PE=1 SV=1	2.31	2
907	Q9Y3C8	Ubiquitin-fold modifier-conjugating enzyme 1 GN=UFC1 PE=1 SV=3	2.31	2
908	Q00341	Vigilin GN=HDLBP PE=1 SV=2	2.31	2
909	O43598	2'-deoxynucleoside 5'-phosphate N-hydrolase 1 GN=DNPH1 PE=1 SV=1	2.3	1
910	Q9Y3F4	Serine-threonine kinase receptor-associated protein GN=STRAP PE=1 SV=1	2.28	2
911	Q12882	Dihydropyrimidine dehydrogenase [NADP(+)] GN=DPYD PE=1 SV=2	2.27	1
912	Q14126	Desmoglein-2 GN=DSG2 PE=1 SV=2	2.26	2
913	P15880	40S ribosomal protein S2 GN=RPS2 PE=1 SV=2	2.25	2
914	P23588	Eukaryotic translation initiation factor 4B GN=EIF4B PE=1 SV=2	2.24	1

915	Q8IWE2	Protein NOXP20 GN=FAM114A1 PE=1 SV=2	2.22	2
916	P53618	Coatomer subunit beta GN=COPB1 PE=1 SV=3	2.21	1
917	Q9BWD1	Acetyl-CoA acetyltransferase, cytosolic GN=ACAT2 PE=1 SV=2	2.2	2
918	Q8NHP1	Aflatoxin B1 aldehyde reductase member 4 GN=AKR7L PE=2 SV=6	2.2	1
919	P55884	Eukaryotic translation initiation factor 3 subunit B GN=EIF3B PE=1 SV=3	2.2	1
920	O43768	Alpha-endosulfine GN=ENSA PE=1 SV=1	2.19	1
921	Q9Y3Z3	Deoxynucleoside triphosphate triphosphohydrolase SAMHD1 GN=SAMHD1 PE=1 SV=2	2.18	1
922	O14907	Tax1-binding protein 3 GN=TAX1BP3 PE=1 SV=2	2.15	2
923	Q9UFH2	Dynein heavy chain 17, axonemal GN=DNAH17 PE=1 SV=2	2.13	2
924	Q15746	Myosin light chain kinase, smooth muscle GN=MYLK PE=1 SV=4	2.11	2
925	Q7L576	Cytoplasmic FMR1-interacting protein 1 GN=CYFIP1 PE=1 SV=1	2.11	1
926	P48556	26S proteasome non-ATPase regulatory subunit 8 GN=PSMD8 PE=1 SV=2	2.09	2
927	Q8NC51	Plasminogen activator inhibitor 1 RNA-binding protein GN=SERBP1 PE=1 SV=2	2.08	1
928	P49588	Alanine--tRNA ligase, cytoplasmic GN=AARS PE=1 SV=2	2.08	2
929	P43686	26S protease regulatory subunit 6B GN=PSMC4 PE=1 SV=2	2.07	2
930	O15212	Prefoldin subunit 6 GN=PFDN6 PE=1 SV=1	2.07	1
931	P08174	Complement decay-accelerating factor GN=CD55 PE=1 SV=4	2.07	1
932	Q32MZ4	Leucine-rich repeat flightless-interacting protein 1 GN=LRRFIP1 PE=1 SV=2	2.06	1

933	Q13867	Bleomycin hydrolase GN=BLMH PE=1 SV=1	2.06	1
934	Q8NI27	THO complex subunit 2 GN=THOC2 PE=1 SV=2	2.06	1
935	P01612	Ig kappa chain V-I region Mev PE=1 SV=1	2.06	4
936	Q96CW1	AP-2 complex subunit mu GN=AP2M1 PE=1 SV=2	2.06	1
937	P46109	Crk-like protein GN=CRKL PE=1 SV=1	2.06	1
938	P53396	ATP-citrate synthase GN=ACLY PE=1 SV=3	2.06	2
939	Q9P2E9	Ribosome-binding protein 1 GN=RRBP1 PE=1 SV=4	2.05	1
940	P14923	Junction plakoglobin GN=JUP PE=1 SV=3	2.04	1
941	Q6UXB2	VEGF coregulated chemokine 1 GN=CXCL17 PE=1 SV=1	2.04	1
942	O14618	Copper chaperone for superoxide dismutase GN=CCS PE=1 SV=1	2.04	1
943	O60763	General vesicular transport factor p115 GN=USO1 PE=1 SV=2	2.04	2
944	Q14019	Coactosin-like protein GN=COTL1 PE=1 SV=3	2.04	1
945	O94973	AP-2 complex subunit alpha-2 GN=AP2A2 PE=1 SV=2	2.04	1
946	Q9UH65	Switch-associated protein 70 GN=SWAP70 PE=1 SV=1	2.04	2
947	P84103	Serine/arginine-rich splicing factor 3 GN=SRSF3 PE=1 SV=1	2.04	1
948	P12109	Collagen alpha-1(VI) chain GN=COL6A1 PE=1 SV=3	2.03	2
949	P35270	Sepiapterin reductase GN=SPR PE=1 SV=1	2.03	1
950	P27695	DNA-(apurinic or apyrimidinic site) lyase GN=APEX1 PE=1 SV=2	2.03	3
951	O94811	Tubulin polymerization-promoting protein GN=TPPP PE=1 SV=1	2.03	2
952	Q96FV2	Secernin-2 GN=SCRN2 PE=1 SV=3	2.03	2

953	O00244	Copper transport protein ATOX1 GN=ATOX1 PE=1 SV=1	2.03	2
954	O00231	26S proteasome non-ATPase regulatory subunit 11 GN=PSMD11 PE=1 SV=3	2.03	2
955	P63096	Guanine nucleotide-binding protein G(i) subunit alpha-1 GN=GNAI1 PE=1 SV=2	2.03	1
956	O43423	Acidic leucine-rich nuclear phosphoprotein 32 family member C GN=ANP32C PE=2 SV=1	2.03	2
957	Q09028	Histone-binding protein RBBP4 GN=RBBP4 PE=1 SV=3	2.03	1
958	P01616	Ig kappa chain V-II region MIL PE=1 SV=1	2.02	12
959	Q00534	Cyclin-dependent kinase 6 GN=CDK6 PE=1 SV=1	2.02	1
960	Q96F07	Cytoplasmic FMR1-interacting protein 2 GN=CYFIP2 PE=1 SV=2	2.02	1
961	O43813	LanC-like protein 1 GN=LANCL1 PE=1 SV=1	2.01	1
962	Q8WZA0	Protein LZIC GN=LZIC PE=1 SV=1	2.01	1
963	P62191	26S protease regulatory subunit 4 GN=PSMC1 PE=1 SV=1	2.01	1
964	O60684	Importin subunit alpha-7 GN=KPNA6 PE=1 SV=1	2.01	2
965	P15170	Eukaryotic peptide chain release factor GTP-binding subunit ERF3A GN=GSPT1 PE=1 SV=1	2.01	2
966	P62906	60S ribosomal protein L10a GN=RPL10A PE=1 SV=2	2.01	1
967	P62333	26S protease regulatory subunit 10B GN=PSMC6 PE=1 SV=1	2.01	2
968	Q9Y6Q5	AP-1 complex subunit mu-2 GN=AP1M2 PE=1 SV=4	2.01	1
969	Q14116	Interleukin-18 GN=IL18 PE=1 SV=1	2.01	1
970	Q15404	Ras suppressor protein 1 GN=RSU1 PE=1 SV=3	2.01	1

971	Q13162	Peroxiredoxin-4 GN=PRDX4 PE=1 SV=1	2.01	9
972	P09668	Pro-cathepsin H GN=CTSH PE=1 SV=4	2.01	1
973	P01040	Cystatin-A GN=CSTA PE=1 SV=1	2.01	1
974	Q8IU85	Calcium/calmodulin-dependent protein kinase type 1D GN=CAMK1D PE=1 SV=1	2.01	1
975	Q9UHD8	Septin-9 GN=SEPT9 PE=1 SV=2	2.01	2
976	A0AVT1	Ubiquitin-like modifier-activating enzyme 6 GN=UBA6 PE=1 SV=1	2.01	2
977	Q9Y263	Phospholipase A-2-activating protein GN=PLAA PE=1 SV=2	2.01	1
978	P36543	V-type proton ATPase subunit E 1 GN=ATP6V1E1 PE=1 SV=1	2.01	1
979	Q9NZL9	Methionine adenosyltransferase 2 subunit beta GN=MAT2B PE=1 SV=1	2.01	2
980	Q15327	Ankyrin repeat domain-containing protein 1 GN=ANKRD1 PE=1 SV=2	2.01	1
981	Q9UPS8	Ankyrin repeat domain-containing protein 26 GN=ANKRD26 PE=1 SV=3	2.01	1
982	P01860	Ig gamma-3 chain C region GN=IGHG3 PE=1 SV=2	2	50
983	P01770	Ig heavy chain V-III region NIE PE=1 SV=1	2	9
984	Q14152	Eukaryotic translation initiation factor 3 subunit A GN=EIF3A PE=1 SV=1	2	1
985	P19367	Hexokinase-1 GN=HK1 PE=1 SV=3	2	1
986	Q9UM54	Unconventional myosin-VI GN=MYO6 PE=1 SV=4	2	1
987	Q9UNH7	Sorting nexin-6 GN=SNX6 PE=1 SV=1	2	1
988	Q8IYJ1	Copine-9 GN=CPNE9 PE=1 SV=3	2	1
989	O00571	ATP-dependent RNA helicase DDX3X GN=DDX3X PE=1 SV=3	2	1
990	Q9BW04	Specifically androgen-regulated gene protein GN=SARG PE=1 SV=2	2	1

991	P07358	Complement component C8 beta chain GN=C8B PE=1 SV=3	2	1
992	Q16629	Serine/arginine-rich splicing factor 7 GN=SRSF7 PE=1 SV=1	2	1
993	P62633	Cellular nucleic acid-binding protein GN=CNBP PE=1 SV=1	2	1
994	P35080	Profilin-2 GN=PFN2 PE=1 SV=3	2	1
995	P18827	Syndecan-1 GN=SDC1 PE=1 SV=3	2	1
996	P07476	Involucrin GN=IVL PE=1 SV=2	2	2
997	P02656	Apolipoprotein C-III GN=APOC3 PE=1 SV=1	2	1
998	O95969	Secretoglobin family 1D member 2 GN=SCGB1D2 PE=2 SV=1	2	1
999	O95274	Ly6/PLAUR domain-containing protein 3 GN=LYPD3 PE=1 SV=2	2	1
1000	O00625	Pirin GN=PIR PE=1 SV=1	2	1
1001	O00204	Sulfotransferase family cytosolic 2B member 1 GN=SULT2B1 PE=1 SV=2	2	2
1002	Q86UD1	Out at first protein homolog GN=OAF PE=2 SV=1	2	1
1003	Q6EEV6	Small ubiquitin-related modifier 4 GN=SUMO4 PE=1 SV=2	2	2
1004	Q15126	Phosphomevalonate kinase GN=PMVK PE=1 SV=3	2	1
1005	P69905	Hemoglobin subunit alpha GN=HBA1 PE=1 SV=2	2	1
1006	P61960	Ubiquitin-fold modifier 1 GN=UFM1 PE=1 SV=1	2	2
1007	P60866	40S ribosomal protein S20 GN=RPS20 PE=1 SV=1	2	1
1008	P46782	40S ribosomal protein S5 GN=RPS5 PE=1 SV=4	2	1
1009	P40855	Peroxisomal biogenesis factor 19 GN=PEX19 PE=1 SV=1	2	1

1010	P14317	Hematopoietic lineage cell-specific protein GN=HCLS1 PE=1 SV=3	2	1
1011	P07910	Heterogeneous nuclear ribonucleoproteins C1/C2 GN=HNRNPC PE=1 SV=4	2	1
1012	Q9NX46	Poly(ADP-ribose) glycohydrolase ARH3 GN=ADPRHL2 PE=1 SV=1	2	1
1013	Q6P996	Pyridoxal-dependent decarboxylase domain-containing protein 1 GN=PDXDC1 PE=1 SV=2	2	1
1014	Q5D862	Filaggrin-2 GN=FLG2 PE=1 SV=1	2	1
1015	Q9Y2T7	Y-box-binding protein 2 GN=YBX2 PE=1 SV=2	2	1
1016	P02746	Complement C1q subcomponent subunit B GN=C1QB PE=1 SV=3	2	1
1017	Q15714	TSC22 domain family protein 1 GN=TSC22D1 PE=1 SV=3	2	1
1018	P51452	Dual specificity protein phosphatase 3 GN=DUSP3 PE=1 SV=1	2	1
1019	P28072	Proteasome subunit beta type-6 GN=PSMB6 PE=1 SV=4	2	1
1020	P62263	40S ribosomal protein S14 GN=RPS14 PE=1 SV=3	2	1
1021	Q07021	Complement component 1 Q subcomponent-binding protein, mitochondrial GN=C1QBP PE=1 SV=1	2	1
1022	P04211	Ig lambda chain V region 4A PE=4 SV=1	2	3
1023	P18124	60S ribosomal protein L7 GN=RPL7 PE=1 SV=1	2	1
1024	P48163	NADP-dependent malic enzyme GN=ME1 PE=1 SV=1	2	1
1025	P21926	CD9 antigen GN=CD9 PE=1 SV=4	2	1
1026	P14735	Insulin-degrading enzyme GN=IDE PE=1 SV=4	2	2
1027	Q9ULC4	Malignant T-cell-amplified sequence 1 GN=MCTS1 PE=1 SV=1	2	1

1028	O95793	Double-stranded RNA-binding protein Staufen homolog 1 GN=STAU1 PE=1 SV=2	2	1
1029	Q8TCB6	Olfactory receptor 51E1 GN=OR51E1 PE=1 SV=1	2	1
1030	P62879	Guanine nucleotide-binding protein G(I)/G(S)/G(T) subunit beta-2 GN=GNB2 PE=1 SV=3	2	1
1031	O00560	Syntenin-1 GN=SDCBP PE=1 SV=1	2	1
1032	P17900	Ganglioside GM2 activator GN=GM2A PE=1 SV=4	2	1
1033	Q66K66	Transmembrane protein 198 GN=TMEM198 PE=1 SV=1	2	2
1034	P04155	Trefoil factor 1 GN=TFF1 PE=1 SV=1	2	1
1035	Q03113	Guanine nucleotide-binding protein subunit alpha-12 GN=GNA12 PE=1 SV=4	2	1
1036	Q99436	Proteasome subunit beta type-7 GN=PSMB7 PE=1 SV=1	2	1
1037	P60903	Protein S100-A10 GN=S100A10 PE=1 SV=2	2	1
1038	Q9Y6X4	Soluble lamin-associated protein of 75 kDa GN=FAM169A PE=1 SV=2	2	2
1039	P18085	ADP-ribosylation factor 4 GN=ARF4 PE=1 SV=3	2	3
1040	P26583	High mobility group protein B2 GN=HMGB2 PE=1 SV=2	2	3
1041	Q9UBL6	Copine-7 GN=CPNE7 PE=2 SV=1	2	1
1042	Q4VXU2	Polyadenylate-binding protein 1-like GN=PABPC1L PE=2 SV=1	2	1
1043	Q9NR46	Endophilin-B2 GN=SH3GLB2 PE=1 SV=1	2	1
1044	Q16864	V-type proton ATPase subunit F GN=ATP6V1F PE=1 SV=2	2	1
1045	P09622	Dihydrolipoyl dehydrogenase, mitochondrial GN=DLD PE=1 SV=2	2	1
1046	P09497	Clathrin light chain B GN=CLTB PE=1 SV=1	2	1

1047	O60812	Heterogeneous nuclear ribonucleoprotein C-like 1 GN=HNRNPCL1 PE=1 SV=1	2	1
1048	O43286	Beta-1,4-galactosyltransferase 5 GN=B4GALT5 PE=2 SV=1	2	1
1049	P62195	26S protease regulatory subunit 8 GN=PSMC5 PE=1 SV=1	2	1
1050	O60927	Protein phosphatase 1 regulatory subunit 11 GN=PPP1R11 PE=1 SV=1	2	1
1051	P61106	Ras-related protein Rab-14 GN=RAB14 PE=1 SV=4	2	2
1052	Q9H0E2	Toll-interacting protein GN=TOLLIP PE=1 SV=1	2	1
1053	P60891	Ribose-phosphate pyrophosphokinase 1 GN=PRPS1 PE=1 SV=2	2	1
1054	P55210	Caspase-7 GN=CASP7 PE=1 SV=1	2	1
1055	P04259	Keratin, type II cytoskeletal 6B GN=KRT6B PE=1 SV=5	2	11
1056	P62913	60S ribosomal protein L11 GN=RPL11 PE=1 SV=2	2	1
1057	P47929	Galectin-7 GN=LGALS7 PE=1 SV=2	2	1
1058	Q07020	60S ribosomal protein L18 GN=RPL18 PE=1 SV=2	2	1
1059	O75531	Barrier-to-autointegration factor GN=BANF1 PE=1 SV=1	2	1
1060	Q9NQ88	Fructose-2,6-bisphosphatase TIGAR GN=TIGAR PE=1 SV=1	2	1
1061	Q13283	Ras GTPase-activating protein-binding protein 1 GN=G3BP1 PE=1 SV=1	2	1
1062	Q9H910	Hematological and neurological expressed 1-like protein GN=HN1L PE=1 SV=1	2	1
1063	Q99963	Endophilin-A3 GN=SH3GL3 PE=1 SV=1	2	3
1064	P53999	Activated RNA polymerase II transcriptional coactivator p15 GN=SUB1 PE=1 SV=3	2	1

1065	Q9Y617	Phosphoserine aminotransferase GN=PSAT1 PE=1 SV=2	2	1
1066	P63165	Small ubiquitin-related modifier 1 GN=SUMO1 PE=1 SV=1	2	1
1067	P31942	Heterogeneous nuclear ribonucleoprotein H3 GN=HNRNPH3 PE=1 SV=2	2	1
1068	Q5JWF2	Guanine nucleotide-binding protein G(s) subunit alpha isoforms XLas GN=GNAS PE=1 SV=2	2	1
1069	Q9Y365	PCTP-like protein GN=STARD10 PE=1 SV=2	2	1
1070	P15289	Arylsulfatase A GN=ARSA PE=1 SV=3	2	1
1071	Q9UKA9	Polypyrimidine tract-binding protein 2 GN=PTBP2 PE=1 SV=1	2	1
1072	Q86V81	THO complex subunit 4 GN=ALYREF PE=1 SV=3	2	1
1073	Q6UXB3	Ly6/PLAUR domain-containing protein 2 GN=LYPD2 PE=2 SV=1	2	1
1074	P62314	Small nuclear ribonucleoprotein Sm D1 GN=SNRPD1 PE=1 SV=1	2	1
1075	O00757	Fructose-1,6-bisphosphatase isozyme 2 GN=FBP2 PE=1 SV=2	2	2
1076	Q9H9H4	Vacuolar protein sorting-associated protein 37B GN=VPS37B PE=1 SV=1	2	1
1077	Q76LX8	A disintegrin and metalloproteinase with thrombospondin motifs 13 GN=ADAMTS13 PE=1 SV=1	2	1
1078	Q86YQ8	Copine-8 GN=CPNE8 PE=1 SV=2	2	1
1079	P27105	Erythrocyte band 7 integral membrane protein GN=STOM PE=1 SV=3	2	1
1080	O43490	Prominin-1 GN=PROM1 PE=1 SV=1	2	1
1081	Q9Y5K8	V-type proton ATPase subunit D GN=ATP6V1D PE=1 SV=1	2	2
1082	Q96M27	Protein PRRC1 GN=PRRC1 PE=1 SV=1	2	1

1083	Q92688	Acidic leucine-rich nuclear phosphoprotein 32 family member B GN=ANP32B PE=1 SV=1	2	1
1084	Q92520	Protein FAM3C GN=FAM3C PE=1 SV=1	2	1
1085	Q15599	Na(+)/H(+) exchange regulatory cofactor NHE-RF2 GN=SLC9A3R2 PE=1 SV=2	2	1
1086	P67870	Casein kinase II subunit beta GN=CSNK2B PE=1 SV=1	2	1
1087	P17096	High mobility group protein HMG-I/HMG-Y GN=HMGA1 PE=1 SV=3	2	1
1088	P62269	40S ribosomal protein S18 GN=RPS18 PE=1 SV=3	2	1
1089	Q15208	Serine/threonine-protein kinase 38 GN=STK38 PE=1 SV=1	2	1
1090	Q6DKI2	Galectin-9C GN=LGALS9C PE=2 SV=2	2	1
1091	Q96MN9	Zinc finger protein 488 GN=ZNF488 PE=1 SV=1	2	1
1092	P47712	Cytosolic phospholipase A2 GN=PLA2G4A PE=1 SV=2	2	1
1093	Q9NUU7	ATP-dependent RNA helicase DDX19A GN=DDX19A PE=1 SV=1	2	1
1094	P50452	Serpin B8 GN=SERPINB8 PE=1 SV=2	2	4
1095	Q99627	COP9 signalosome complex subunit 8 GN=COPS8 PE=1 SV=1	2	1
1096	O75828	Carbonyl reductase [NADPH] 3 GN=CBR3 PE=1 SV=3	2	4
1097	Q14166	Tubulin--tyrosine ligase-like protein 12 GN=TTLL12 PE=1 SV=2	2	1
1098	P01701	Ig lambda chain V-I region NEW PE=1 SV=1	2	1
1099	B2RXH8	Heterogeneous nuclear ribonucleoprotein C-like 2 GN=HNRNPCL2 PE=2 SV=1	2	1
1100	Q5TBC7	Bcl-2-like protein 15 GN=BCL2L15 PE=2 SV=1	2	1
1101	P12268	Inosine-5'-monophosphate dehydrogenase 2	2	1

		GN=IMPDH2 PE=1 SV=2		
1102	Q9UBQ5	Eukaryotic translation initiation factor 3 subunit K GN=EIF3K PE=1 SV=1	2	1
1103	O00170	AH receptor-interacting protein GN=AIP PE=1 SV=2	2	1
1104	Q9NQP4	Prefoldin subunit 4 GN=PFDN4 PE=1 SV=1	2	1
1105	P01603	Ig kappa chain V-I region Ka PE=1 SV=1	2	2
1106	P61247	40S ribosomal protein S3a GN=RPS3A PE=1 SV=2	2	1
1107	P36969	Phospholipid hydroperoxide glutathione peroxidase, mitochondrial GN=GPX4 PE=1 SV=3	2	1
1108	P07360	Complement component C8 gamma chain GN=C8G PE=1 SV=3	2	1
1109	P27487	Dipeptidyl peptidase 4 GN=DPP4 PE=1 SV=2	2	1
1110	P16989	Y-box-binding protein 3 GN=YBX3 PE=1 SV=4	2	1
1111	Q8TBX8	Phosphatidylinositol 5-phosphate 4-kinase type-2 gamma GN=PIP4K2C PE=1 SV=3	2	1
1112	Q96AE4	Far upstream element-binding protein 1 GN=FUBP1 PE=1 SV=3	2	1
1113	Q6PIW4	Fidgetin-like protein 1 GN=FIGNL1 PE=1 SV=2	2	2
1114	Q7L1Q6	Basic leucine zipper and W2 domain-containing protein 1 GN=BZW1 PE=1 SV=1	2	1
1115	Q71UI9	Histone H2A.V GN=H2AFV PE=1 SV=3	2	1
1116	Q16774	Guanylate kinase GN=GUK1 PE=1 SV=2	2	1
1117	P61956	Small ubiquitin-related modifier 2 GN=SUMO2 PE=1 SV=3	2	2
1118	Q6IA69	Glutamine-dependent NAD(+) synthetase GN=NADSYN1 PE=1 SV=3	2	1
1119	Q03169	Tumor necrosis factor alpha-induced protein 2 GN=TNFAIP2 PE=1 SV=2	2	1

1120	P14866	Heterogeneous nuclear ribonucleoprotein L GN=HNRNPL PE=1 SV=2	2	1
1121	Q99829	Copine-1 GN=CPNE1 PE=1 SV=1	2	2
1122	Q92890	Ubiquitin fusion degradation protein 1 homolog GN=UFD1L PE=1 SV=3	2	1
1123	O75340	Programmed cell death protein 6 GN=PDCD6 PE=1 SV=1	2	1
1124	Q92499	ATP-dependent RNA helicase DDX1 GN=DDX1 PE=1 SV=2	2	1
1125	O60282	Kinesin heavy chain isoform 5C GN=KIF5C PE=1 SV=1	2	1
1126	Q9UN76	Sodium- and chloride-dependent neutral and basic amino acid transporter B(0+) GN=SLC6A14 PE=2 SV=1	2	1
1127	Q8IYD1	Eukaryotic peptide chain release factor GTP-binding subunit ERF3B GN=GSPT2 PE=1 SV=2	2	1
1128	Q29963	HLA class I histocompatibility antigen, Cw-6 alpha chain GN=HLA-C PE=2 SV=2	2	1
1129	P49407	Beta-arrestin-1 GN=ARRB1 PE=1 SV=2	2	1
1130	Q9BY43	Charged multivesicular body protein 4a GN=CHMP4A PE=1 SV=3	2	1
1131	Q9UQ16	Dynamin-3 GN=DNM3 PE=1 SV=4	2	1
1132	Q9Y259	Choline/ethanolamine kinase GN=CHKB PE=1 SV=3	2	1
1133	O75822	Eukaryotic translation initiation factor 3 subunit J GN=EIF3J PE=1 SV=2	2	1
1134	Q96AT9	Ribulose-phosphate 3-epimerase GN=RPE PE=1 SV=1	2	1
1135	P41218	Myeloid cell nuclear differentiation antigen GN=MNDA PE=1 SV=1	2	1
1136	P01609	Ig kappa chain V-I region Scw PE=1 SV=1	2	14
1137	Q8IWP9	Coiled-coil domain-containing protein 28A GN=CCDC28A PE=2 SV=1	2	1

1138	P0CG04	Ig lambda-1 chain C regions GN=IGLC1 PE=1 SV=1	2	58
1139	Q9UHV9	Prefoldin subunit 2 GN=PFDN2 PE=1 SV=1	2	1
1140	Q9H7C9	Mth938 domain-containing protein GN=AAMDC PE=1 SV=1	2	1
1141	Q9H115	Beta-soluble NSF attachment protein GN=NAPB PE=1 SV=2	2	1
1142	Q9BUL8	Programmed cell death protein 10 GN=PDCD10 PE=1 SV=1	2	1
1143	P57764	Gasdermin-D GN=GSDMD PE=1 SV=1	2	1
1144	P10155	60 kDa SS-A/Ro ribonucleoprotein GN=TROVE2 PE=1 SV=2	2	1
1145	Q13347	Eukaryotic translation initiation factor 3 subunit I GN=EIF3I PE=1 SV=1	2	1
1146	P49441	Inositol polyphosphate 1-phosphatase GN=INPP1 PE=1 SV=1	2	1
1147	Q6P1N9	Putative deoxyribonuclease TATDN1 GN=TATDN1 PE=1 SV=2	2	1
1148	Q15370	Transcription elongation factor B polypeptide 2 GN=TCEB2 PE=1 SV=1	2	1
1149	Q9UBW8	COP9 signalosome complex subunit 7a GN=COPS7A PE=1 SV=1	2	1
1150	Q8WUF5	RelA-associated inhibitor GN=PPP1R13L PE=1 SV=4	2	1
1151	Q16401	26S proteasome non-ATPase regulatory subunit 5 GN=PSMD5 PE=1 SV=3	2	1
1152	Q8NDH3	Probable aminopeptidase NPEPL1 GN=NPEPL1 PE=1 SV=3	2	1
1153	P23141	Liver carboxylesterase 1 GN=CES1 PE=1 SV=2	2	1
1154	P15291	Beta-1,4-galactosyltransferase 1 GN=B4GALT1 PE=1 SV=5	2	1
1155	P02649	Apolipoprotein E GN=APOE PE=1 SV=1	2	1

1156	P54619	5'-AMP-activated protein kinase subunit gamma-1 GN=PRKAG1 PE=1 SV=1	2	1
1157	P36507	Dual specificity mitogen-activated protein kinase kinase 2 GN=MAP2K2 PE=1 SV=1	2	1
1158	O60635	Tetraspanin-1 GN=TSPAN1 PE=1 SV=2	2	1
1159	P46783	40S ribosomal protein S10 GN=RPS10 PE=1 SV=1	2	1
1160	P00748	Coagulation factor XII GN=F12 PE=1 SV=3	2	1
1161	P23246	Splicing factor, proline- and glutamine-rich GN=SFPQ PE=1 SV=2	2	1
1162	P20042	Eukaryotic translation initiation factor 2 subunit 2 GN=EIF2S2 PE=1 SV=2	2	1
1163	P16083	Ribosyldihydronicotinamide dehydrogenase [quinone] GN=NQO2 PE=1 SV=5	2	1
1164	O95379	Tumor necrosis factor alpha-induced protein 8 GN=TNFAIP8 PE=1 SV=1	2	1
1165	Q9NQX3	Gephyrin GN=GPHN PE=1 SV=1	2	1
1166	P08133	Annexin A6 GN=ANXA6 PE=1 SV=3	2	2
1167	Q9BXR6	Complement factor H-related protein 5 GN=CFHR5 PE=1 SV=1	2	1
1168	P30049	ATP synthase subunit delta, mitochondrial GN=ATP5D PE=1 SV=2	2	1
1169	Q6PCE3	Glucose 1,6-bisphosphate synthase GN=PGM2L1 PE=1 SV=3	2	1
1170	Q92804	TATA-binding protein-associated factor 2N GN=TAF15 PE=1 SV=1	2	1
1171	O00399	Dynactin subunit 6 GN=DCTN6 PE=1 SV=1	2	1
1172	P50552	Vasodilator-stimulated phosphoprotein GN=VASP PE=1 SV=3	2	1
1173	O14933	Ubiquitin/ISG15-conjugating enzyme E2 L6 GN=UBE2L6 PE=1 SV=4	2	1
1174	Q12904	Aminoacyl tRNA synthase complex-interacting multifunctional protein 1 GN=AIMP1 PE=1	2	1

		SV=2		
1175	Q9UBX1	Cathepsin F GN=CTSF PE=1 SV=1	2	1
1176	Q9BUH6	Uncharacterized protein C9orf142 GN=C9orf142 PE=1 SV=2	2	1
1177	Q96A05	V-type proton ATPase subunit E 2 GN=ATP6V1E2 PE=2 SV=1	2	1
1178	Q96BW5	Phosphotriesterase-related protein GN=PTER PE=1 SV=1	2	1
1179	Q6P6B1	Glutamate-rich protein 5 GN=ERICH5 PE=2 SV=1	2	1
1180	Q9P218	Collagen alpha-1(XX) chain GN=COL20A1 PE=1 SV=4	2	1
1181	Q96QV6	Histone H2A type 1-A GN=HIST1H2AA PE=1 SV=3	2	1
1182	Q96P70	Importin-9 GN=IPO9 PE=1 SV=3	2	1
1183	P08582	Melanotransferrin GN=MFI2 PE=1 SV=2	2	2
1184	Q15942	Zyxin GN=ZYX PE=1 SV=1	2	1
1185	P62241	40S ribosomal protein S8 GN=RPS8 PE=1 SV=2	2	2
1186	Q01459	Di-N-acetylchitobiase GN=CTBS PE=1 SV=1	2	1
1187	Q8NFJ5	Retinoic acid-induced protein 3 GN=GPRC5A PE=1 SV=2	2	1
1188	Q14195	Dihydropyrimidinase-related protein 3 GN=DPYSL3 PE=1 SV=1	2	1
1189	P98179	Putative RNA-binding protein 3 GN=RBM3 PE=1 SV=1	2	1
1190	O94903	Proline synthase co-transcribed bacterial homolog protein GN=PROSC PE=1 SV=1	2	1
1191	O00233	26S proteasome non-ATPase regulatory subunit 9 GN=PSMD9 PE=1 SV=3	2	1
1192	Q01082	Spectrin beta chain, non-erythrocytic 1 GN=SPTBN1 PE=1 SV=2	2	1

1193	Q15437	Protein transport protein Sec23B GN=SEC23B PE=1 SV=2	2	1
1194	Q9UMX5	Neudesin GN=NENF PE=1 SV=1	2	1
1195	P36578	60S ribosomal protein L4 GN=RPL4 PE=1 SV=5	2	1
1196	O43633	Charged multivesicular body protein 2a GN=CHMP2A PE=1 SV=1	2	1
1197	Q9H3U1	Protein unc-45 homolog A GN=UNC45A PE=1 SV=1	2	1
1198	P15498	Proto-oncogene vav GN=VAV1 PE=1 SV=4	2	2
1199	P07357	Complement component C8 alpha chain GN=C8A PE=1 SV=2	2	1
1200	Q9H3S4	Thiamin pyrophosphokinase 1 GN=TPK1 PE=1 SV=1	2	1
1201	Q08211	ATP-dependent RNA helicase A GN=DHX9 PE=1 SV=4	2	1
1202	P62993	Growth factor receptor-bound protein 2 GN=GRB2 PE=1 SV=1	2	1
1203	Q96CV9	Optineurin GN=OPTN PE=1 SV=2	2	1
1204	P01778	Ig heavy chain V-III region ZAP PE=1 SV=1	2	4
1205	O60701	UDP-glucose 6-dehydrogenase GN=UGDH PE=1 SV=1	2	1
1206	Q9Y5Y9	Sodium channel protein type 10 subunit alpha GN=SCN10A PE=1 SV=2	2	1
1207	Q9Y6W5	Wiskott-Aldrich syndrome protein family member 2 GN=WASF2 PE=1 SV=3	2	1
1208	P38646	Stress-70 protein, mitochondrial GN=HSPA9 PE=1 SV=2	2	1
1209	O75396	Vesicle-trafficking protein SEC22b GN=SEC22B PE=1 SV=4	2	1
1210	P35244	Replication protein A 14 kDa subunit GN=RPA3 PE=1 SV=1	2	1
1211	P30566	Adenylosuccinate lyase GN=ADSL PE=1	2	1

		SV=2		
1212	A5D8V6	Vacuolar protein sorting-associated protein 37C GN=VPS37C PE=1 SV=2	2	1
1213	O00232	26S proteasome non-ATPase regulatory subunit 12 GN=PSMD12 PE=1 SV=3	2	1
1214	Q9BVA1	Tubulin beta-2B chain GN=TUBB2B PE=1 SV=1	2	15
1215	P01593	Ig kappa chain V-I region AG PE=1 SV=1	2	13
1216	Q7KZF4	Staphylococcal nuclease domain-containing protein 1 GN=SND1 PE=1 SV=1	2	1
1217	P55854	Small ubiquitin-related modifier 3 GN=SUMO3 PE=1 SV=2	2	1
1218	Q9C0C2	182 kDa tankyrase-1-binding protein GN=TNKS1BP1 PE=1 SV=4	2	1
1219	Q9NZZ3	Charged multivesicular body protein 5 GN=CHMP5 PE=1 SV=1	2	1
1220	Q9Y6R7	IgGFc-binding protein GN=FCGBP PE=1 SV=3	2	1
1221	O43653	Prostate stem cell antigen GN=PSCA PE=1 SV=1	2	1
1222	Q8WXX0	Dynein heavy chain 7, axonemal GN=DNAH7 PE=1 SV=2	2	1
1223	O95886	Disks large-associated protein 3 GN=DLGAP3 PE=1 SV=3	2	1
1224	Q8N7X1	RNA-binding motif protein, X-linked-like-3 GN=RBMXL3 PE=2 SV=2	2	1
1225	Q9Y2W2	WW domain-binding protein 11 GN=WBP11 PE=1 SV=1	2	1
1226	P53990	IST1 homolog GN=IST1 PE=1 SV=1	2	1
1227	Q9Y376	Calcium-binding protein 39 GN=CAB39 PE=1 SV=1	2	1
1228	P01596	Ig kappa chain V-I region CAR PE=1 SV=1	2	5
1229	Q9Y3E7	Charged multivesicular body protein 3	2	1

		GN=CHMP3 PE=1 SV=3		
1230	Q06033	Inter-alpha-trypsin inhibitor heavy chain H3 GN=ITIH3 PE=1 SV=2	2	1
1231	P02792	Ferritin light chain GN=FTL PE=1 SV=2	2	1
1232	Q04446	1,4-alpha-glucan-branching enzyme GN=GBE1 PE=1 SV=3	2	1
1233	P61201	COP9 signalosome complex subunit 2 GN=COPS2 PE=1 SV=1	2	1
1234	P46777	60S ribosomal protein L5 GN=RPL5 PE=1 SV=3	2	1
1235	Q5VWZ2	Lysophospholipase-like protein 1 GN=LYPLAL1 PE=1 SV=3	2	1
1236	P62249	40S ribosomal protein S16 GN=RPS16 PE=1 SV=2	2	1
1237	Q9UJC5	SH3 domain-binding glutamic acid-rich-like protein 2 GN=SH3BGRL2 PE=1 SV=2	2	1
1238	Q9H8Y8	Golgi reassembly-stacking protein 2 GN=GORASP2 PE=1 SV=3	2	1
1239	Q16836	Hydroxyacyl-coenzyme A dehydrogenase, mitochondrial GN=HADH PE=1 SV=3	2	1
1240	P50226	Sulfotransferase 1A2 GN=SULT1A2 PE=1 SV=2	2	1
1241	P01624	Ig kappa chain V-III region POM PE=1 SV=1	2	3
1242	Q92696	Geranylgeranyl transferase type-2 subunit alpha GN=RABGGTA PE=1 SV=2	2	1
1243	P62829	60S ribosomal protein L23 GN=RPL23 PE=1 SV=1	2	1
1244	P61966	AP-1 complex subunit sigma-1A GN=AP1S1 PE=1 SV=1	2	1
1245	P12532	Creatine kinase U-type, mitochondrial GN=CKMT1A PE=1 SV=1	2	1
1246	P61923	Coatomer subunit zeta-1 GN=COPZ1 PE=1 SV=1	2	2

1247	O75506	Heat shock factor-binding protein 1 GN=HSBP1 PE=1 SV=1	2	1
1248	Q8WXC6	Myeloma-overexpressed gene 2 protein GN=MYEOV2 PE=2 SV=3	2	1
1249	Q9UHB6	LIM domain and actin-binding protein 1 GN=LIMA1 PE=1 SV=1	2	1
1250	P04899	Guanine nucleotide-binding protein G(i) subunit alpha-2 GN=GNAI2 PE=1 SV=3	2	1
1251	Q9Y5K6	CD2-associated protein GN=CD2AP PE=1 SV=1	2	1
1252	Q96C90	Protein phosphatase 1 regulatory subunit 14B GN=PPP1R14B PE=1 SV=3	2	1
1253	Q9HCH3	Copine-5 GN=CPNE5 PE=1 SV=2	2	1
1254	Q9P0R6	GSK3-beta interaction protein GN=GSKIP PE=1 SV=2	2	1
1255	Q9H300	Presenilins-associated rhomboid-like protein, mitochondrial GN=PARL PE=1 SV=2	2	1
1256	Q8WTQ4	Uncharacterized protein C16orf78 GN=C16orf78 PE=2 SV=1	2	1
1257	Q8N0W5	IQ domain-containing protein K GN=IQCK PE=2 SV=1	2	1
1258	P57082	T-box transcription factor TBX4 GN=TBX4 PE=1 SV=2	2	1
1259	O95427	GPI ethanolamine phosphate transferase 1 GN=PIGN PE=1 SV=1	2	1
1260	P50570	Dynamin-2 GN=DNM2 PE=1 SV=2	2	2
1261	O00442	RNA 3'-terminal phosphate cyclase GN=RTCA PE=1 SV=1	2	1
1262	Q9BQE3	Tubulin alpha-1C chain GN=TUBA1C PE=1 SV=1	2	13
1263	P16401	Histone H1.5 GN=HIST1H1B PE=1 SV=3	2	1
1264	Q9UKS7	Zinc finger protein Helios GN=IKZF2 PE=1 SV=2	2	1

1265	O60925	Prefoldin subunit 1 GN=PFDN1 PE=1 SV=2	2	1
1266	P25685	DnaJ homolog subfamily B member 1 GN=DNAJB1 PE=1 SV=4	1.89	1
1267	Q9P289	Serine/threonine-protein kinase MST4 GN=MST4 PE=1 SV=2	1.89	2
1268	Q9Y2Z0	Suppressor of G2 allele of SKP1 homolog GN=SUGT1 PE=1 SV=3	1.89	1
1269	P57053	Histone H2B type F-S GN=H2BFS PE=1 SV=2	1.89	2
1270	P0DMN0	Sulfotransferase 1A4 GN=SULT1A4 PE=1 SV=1	1.89	2
1271	O00462	Beta-mannosidase GN=MANBA PE=1 SV=3	1.89	1
1272	Q92747	Actin-related protein 2/3 complex subunit 1A GN=ARPC1A PE=1 SV=2	1.82	2
1273	Q9Y2B0	Protein canopy homolog 2 GN=CNPY2 PE=1 SV=1	1.8	1
1274	P04438	Ig heavy chain V-II region SESS PE=2 SV=1	1.8	1
1275	P43251	Biotinidase GN=BTD PE=1 SV=2	1.78	1
1276	P08754	Guanine nucleotide-binding protein G(k) subunit alpha GN=GNAI3 PE=1 SV=3	1.77	2
1277	Q9C0G6	Dynein heavy chain 6, axonemal GN=DNAH6 PE=1 SV=3	1.77	1
1278	P35241	Radixin GN=RDX PE=1 SV=1	1.72	11
1279	Q8WZA6	Olfactory receptor 1E3 GN=OR1E3 PE=3 SV=2	1.72	1
1280	Q9UHA4	Ragulator complex protein LAMTOR3 GN=LAMTOR3 PE=1 SV=1	1.72	1
1281	O43390	Heterogeneous nuclear ribonucleoprotein R GN=HNRNPR PE=1 SV=1	1.7	2
1282	Q687X5	Metalloreductase STEAP4 GN=STEAP4 PE=1 SV=1	1.7	1
1283	Q9NP99	Triggering receptor expressed on myeloid cells 1 GN=TREM1 PE=1 SV=1	1.7	1

1284	O95721	Synaptosomal-associated protein 29 GN=SNAP29 PE=1 SV=1	1.68	1
1285	P99999	Cytochrome c GN=CYCS PE=1 SV=2	1.68	1
1286	Q96BQ1	Protein FAM3D GN=FAM3D PE=1 SV=1	1.68	1
1287	P01824	Ig heavy chain V-II region WAH PE=1 SV=1	1.68	2
1288	Q04609	Glutamate carboxypeptidase 2 GN=FOLH1 PE=1 SV=1	1.68	1
1289	Q13409	Cytoplasmic dynein 1 intermediate chain 2 GN=DYNC1I2 PE=1 SV=3	1.66	2
1290	Q9Y333	U6 snRNA-associated Sm-like protein LSm2 GN=LSM2 PE=1 SV=1	1.66	1
1291	P62424	60S ribosomal protein L7a GN=RPL7A PE=1 SV=2	1.66	1
1292	P11908	Ribose-phosphate pyrophosphokinase 2 GN=PRPS2 PE=1 SV=2	1.66	1
1293	Q00266	S-adenosylmethionine synthase isoform type-1 GN=MAT1A PE=1 SV=2	1.66	1
1294	Q13287	N-myc-interactor GN=NMI PE=1 SV=2	1.64	1
1295	Q03013	Glutathione S-transferase Mu 4 GN=GSTM4 PE=1 SV=3	1.64	1
1296	O00629	Importin subunit alpha-3 GN=KPNA4 PE=1 SV=1	1.62	1
1297	P05067	Amyloid beta A4 protein GN=APP PE=1 SV=3	1.62	1
1298	Q5SGD2	Protein phosphatase 1L GN=PPM1L PE=1 SV=1	1.62	1
1299	P09237	Matrilysin GN=MMP7 PE=1 SV=1	1.6	1
1300	Q9BRG1	Vacuolar protein-sorting-associated protein 25 GN=VPS25 PE=1 SV=1	1.6	1
1301	Q03154	Aminoacylase-1 GN=ACY1 PE=1 SV=1	1.59	1
1302	Q9UI12	V-type proton ATPase subunit H GN=ATP6V1H PE=1 SV=1	1.59	1
1303	Q969R2	Oxysterol-binding protein 2 GN=OSBP2 PE=1	1.59	1

		SV=2		
1304	P55010	Eukaryotic translation initiation factor 5 GN=EIF5 PE=1 SV=2	1.57	1
1305	Q76FK4	Nucleolar protein 8 GN=NOL8 PE=1 SV=1	1.57	1
1306	Q969T9	WW domain-binding protein 2 GN=WBP2 PE=1 SV=1	1.55	1
1307	Q92623	Tetratricopeptide repeat protein 9A GN=TTC9 PE=2 SV=3	1.54	1
1308	P47914	60S ribosomal protein L29 GN=RPL29 PE=1 SV=2	1.52	1
1309	P34913	Bifunctional epoxide hydrolase 2 GN=EPHX2 PE=1 SV=2	1.52	2
1310	Q9UK41	Vacuolar protein sorting-associated protein 28 homolog GN=VPS28 PE=1 SV=1	1.51	1
1311	Q12906	Interleukin enhancer-binding factor 3 GN=ILF3 PE=1 SV=3	1.49	1
1312	Q9UBN7	Histone deacetylase 6 GN=HDAC6 PE=1 SV=2	1.49	1
1313	P48595	Serpin B10 GN=SERPINB10 PE=1 SV=1	1.47	1
1314	P35268	60S ribosomal protein L22 GN=RPL22 PE=1 SV=2	1.47	1
1315	Q7Z478	ATP-dependent RNA helicase DHX29 GN=DHX29 PE=1 SV=2	1.47	1
1316	Q9UBR2	Cathepsin Z GN=CTSZ PE=1 SV=1	1.46	1
1317	Q8TED9	Actin filament-associated protein 1-like 1 GN=AFAP1L1 PE=1 SV=2	1.44	1
1318	Q8NBJ7	Sulfatase-modifying factor 2 GN=SUMF2 PE=1 SV=2	1.43	1
1319	O43237	Cytoplasmic dynein 1 light intermediate chain 2 GN=DYNC1LI2 PE=1 SV=1	1.43	1
1320	A0MZ66	Shootin-1 GN=KIAA1598 PE=1 SV=4	1.42	1
1321	Q9NR28	Diablo homolog, mitochondrial GN=DIABLO PE=1 SV=1	1.41	1

1322	Q99729	Heterogeneous nuclear ribonucleoprotein A/B GN=HNRNPAB PE=1 SV=2	1.4	2
1323	Q99519	Sialidase-1 GN=NEU1 PE=1 SV=1	1.39	1
1324	P01608	Ig kappa chain V-I region Roy PE=1 SV=1	1.37	15
1325	Q8WVQ1	Soluble calcium-activated nucleotidase 1 GN=CANT1 PE=1 SV=1	1.36	1
1326	P06731	Carcinoembryonic antigen-related cell adhesion molecule 5 GN=CEACAM5 PE=1 SV=3	1.35	1
1327	Q13596	Sorting nexin-1 GN=SNX1 PE=1 SV=3	1.34	1
1328	O95479	GDH/6PGL endoplasmic bifunctional protein GN=H6PD PE=1 SV=2	1.34	1
1329	O15247	Chloride intracellular channel protein 2 GN=CLIC2 PE=1 SV=3	1.34	1
1330	O95671	N-acetylserotonin O-methyltransferase-like protein GN=ASMTL PE=1 SV=3	1.33	1
1331	Q9Y5B9	FACT complex subunit SPT16 GN=SUPT16H PE=1 SV=1	1.33	1
1332	Q6MZZ7	Calpain-13 GN=CAPN13 PE=1 SV=2	1.32	1
1333	Q5VZK9	Leucine-rich repeat-containing protein 16A GN=LRRC16A PE=1 SV=1	1.31	1
1334	P35542	Serum amyloid A-4 protein GN=SAA4 PE=1 SV=2	1.3	1
1335	Q00610	Clathrin heavy chain 1 GN=CLTC PE=1 SV=5	1.28	1
1336	P15328	Folate receptor alpha GN=FOLR1 PE=1 SV=3	1.28	1
1337	O00194	Ras-related protein Rab-27B GN=RAB27B PE=1 SV=4	1.27	2
1338	O00182	Galectin-9 GN=LGALS9 PE=1 SV=2	1.27	1
1339	Q11128	Alpha-(1,3)-fucosyltransferase 5 GN=FUT5 PE=2 SV=1	1.27	1
1340	P22894	Neutrophil collagenase GN=MMP8 PE=1 SV=1	1.25	1

1341	Q15582	Transforming growth factor-beta-induced protein ig-h3 GN=TGFBI PE=1 SV=1	1.23	1
1342	Q9UKE5	TRAF2 and NCK-interacting protein kinase GN=TNIK PE=1 SV=1	1.23	1
1343	P15531	Nucleoside diphosphate kinase A GN=NME1 PE=1 SV=1	1.22	10
1344	P16070	CD44 antigen GN=CD44 PE=1 SV=3	1.22	1
1345	Q15369	Transcription elongation factor B polypeptide 1 GN=TCEB1 PE=1 SV=1	1.22	1
1346	Q8NEY1	Neuron navigator 1 GN=NAV1 PE=1 SV=2	1.22	1
1347	P14324	Farnesyl pyrophosphate synthase GN=FDPS PE=1 SV=4	1.19	1
1348	Q15102	Platelet-activating factor acetylhydrolase IB subunit gamma GN=PAFAH1B3 PE=1 SV=1	1.19	3
1349	Q16186	Proteasomal ubiquitin receptor ADRM1 GN=ADRM1 PE=1 SV=2	1.19	2
1350	P10253	Lysosomal alpha-glucosidase GN=GAA PE=1 SV=4	1.17	1
1351	P57735	Ras-related protein Rab-25 GN=RAB25 PE=1 SV=2	1.16	1
1352	Q9BUP3	Oxidoreductase HTATIP2 GN=HTATIP2 PE=1 SV=2	1.15	1
1353	P58876	Histone H2B type 1-D GN=HIST1H2BD PE=1 SV=2	1.14	1
1354	P54802	Alpha-N-acetylglucosaminidase GN=NAGLU PE=1 SV=2	1.13	1
1355	P17050	Alpha-N-acetylgalactosaminidase GN=NAGA PE=1 SV=2	1.12	1
1356	Q16589	Cyclin-G2 GN=CCNG2 PE=2 SV=1	1.11	2
1357	Q86SF2	N-acetylgalactosaminyltransferase 7 GN=GALNT7 PE=1 SV=1	1.1	1
1358	O95741	Copine-6 GN=CPNE6 PE=1 SV=3	1.09	1
1359	P53680	AP-2 complex subunit sigma GN=AP2S1	1.08	1

		PE=1 SV=2		
1360	Q14204	Cytoplasmic dynein 1 heavy chain 1 GN=DYNC1H1 PE=1 SV=5	1.08	1
1361	P48739	Phosphatidylinositol transfer protein beta isoform GN=PITPNB PE=1 SV=2	1.08	1
1362	Q12905	Interleukin enhancer-binding factor 2 GN=ILF2 PE=1 SV=2	1.08	1
1363	Q14134	Tripartite motif-containing protein 29 GN=TRIM29 PE=1 SV=2	1.07	1
1364	Q9C002	Normal mucosa of esophagus-specific gene 1 protein GN=NMES1 PE=2 SV=1	1.04	1
1365	Q9Y5X3	Sorting nexin-5 GN=SNX5 PE=1 SV=1	1.04	1
1366	Q9Y3I0	tRNA-splicing ligase RtcB homolog GN=RTCB PE=1 SV=1	1.03	1
1367	P10643	Complement component C7 GN=C7 PE=1 SV=2	1.01	1
1368	Q8IVS8	Glycerate kinase GN=GLYCTK PE=1 SV=1	0.98	1
1369	Q9BV57	1,2-dihydroxy-3-keto-5-methylthiopentene dioxygenase GN=ADI1 PE=1 SV=1	0.97	1
1370	P16930	Fumarylacetoacetase GN=FAH PE=1 SV=2	0.97	1
1371	Q8NH57	Putative olfactory receptor 52P1 GN=OR52P1P PE=5 SV=2	0.97	1
1372	Q9BY12	S phase cyclin A-associated protein in the endoplasmic reticulum GN=SCAPER PE=1 SV=2	0.94	1
1373	P62851	40S ribosomal protein S25 GN=RPS25 PE=1 SV=1	0.94	1
1374	P42892	Endothelin-converting enzyme 1 GN=ECE1 PE=1 SV=2	0.92	1
1375	O43747	AP-1 complex subunit gamma-1 GN=AP1G1 PE=1 SV=5	0.91	1
1376	Q9H2H8	Peptidyl-prolyl cis-trans isomerase-like 3 GN=PPIL3 PE=1 SV=1	0.91	1

1377	Q16576	Histone-binding protein RBBP7 GN=RBBP7 PE=1 SV=1	0.88	1
1378	Q92597	Protein NDRG1 GN=NDRG1 PE=1 SV=1	0.87	1
1379	Q9NVS9	Pyridoxine-5'-phosphate oxidase GN=PNPO PE=1 SV=1	0.86	1
1380	Q13685	Angio-associated migratory cell protein GN=AAMP PE=1 SV=2	0.86	1
1381	Q14746	Conserved oligomeric Golgi complex subunit 2 GN=COG2 PE=1 SV=1	0.86	1
1382	Q99961	Endophilin-A2 GN=SH3GL1 PE=1 SV=1	0.85	1
1383	P10619	Lysosomal protective protein GN=CTSA PE=1 SV=2	0.85	1
1384	P01708	Ig lambda chain V-II region BUR PE=1 SV=1	0.82	2
1385	Q7Z6B7	SLIT-ROBO Rho GTPase-activating protein 1 GN=SRGAP1 PE=1 SV=1	0.81	1
1386	Q9BRT3	Migration and invasion enhancer 1 GN=MIEN1 PE=1 SV=1	0.81	1
1387	Q96RF0	Sorting nexin-18 GN=SNX18 PE=1 SV=2	0.81	1
1388	P58499	Protein FAM3B GN=FAM3B PE=1 SV=2	0.81	1
1389	P22692	Insulin-like growth factor-binding protein 4 GN=IGFBP4 PE=1 SV=2	0.8	1
1390	P16104	Histone H2AX GN=H2AFX PE=1 SV=2	0.79	1
1391	Q13438	Protein OS-9 GN=OS9 PE=1 SV=1	0.79	1
1392	P01615	Ig kappa chain V-II region FR PE=1 SV=1	0.78	3
1393	Q9UBS4	DnaJ homolog subfamily B member 11 GN=DNAJB11 PE=1 SV=1	0.77	1
1394	P09382	Galectin-1 GN=LGALS1 PE=1 SV=2	0.76	1
1395	Q8N9B5	Junction-mediating and -regulatory protein GN=JMY PE=1 SV=2	0.76	1
1396	P40429	60S ribosomal protein L13a GN=RPL13A PE=1 SV=2	0.75	1

1397	P42704	Leucine-rich PPR motif-containing protein, mitochondrial GN=LRPPRC PE=1 SV=3	0.75	1
1398	O14841	5-oxoprolinase GN=OPLAH PE=1 SV=3	0.75	1
1399	Q10570	Cleavage and polyadenylation specificity factor subunit 1 GN=CPSF1 PE=1 SV=2	0.75	1
1400	Q9Y2J4	Angiomotin-like protein 2 GN=AMOTL2 PE=1 SV=3	0.74	1
1401	O95352	Ubiquitin-like modifier-activating enzyme ATG7 GN=ATG7 PE=1 SV=1	0.74	1
1402	O95573	Long-chain-fatty-acid--CoA ligase 3 GN=ACSL3 PE=1 SV=3	0.73	1
1403	P38405	Guanine nucleotide-binding protein G(olf) subunit alpha GN=GNAL PE=1 SV=1	0.72	1
1404	Q8TDN4	CDK5 and ABL1 enzyme substrate 1 GN=CABLES1 PE=1 SV=2	0.72	1
1405	A6NCS6	Uncharacterized protein C2orf72 GN=C2orf72 PE=2 SV=2	0.71	1
1406	P30460	HLA class I histocompatibility antigen, B-8 alpha chain GN=HLA-B PE=1 SV=1	0.7	1
1407	O95819	Mitogen-activated protein kinase kinase kinase kinase 4 GN=MAP4K4 PE=1 SV=2	0.7	1
1408	Q96QB1	Rho GTPase-activating protein 7 GN=DLC1 PE=1 SV=4	0.69	1
1409	Q9BVJ7	Dual specificity protein phosphatase 23 GN=DUSP23 PE=1 SV=1	0.68	1
1410	Q9NZD2	Glycolipid transfer protein GN=GLTP PE=1 SV=3	0.66	1
1411	Q04637	Eukaryotic translation initiation factor 4 gamma 1 GN=EIF4G1 PE=1 SV=4	0.66	1
1412	Q9UMY4	Sorting nexin-12 GN=SNX12 PE=1 SV=3	0.66	1
1413	Q99613	Eukaryotic translation initiation factor 3 subunit C GN=EIF3C PE=1 SV=1	0.66	1
1414	P38432	Coilin GN=COIL PE=1 SV=1	0.64	1

1415	Q7L014	Probable ATP-dependent RNA helicase DDX46 GN=DDX46 PE=1 SV=2	0.64	1
1416	Q674X7	Kazrin GN=KAZN PE=1 SV=2	0.64	1
1417	P51991	Heterogeneous nuclear ribonucleoprotein A3 GN=HNRNPA3 PE=1 SV=2	0.63	2
1418	P01815	Ig heavy chain V-II region COR PE=1 SV=1	0.62	1
1419	P20336	Ras-related protein Rab-3A GN=RAB3A PE=1 SV=1	0.61	2
1420	Q14533	Keratin, type II cuticular Hb1 GN=KRT81 PE=1 SV=3	0.61	1
1421	Q15562	Transcriptional enhancer factor TEF-4 GN=TEAD2 PE=1 SV=2	0.61	1
1422	P62280	40S ribosomal protein S11 GN=RPS11 PE=1 SV=3	0.59	1
1423	O00148	ATP-dependent RNA helicase DDX39A GN=DDX39A PE=1 SV=2	0.58	1
1424	P62995	Transformer-2 protein homolog beta GN=TRA2B PE=1 SV=1	0.57	1
1425	P56211	cAMP-regulated phosphoprotein 19 GN=ARPP19 PE=1 SV=2	0.55	1
1426	Q9UH36	SRR1-like protein GN=SRRD PE=2 SV=1	0.54	1
1427	Q92820	Gamma-glutamyl hydrolase GN=GGH PE=1 SV=2	0.54	1
1428	Q6NUQ4	Transmembrane protein 214 GN=TMEM214 PE=1 SV=2	0.53	1
1429	O43505	N-acetyllactosaminide beta-1,3-N-acetylglucosaminyltransferase GN=B3GNT1 PE=1 SV=1	0.53	1
1430	O43681	ATPase ASNA1 GN=ASNA1 PE=1 SV=2	0.51	1
1431	O60493	Sorting nexin-3 GN=SNX3 PE=1 SV=3	0.5	1
1432	Q96C86	m7GpppX diphosphatase GN=DCPS PE=1 SV=2	0.5	1
1433	Q29836	HLA class I histocompatibility antigen, B-67	0.49	1

		alpha chain GN=HLA-B PE=1 SV=1		
1434	Q13045	Protein flightless-1 homolog GN=FLII PE=1 SV=2	0.49	1
1435	Q9NUP9	Protein lin-7 homolog C GN=LIN7C PE=1 SV=1	0.49	1
1436	Q14444	Caprin-1 GN=CAPRIN1 PE=1 SV=2	0.48	1
1437	Q9H074	Polyadenylate-binding protein-interacting protein 1 GN=PAIP1 PE=1 SV=1	0.48	1
1438	P55008	Allograft inflammatory factor 1 GN=AIF1 PE=1 SV=1	0.47	1
1439	Q8IZ07	Ankyrin repeat domain-containing protein 13A GN=ANKRD13A PE=1 SV=3	0.47	1
1440	P36915	Guanine nucleotide-binding protein-like 1 GN=GNL1 PE=1 SV=2	0.46	1
1441	P06310	Ig kappa chain V-II region RPMI 6410 PE=4 SV=1	0.46	11
1442	Q8WZA9	Immunity-related GTPase family Q protein GN=IRGQ PE=1 SV=1	0.46	1
1443	P62899	60S ribosomal protein L31 GN=RPL31 PE=1 SV=1	0.46	1
1444	P46060	Ran GTPase-activating protein 1 GN=RANGAP1 PE=1 SV=1	0.46	1
1445	Q96GX5	Serine/threonine-protein kinase greatwall GN=MASTL PE=1 SV=1	0.46	1
1446	Q96FJ2	Dynein light chain 2, cytoplasmic GN=DYNLL2 PE=1 SV=1	0.45	1
1447	P62701	40S ribosomal protein S4, X isoform GN=RPS4X PE=1 SV=2	0.44	1
1448	P29034	Protein S100-A2 GN=S100A2 PE=1 SV=3	0.43	2
1449	Q9NYB0	Telomeric repeat-binding factor 2-interacting protein 1 GN=TERF2IP PE=1 SV=1	0.43	1
1450	Q5SRE5	Nucleoporin NUP188 homolog GN=NUP188 PE=1 SV=1	0.42	1

1451	O43583	Density-regulated protein GN=DENR PE=1 SV=2	0.41	1
1452	Q9Y224	UPF0568 protein C14orf166 GN=C14orf166 PE=1 SV=1	0.41	1
1453	P01621	Ig kappa chain V-III region NG9 (Fragment) PE=1 SV=1	0.4	18
1454	Q3MJ13	WD repeat-containing protein 72 GN=WDR72 PE=2 SV=2	0.39	1
1455	Q9GZP4	PITH domain-containing protein 1 GN=PITHD1 PE=1 SV=1	0.38	1
1456	Q13177	Serine/threonine-protein kinase PAK 2 GN=PAK2 PE=1 SV=3	0.38	1
1457	P20783	Neurotrophin-3 GN=NTF3 PE=1 SV=1	0.38	1
1458	Q15785	Mitochondrial import receptor subunit TOM34 GN=TOMM34 PE=1 SV=2	0.37	1
1459	Q9UBQ0	Vacuolar protein sorting-associated protein 29 GN=VPS29 PE=1 SV=1	0.37	1
1460	Q16666	Gamma-interferon-inducible protein 16 GN=IFI16 PE=1 SV=3	0.36	2
1461	Q92734	Protein TFG GN=TFG PE=1 SV=2	0.36	1
1462	P39019	40S ribosomal protein S19 GN=RPS19 PE=1 SV=2	0.36	1
1463	O94823	Probable phospholipid-transporting ATPase VB GN=ATP10B PE=2 SV=2	0.36	1
1464	Q9UNF0	Protein kinase C and casein kinase substrate in neurons protein 2 GN=PACSIN2 PE=1 SV=2	0.35	1
1465	Q8NAB2	Kelch repeat and BTB domain-containing protein 3 GN=KBTBD3 PE=2 SV=2	0.35	1
1466	P41240	Tyrosine-protein kinase CSK GN=CSK PE=1 SV=1	0.34	1
1467	Q17RC7	Exocyst complex component 3-like protein 4 GN=EXOC3L4 PE=2 SV=2	0.34	1
1468	P16885	1-phosphatidylinositol 4,5-bisphosphate phosphodiesterase gamma-2 GN=PLCG2	0.34	1

		PE=1 SV=4		
1469	P49207	60S ribosomal protein L34 GN=RPL34 PE=1 SV=3	0.34	1
1470	P29350	Tyrosine-protein phosphatase non-receptor type 6 GN=PTPN6 PE=1 SV=1	0.33	1
1471	Q9UK76	Hematological and neurological expressed 1 protein GN=HN1 PE=1 SV=3	0.33	1
1472	P61328	Fibroblast growth factor 12 GN=FGF12 PE=1 SV=1	0.32	1
1473	Q96IV0	Peptide-N(4)-(N-acetyl-beta-glucosaminyl)asparagine amidase GN=NGLY1 PE=1 SV=1	0.32	1
1474	Q9NYC9	Dynein heavy chain 9, axonemal GN=DNAH9 PE=1 SV=3	0.32	1
1475	O00273	DNA fragmentation factor subunit alpha GN=DFFA PE=1 SV=1	0.32	1
1476	P18621	60S ribosomal protein L17 GN=RPL17 PE=1 SV=3	0.31	1
1477	O95758	Polypyrimidine tract-binding protein 3 GN=PTBP3 PE=1 SV=2	0.31	1
1478	Q00688	Peptidyl-prolyl cis-trans isomerase FKBP3 GN=FKBP3 PE=1 SV=1	0.31	1
1479	O00487	26S proteasome non-ATPase regulatory subunit 14 GN=PSMD14 PE=1 SV=1	0.3	1
1480	O15085	Rho guanine nucleotide exchange factor 11 GN=ARHGEF11 PE=1 SV=1	0.29	1
1481	P07814	Bifunctional glutamate/proline--tRNA ligase GN=EPRS PE=1 SV=5	0.29	1
1482	Q7LG56	Ribonucleoside-diphosphate reductase subunit M2 B GN=RRM2B PE=1 SV=1	0.28	1
1483	Q9Y295	Developmentally-regulated GTP-binding protein 1 GN=DRG1 PE=1 SV=1	0.28	1
1484	P34949	Mannose-6-phosphate isomerase GN=MPI PE=1 SV=2	0.27	1

1485	Q6PJP8	DNA cross-link repair 1A protein GN=DCLRE1A PE=1 SV=3	0.27	1
1486	P36873	Serine/threonine-protein phosphatase PP1-gamma catalytic subunit GN=PPP1CC PE=1 SV=1	0.27	4
1487	Q04206	Transcription factor p65 GN=RELA PE=1 SV=2	0.27	1
1488	P06132	Uroporphyrinogen decarboxylase GN=UROD PE=1 SV=2	0.27	1
1489	Q96B23	Uncharacterized protein C18orf25 GN=C18orf25 PE=1 SV=2	0.26	1
1490	Q9UJX4	Anaphase-promoting complex subunit 5 GN=ANAPC5 PE=1 SV=2	0.26	1
1491	P29144	Tripeptidyl-peptidase 2 GN=TPP2 PE=1 SV=4	0.26	1
1492	A3KMH1	von Willebrand factor A domain-containing protein 8 GN=VWA8 PE=1 SV=2	0.25	1
1493	Q8ND23	Leucine-rich repeat-containing protein 16B GN=LRRC16B PE=2 SV=2	0.25	1
1494	Q9H5H4	Zinc finger protein 768 GN=ZNF768 PE=1 SV=2	0.25	1
1495	Q8IYT4	Katanin p60 ATPase-containing subunit A-like 2 GN=KATNAL2 PE=2 SV=3	0.25	2
1496	O95359	Transforming acidic coiled-coil-containing protein 2 GN=TACC2 PE=1 SV=3	0.24	1
1497	Q8N3Y7	Epidermal retinol dehydrogenase 2 GN=SDR16C5 PE=2 SV=2	0.24	1
1498	Q8NBF2	NHL repeat-containing protein 2 GN=NHLRC2 PE=1 SV=1	0.23	1
1499	P11171	Protein 4.1 GN=EPB41 PE=1 SV=4	0.23	1
1500	Q9NP79	Vacuolar protein sorting-associated protein VTA1 homolog GN=VTA1 PE=1 SV=1	0.2	1
1501	Q99466	Neurogenic locus notch homolog protein 4 GN=NOTCH4 PE=1 SV=2	0.19	1
1502	P55061	Bax inhibitor 1 GN=TMBIM6 PE=1 SV=2	0.19	0

1503	P42025	Beta-centractin GN=ACTR1B PE=1 SV=1	0.19	2
1504	Q07075	Glutamyl aminopeptidase GN=ENPEP PE=1 SV=3	0.19	1
1505	Q9BRP8	Partner of Y14 and mago GN=WIBG PE=1 SV=1	0.19	1
1506	Q8N8A2	Serine/threonine-protein phosphatase 6 regulatory ankyrin repeat subunit B GN=ANKRD44 PE=1 SV=3	0.16	0
1507	Q14153	Protein FAM53B GN=FAM53B PE=1 SV=2	0.14	0
1508	Q9C0D0	Phosphatase and actin regulator 1 GN=PHACTR1 PE=2 SV=3	0.12	0

Supplemental Table S4. The list of unique proteins identified from DDA (FDR < 1%, 95% peptide confidence level) in the spectral library used to process SWATH data.
